# m6A-dependent regulation of DGUOK-AS1 by RBM15 and HNRNPH1 in lung adenocarcinoma

**DOI:** 10.1016/j.isci.2026.117078

**Published:** 2026-08-07

**Authors:** Menghao Yang, Jiaen Wu, Youjie Li, Hui Li, Hongrong Zhang, Yan Liang, Qin Wang, Pingyu Wang, Yuan Yu, Guangbin Sun, Jiankai Feng, Wenwen Liu, Shuyang Xie, Hongfang Sun

**Affiliations:** 1 Department of Biochemistry and Molecular Biology, Binzhou Medical University, Yantai, Shandong, China; 2 Department of Laboratory Medicine, Yantai Affiliated Hospital of Binzhou Medical University, Yantai, Shandong, China; 3 Department of Endocrinology and Metabolism, Cleveland Clinic, Cleveland, OH, USA

**Keywords:** LUAD, DGUOK-AS1, RBM15, PRMT5, HNRNPH1, m6A

## Abstract

Lung adenocarcinoma (LUAD) remains a leading cause of cancer-related death, underscoring the need for an improved molecular understanding. This study investigated the regulatory mechanism of the long non-coding RNA deoxyguanosine kinase antisense RNA 1 (DGUOK-AS1) in LUAD. DGUOK-AS1 was significantly upregulated in LUAD cells and serum samples, and its elevated expression showed a preliminary association with LUAD. Functional experiments demonstrated that DGUOK-AS1 promoted LUAD proliferation and migration both *in vitro* and *in vivo*, partly by acting as a competing endogenous RNA for miR-2467-5p to modulate PRMT5 expression. Mechanistically, RNA-binding motif protein 15 (RBM15) enhanced DGUOK-AS1 stability through m6A modification, which in turn enabled heterogeneous nuclear ribonucleoprotein H1 (HNRNPH1) binding in an m6A-dependent manner via its RNA recognition motif 3 (RRM3) domain, promoting degradation. RBM15 knockdown attenuated the malignant phenotype through the miR-2467-5p/PRMT5 axis. These findings reveal an m6A-dependent mechanism governing DGUOK-AS1 stability and provide insights into its contribution to LUAD progression.

## Highlights

• DGUOK-AS1 is upregulated in LUAD and shows a preliminary clinical association
• RBM15 and HNRNPH1 oppositely regulate DGUOK-AS1 stability via m6A modification
• DGUOK-AS1 promotes LUAD growth partly through the miR-2467-5p/PRMT5 axis
• HNRNPH1 binds DGUOK-AS1 in an m6A-dependent manner via its RRM3 domain

## Graphical abstract

**Figure:**
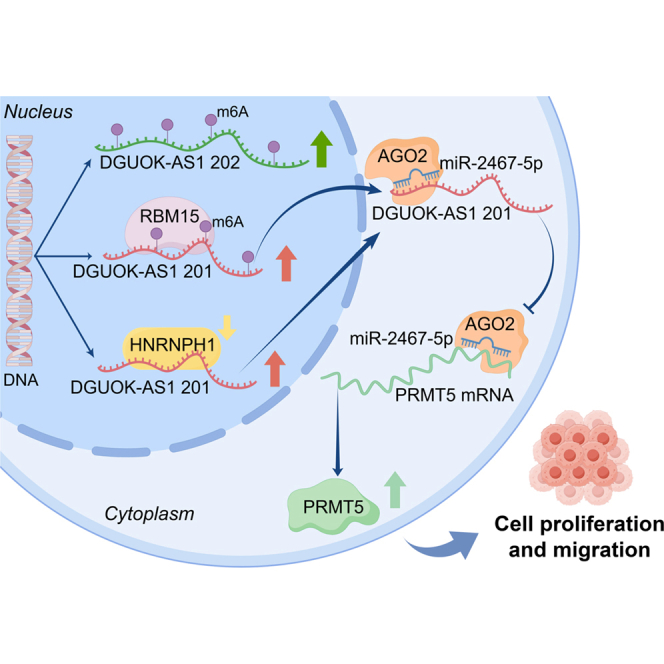

## Teaser

Nucleic acids; Protein; Biochemical mechanism

## Introduction

Non-small cell lung cancer (NSCLC) accounts for a relatively large proportion of lung cancer,^1^^,^^2^^,^^3^^,^^4^ mainly including lung adenocarcinoma (LUAD) and lung squamous cell carcinoma (LUSC). NSCLC has consistently been considered a serious threat to human health and life, characterized by its high proliferation, invasive tendencies, and metastatic capabilities.^5^^,^^6^ Consequently, it is essential and significant to investigate the molecular mechanism underlying the progression of NSCLC.

The interactions between long non-coding RNAs (LncRNAs) and microRNAs (miRNAs) play a crucial role in collectively regulating gene expression and influencing various physiological manifestations of tumor cells.^7^^,^^8^^,^^9^ We identified a LncRNA, deoxyguanosine kinase antisense RNA 1 (DGUOK-AS1) whose expression was upregulated in NSCLC. It has been reported that DGUOK-AS1 can promote cell proliferation, migration, invasion, and angiogenesis, exerting a tumorigenic role in breast cancer, cervical squamous cell carcinoma, liver cancer, and LUSC.^10^^,^^11^^,^^12^^,^^13^ However, the molecular mechanisms underlying the role of DGUOK-AS1 in LUAD remain unclear.

The N6-methyladenosine (m6A) modification stands as the most prevalent modification on mRNA and ncRNA, playing a pivotal role in various biological processes which include splicing, nuclear transportation, stabilization, degradation, and translation of RNAs.^14^^,^^15^^,^^16^ The m6A modifications are added by the “writer” proteins, which mainly include METTL3 and METTL14,^17^^,^^18^^,^^19^ while removed by “erasers” such as FTO and ALKBH5.^20^^,^^21^ The m6A “reader” proteins consist of the YTH family proteins,^22^ IGF2BPs,^23^ and others.^24^^,^^25^^,^^26^ RNA-binding motif protein 15 (RBM15), belonging to the m6A “writer” proteins, functions predominantly by associating with WTAP and METTL3 to carry out m6A methylation modifications on RNAs.^27^ Research findings indicate that RBM15 plays a role in facilitating the initiation and progression of laryngeal squamous cell carcinoma through the maintenance of TMBIM6 mRNA stability.^28^ Moreover, RBM15-mediated m6A modification could potentially promote the progression of hepatocellular carcinoma (HCC) and the osimertinib resistance of LUAD.^29^^,^^30^ However, there are few studies on the effects of RBM15 on LncRNAs.

LncRNA performs its biological function mainly through interaction with RNA-binding proteins (RBPs). Studies have reported that heterogeneous nuclear ribonucleoprotein H1 (HNRNPH1) can regulate exon recognition and splice-site selection.^31^ Moreover, it can interact with proteins and RNAs, contributing to stabilize RNA and facility translation processes.^32^^,^^33^ However, the functional role of HNRNPH1 and its interaction with DGUOK-AS1 remain poorly understood in LUAD.

This study identified DGUOK-AS1 as an upregulated lncRNA in LUAD and assessed its potential as a diagnostic biomarker for NSCLC. Moreover, we found that DGUOK-AS1 promotes the growth of LUAD cells *in vitro* and *in vivo*, partially via the miR-2467-5p/PRMT5 axis. We also demonstrated that RBM15-induced m6A modification stabilizes and increases DGUOK-AS1 expression levels. Additionally, the interaction between DGUOK-AS1 and HNRNPH1 leads to the degradation of this LncRNA. Taken together, these findings suggest that DGUOK-AS1 could be a valuable biomarker and therapeutic target for LUAD.

## Results

### The expression of DGUOK-AS1 was increased in NSCLC

**Figure 1:**
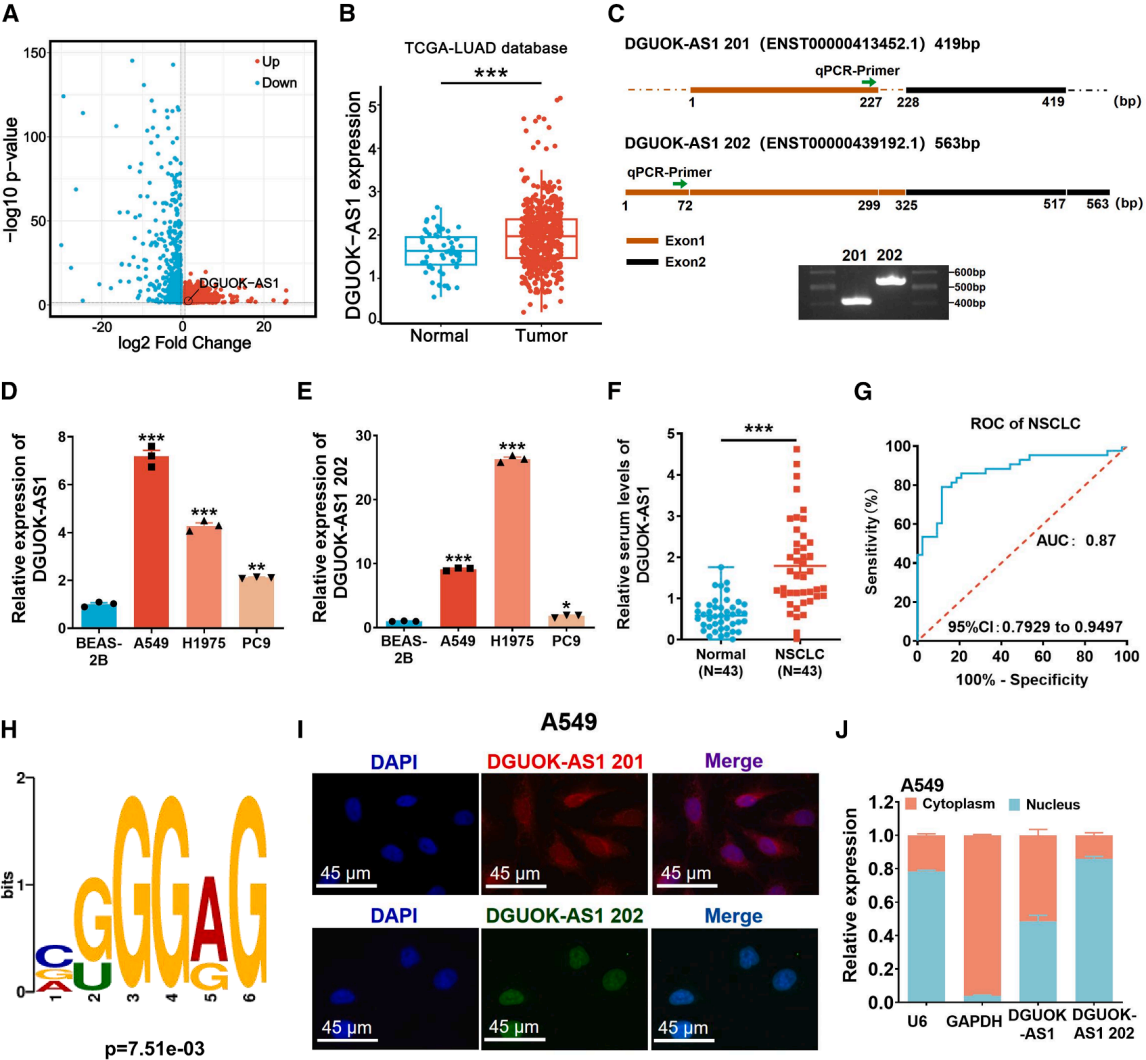
DGUOK-AS1 is upregulated in lung adenocarcinoma (A) Volcano plot shows the LncRNA differential analysis in LUAD dataset of The Cancer Genome Atlas (TCGA). (B) Differential expression analysis of DGUOK-AS1 in the TCGA-LUAD dataset (∗∗∗*p* < 0.001) (normal: *n* = 59; NSCLC: *n* = 483). (C) Diagram of RT-qPCR primers for DGUOK-AS1 201/202 and the gel image of full-length PCR products. (D and E) The expression of DGUOK-AS1 201/202 in BEAS-2B, A549, H1975, and PC-9 cells (*n* = 3, ∗*p* < 0.05, ∗∗*p* < 0.01, ∗∗∗*p* < 0.001). (F) DGUOK-AS1 expression in serum samples from 43 pairs of healthy individuals and NSCLC patients (*n* = 3, ∗∗∗*p* < 0.001; mean ± SD = 0.6122 ± 0.3887). (G) The receiver-operating curve (ROC) analysis was used to evaluate the diagnostic performance of DGUOK-AS1 derived from serum sample data (normal: *n* = 43; NSCLC: *n* = 43). (H) The exosome sorting motif of DGUOK-AS1 201. (I) FISH analysis indicated subcellular location of DGUOK-AS1 (red) and DGUOK-AS1 202 (green) in A549 cells. Cell nuclei are stained with 4',6-diamidino-2-phenylindole (DAPI) (blue) (scale bars, 45 μm; *n* = 3). (J) The expression of DGUOK-AS1 201/202 in the cytoplasmic and nuclear of A549 cells (*n* = 4). Data in all graphs are shown as mean ± SEM based on three independent trials. For (A–J), *n* represents the number of independent biological replicates, unless otherwise specified (mice: *n* = number of animals). Statistical analysis was performed using a paired *t* test (F) or one-way ANOVA (D and E), followed by Tukey’s multiple comparison test.

In recent years, LncRNAs have been recognized as significant regulators of cellular functions.^34^ To investigate the potential mechanisms of LncRNAs in LUAD, we conducted an analysis of data from The Cancer Genome Atlas (TCGA) database and identified several LncRNAs exhibiting significantly different expression levels (Figure 1A). Among them, LncRNA DGUOK-AS1 shows higher expression levels in LUAD (Figure 1B). The data showed that two transcripts of DGUOK-AS1 were increased in LUAD (Table S1). To distinguish between the two transcripts of DGUOK-AS1, designated as 201 and 202, and the sizes of the two cloned DGUOK-AS1 transcripts are presented in Figure 1C. The successful cloning of the DGUOK-AS1 transcript was confirmed by Sanger sequencing (Table S2). The qPCR primers were specifically designed for the two DGUOK-AS1 transcripts (201 and 202) (Figure 1C).

We next evaluated the expression levels of the nine candidate lncRNAs. Our findings demonstrated significantly elevated levels of DGUOK-AS1 expression in the A549, H1975, and PC-9 cells compared to the BEAS-2B cells (Figure S1A; Figures 1D and 1E). Furthermore, we gathered serum samples from 43 pairs of lung cancer patients and healthy individuals for clinical examination. Quantitative real-time PCR (RT-qPCR) analysis revealed higher DGUOK-AS1 RNA levels in serum samples from NSCLC patients compared to healthy individuals (Figures S1B and1F). The receiver-operating curve (ROC) from RT-qPCR analysis shows that the detection of DGUOK-AS1 in serum has moderate sensitivity and specificity, with an area under the curve of 0.87 (Figure 1G). Among the 9 candidates examined (Figures S1A and S1B; Figures 1D–1F), DGUOK-AS1 showed the most consistent and pronounced upregulation across both serum and cell lines, and was therefore selected for further functional characterization. We next analyzed the exosome sorting motif of DGUOK-AS1 201 by MEME Suite (Figure 1H). Meanwhile, the distribution of DGUOK-AS1 201 in the cytoplasm and nucleus of A549 and H1975 cells, as determined by fluorescent *in situ* hybridization (FISH) and nuclear-cytoplasmic fractionation assays (Figures 1I and 1J; Figures S1C and S1D), with DGUOK-AS1 202 notably accumulating in the nucleus. The results showed that DGUOK-AS1 201 possesses the potential for uptake by exosomes. Our findings demonstrate that DGUOK-AS1 is upregulated in NSCLC and that serum DGUOK-AS1 represents a promising candidate biomarker for distinguishing NSCLC patients from healthy individuals.

### DGUOK-AS1 201 facilitates the proliferation and migration of LUAD cells

**Figure 2:**
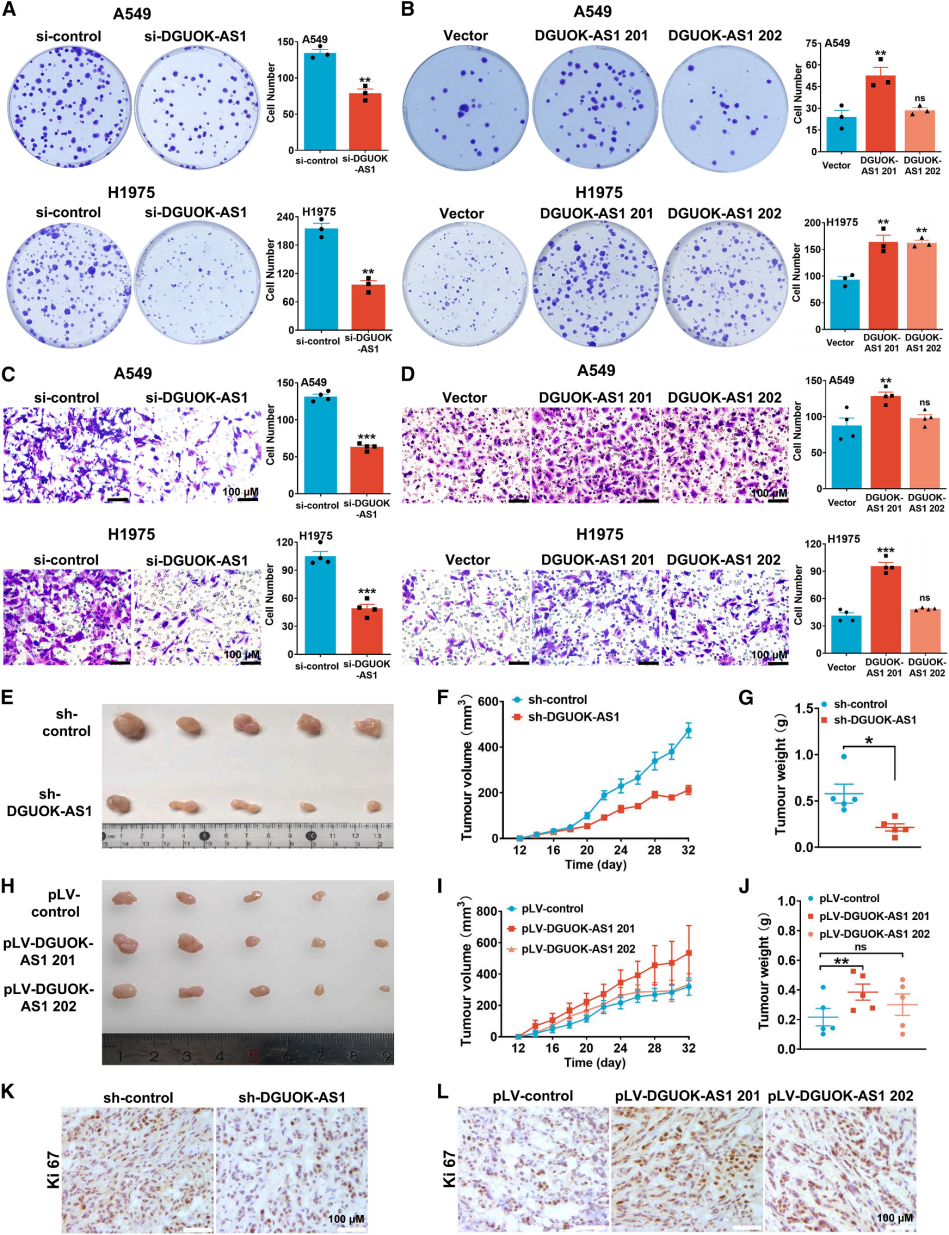
DGUOK-AS1 promotes the growth of lung adenocarcinoma cells (A) Colony formation assay was performed to measure the cell proliferation of A549 and H1975 cells transfected with DGUOK-AS1 siRNA or scrambled controls (*n* = 3, ∗∗*p* < 0.01). (B) Cell proliferation analysis of A549 and H1975 cells transfected with DGUOK-AS1 201 and DGUOK-AS1 202 overexpression vectors or control vector (*n* = 3, ∗∗*p* < 0.01; ns, not significant). (C) The migration ability of A549 and H1975 cells transfected with DGUOK-AS1 siRNA or scrambled controls was detected by Transwell assay (scale bars, 100 μm; *n* = 4, ∗∗∗*p* < 0.001). (D) Transwell migration assays of A549 and H1975 cells transfected with DGUOK-AS1 201 and DGUOK-AS1 202 overexpression vectors or control vector (scale bars, 100 μm; *n* = 4, ∗∗*p* < 0.01, ∗∗∗*p* < 0.001; ns, not significant). (E) Representative images of the xenograft tumors by subcutaneous injection of A549 cells stably transfected with sh-DGUOK-AS1 or sh-control (*n* = 5 mice per group). (H) Images of the tumors formed by stable A549 cells infected with lentivirus expressing DGUOK-AS1 201, DGUOK-AS1 202, or control lentivirus (*n* = 5 mice per group). (F, G, I, and J) Growth curves and weight of tumors in subcutaneous xenografts from knockdown or overexpression groups (*n* = 5, ∗*p* < 0.05, ∗∗*p* < 0.01; ns, not significant). (K and L) The Ki67 expression was determined by immunohistochemical assay in the subcutaneous xenograft models (scale bars, 100 μm; *n* = 3). Data in all graphs are shown as mean ± SEM based on three independent trials. For (A–L), *n* represents the number of independent biological replicates, unless otherwise specified (mice: *n* = number of animals). Statistical analysis was performed using a paired *t* test (A, C, G, and J) or one-way ANOVA (B and D), followed by Tukey’s multiple comparison test.

We constructed overexpression plasmids of DGUOK-AS1 201 and DGUOK-AS1 202, and designed small interfering RNAs (siRNAs) targeting their shared region to alter the expression levels of DGUOK-AS1 in LUAD cells (Figures S2A and S2B). Cell proliferation assays (CCK-8) showed that the downregulation of DGUOK-AS1 reduced proliferation of A549 and H1975 cells (Figure S2C), whereas the upregulation of DGUOK-AS1 enhanced proliferation of A549, H1975, and PC-9 cells (Figure S2D), with DGUOK-AS1 201 exhibiting a more pronounced effect than DGUOK-AS1 202. Consistent with these findings, knockdown and overexpression of DGUOK-AS1 significantly reduced and increased the colony-forming ability of A549 and H1975 cells compared to the respective control cells (Figures 2A and 2B). Moreover, the Transwell assay and wound healing findings indicated that the downregulation of DGUOK-AS1 hindered cell migration (Figure 2C; Figure S2E), while the upregulation of DGUOK-AS1 201 promoted the migration of A549, H1975, and PC-9 cells (Figure 2D; Figures S2F and S2G), with DGUOK-AS1 202 demonstrating a slight increase in migratory capacity.

To investigate the effect of DGUOK-AS1 on tumorigenicity *in vivo*, we subcutaneously injected A549 cells with either stable DGUOK-AS1 interference or high expression into 5-week-old female nude mice. The results indicated that A549 cells with reduced DGUOK-AS1 expression formed smaller tumors in nude mice and displayed slower growth rates (Figures 2E–2G). Compared with DGUOK-AS1 202, A549 cells with elevated levels of DGUOK-AS1 201 developed larger and more rapidly growing tumors (Figures 2H–2J). Immunohistochemical (IHC) analysis showed that the Ki67 level was reduced in the sh-DGUOK-AS1 group, while it was increased in the DGUOK-AS1-overexpressing groups, especially in the DGUOK-AS1 201 group (Figures 2K and 2L). These data suggest that DGUOK-AS1 201 exhibits a more significant capacity to promote the proliferation and migration of the LUAD cells.

### DGUOK-AS1 201 targets miR-2467-5p, influencing cell proliferation and migration

**Figure 3:**
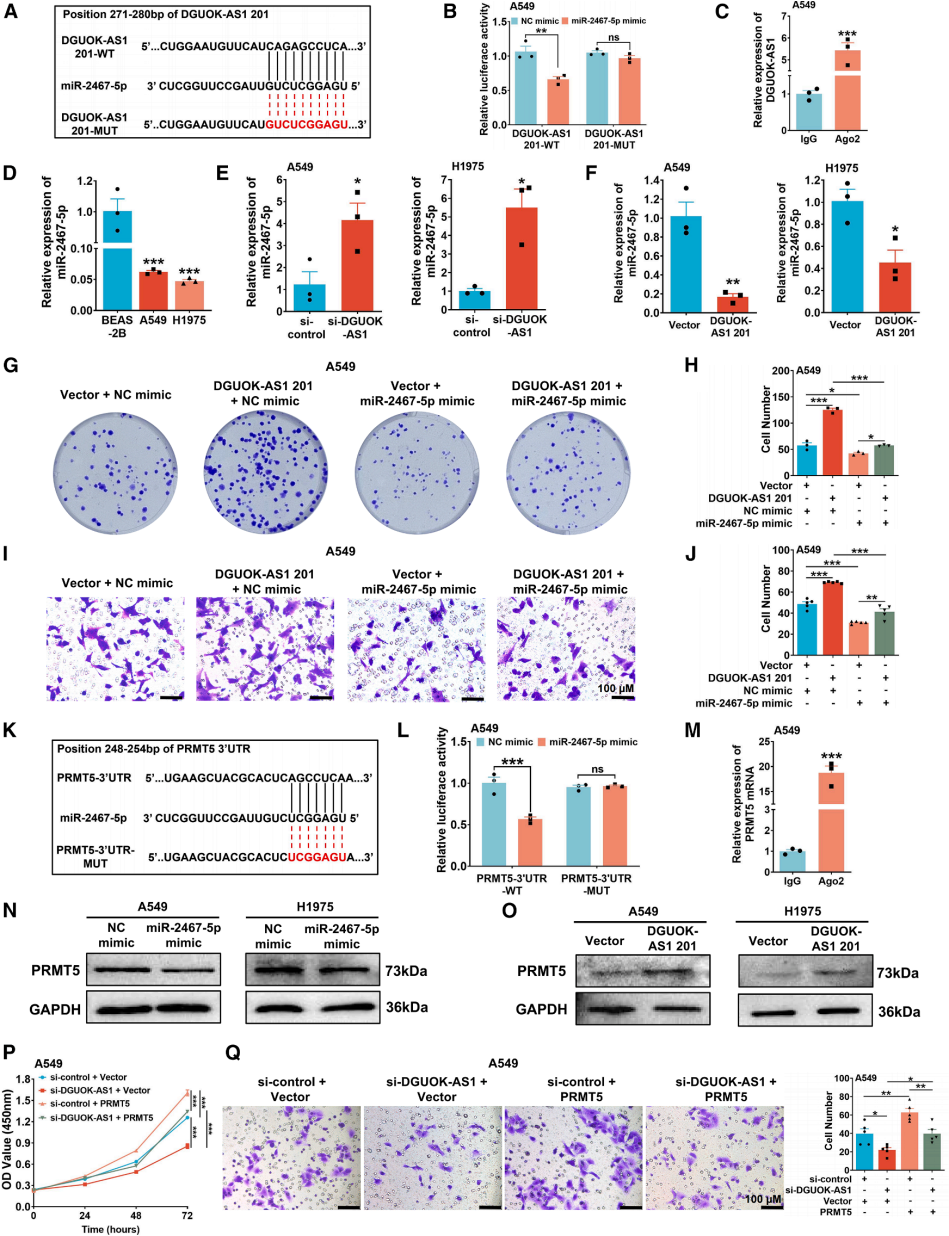
DGUOK-AS1 201 and miR-2467-5p regulated PRMT5 expression (A) Predicted binding sites of miR-2467-5p on wild-type and mutant DGUOK-AS1 201 sequences. (B) Dual-luciferase reporter assay in A549 cells co-transfected with wild-type (WT) or mutant (MUT) DGUOK-AS1 201 and miR-2467-5p mimics (*n* = 3, ∗∗*p* < 0.01; ns, not significant). (C) RIP-qPCR analysis of DGUOK-AS1 enrichment on Ago2 relative to IgG in A549 cells (*n* = 3, ∗∗∗*p* < 0.001). (D) miR-2467-5p expression in BEAS-2B, A549, and H1975 cells was determined by RT-qPCR (*n* = 3, ∗∗∗*p* < 0.001). (E and F) miR-2467-5p levels in A549 and H1975 cells following DGUOK-AS1 knockdown or DGUOK-AS1 201 overexpression (*n* = 3, ∗*p* < 0.05, ∗∗*p* < 0.01). (G–J) Colony formation and Transwell assays assessing proliferation and migration of A549 cells co-transfected with DGUOK-AS1 201 overexpression vector and miR-2467-5p mimics (G and H: *n* = 3; I and J: scale bars, 100 μm; *n* = 5; ∗*p* < 0.05, ∗∗*p* < 0.01, ∗∗∗*p* < 0.001). (K) Schematic of the predicted binding sites between miR-2467-5p and the wild-type or mutant 3′ UTR of PRMT5 mRNA. (L) Dual-luciferase reporter assay validating the interaction between miR-2467-5p and the PRMT5 3′ UTR in A549 cells (*n* = 3, ∗∗∗*p* < 0.001; ns, not significant). (M) RIP-qPCR analysis of PRMT5 mRNA enrichment using anti-Ago2 antibody or IgG control (*n* = 3, ∗∗∗*p* < 0.001). (N) PRMT5 protein expression in A549 and H1975 cells transfected with NC mimic or miR-2467-5p mimics. (O) PRMT5 protein levels in A549 and H1975 cells overexpressing DGUOK-AS1 201. (P and Q) CCK-8 and Transwell assays of proliferation and migration in A549 cells co-transfected with PRMT5 overexpression vector and DGUOK-AS1 siRNA (P: *n* = 3; Q: scale bars, 100 μm; *n* = 5; ∗*p* < 0.05, ∗∗*p* < 0.01, ∗∗∗*p* < 0.001). The data are shown as the mean ± SEM based on three independent trials. For (A–Q), *n* represents the number of independent biological replicates, unless otherwise specified. Statistical analysis was performed using a paired *t* test (C, E, F, and M), a one-way ANOVA (D, H, J, and Q), or two-way ANOVA (B and L), followed by Tukey’s multiple comparison test.

We identified the main subcellular location of DGUOK-AS1 201 in the cytoplasm of A549 and H1975 cells based on the previous results. To elucidate the molecular mechanisms through which DGUOK-AS1 facilitates the progression of LUAD, we hypothesize that DGUOK-AS1 201 may function in the cytoplasm as a molecular “sponge” for specific miRNAs, thereby facilitating oncogenic processes. We used miRDB to predict miRNAs that may interact with DGUOK-AS1 201 (Table S3). Among the predicted miRNAs, the top 4 candidates by prediction score were examined for expression changes upon DGUOK-AS1 201 knockdown or overexpression. miR-2467-5p showed the strongest regulation and was therefore selected for further study (Figures S3A and S3B). The potential DGUOK-AS1 201 binding sites of miR-2467-5p were predicted in the TargetScan database (Figure 3A). Dual luciferase reporter assay revealed that overexpression of the miR-2467-5p mimic diminished the luciferase activity of DGUOK-AS1 201-wild type (WT) but not that of DGUOK-AS1 201-mutant (MUT) (Figure 3B; Figure S3C). In addition, the anti-Ago2 RNA immunoprecipitation (RIP) assay also confirmed that miR-2467-5p was a target of DGUOK-AS1 201 in A549 and H1975 cells (Figure 3C; Figure S3D).

Compared with BEAS-2B cells, the expression levels of endogenous miR-2467-5p were significantly lower in the A549 and H1975 cells (Figure 3D), which negatively correlated with the expression levels of DGUOK-AS1 201. Furthermore, the downexpression of DGUOK-AS1 201 results in an increase in the expression levels of miR-2467-5p, while a reduction of miR-2467-5p expression levels is observed under the opposite conditions (Figures 3E and 3F). Next, we performed rescue experiments in A549 cells to explore whether DGUOK-AS1 201 promotes progression of LUAD by interacting with miR-2467-5p. The results showed that miR-2467-5p mimics could partially diminish the DGUOK-AS1 201 overexpression-induced increase in proliferation and migration of A549 cells (Figures 3G–3J; Figures S3E and S3F). Additionally, restoration of miR-2467-5p could inhibit epithelial-mesenchymal transition (EMT), which was promoted by DGUOK-AS1 201, as shown by increased E-cadherin and decreased vimentin (Figure S3G). The results mentioned suggest that DGUOK-AS1 201 might act as a “sponge” binding miR-2467-5p and affecting cell proliferation and migration.

### PRMT5 is a downstream target of the DGUOK-AS1 201/miR-2467-5p pathway

To elucidate the underlying molecular mechanism of the DGUOK-AS1 201/miR-2467-5p axis in regulating lung cancer cell proliferation, we used bioinformatic tools (TargetScan and miRWalk) to predict target genes of miR-2467-5p. As shown in Figure 3K, binding sequences of miR-2467-5p were identified in the 3′UTR of PRMT5 mRNA. Subsequently, the dual-luciferase reporter assays demonstrated a significant decrease in fluorescence intensity in the co-transfection group of miR-2467-5p mimic and the WT PRMT5 mRNA 3′UTR (Figure 3L). Moreover, the RIP experiments showed significant enrichment of PRMT5 mRNA in the Ago2 antibody group (Figure 3M), suggesting that miR-2467-5p may bind to the PRMT5 mRNA 3′UTR. Additionally, western blot analysis revealed that miR-2467-5p could downregulate the expression of PRMT5 in A549 and H1975 cells (Figure 3N), while the overexpression of DGUOK-AS1 201 led to increased levels of PRMT5 protein (Figure 3O). These results demonstrate that PRMT5 is a downstream target of the DGUOK-AS1 201/miR-2467-5p axis.

Some studies have demonstrated that knockdown of PRMT5 significantly suppressed tumor growth.^35^ We then reduced PRMT5 protein levels in A549 and H1975 cells (Figure S4A) and analyzed the proliferation and migration ability of the LUAD cells. The findings demonstrated that the downregulation of PRMT5 significantly impeded the proliferation and migration of A549 and H1975 cells (Figures S4B–S4D). Moreover, we confirmed that overexpression of PRMT5 could promote the proliferation and migration of A549 cells by CCK-8, colony formation, and Transwell (Figures S4E–S4H). The rescue assay results showed that PRMT5 overexpression significantly counteracted the inhibition of cell proliferation and migration in A549 cells, which was induced by the DGUOK-AS1 knockdown (Figures 3P and 3Q; Figure S4I). Restoration of PRMT5 may facilitate EMT, which was inhibited by the downregulation of DGUOK-AS1, as evidenced by the reduction of E-cadherin and the upregulation of vimentin (Figure S4J). We propose that DGUOK-AS1 acts as a molecular “sponge” for miR-2467-5p, preventing it from binding to the 3′UTR of PRMT5 mRNA. This may result in elevated PRMT5 expression, potentially enhancing cell proliferation and migration.

### RBM15 interacts with DGUOK-AS1 201 and upregulates its m6A modification

**Figure 4:**
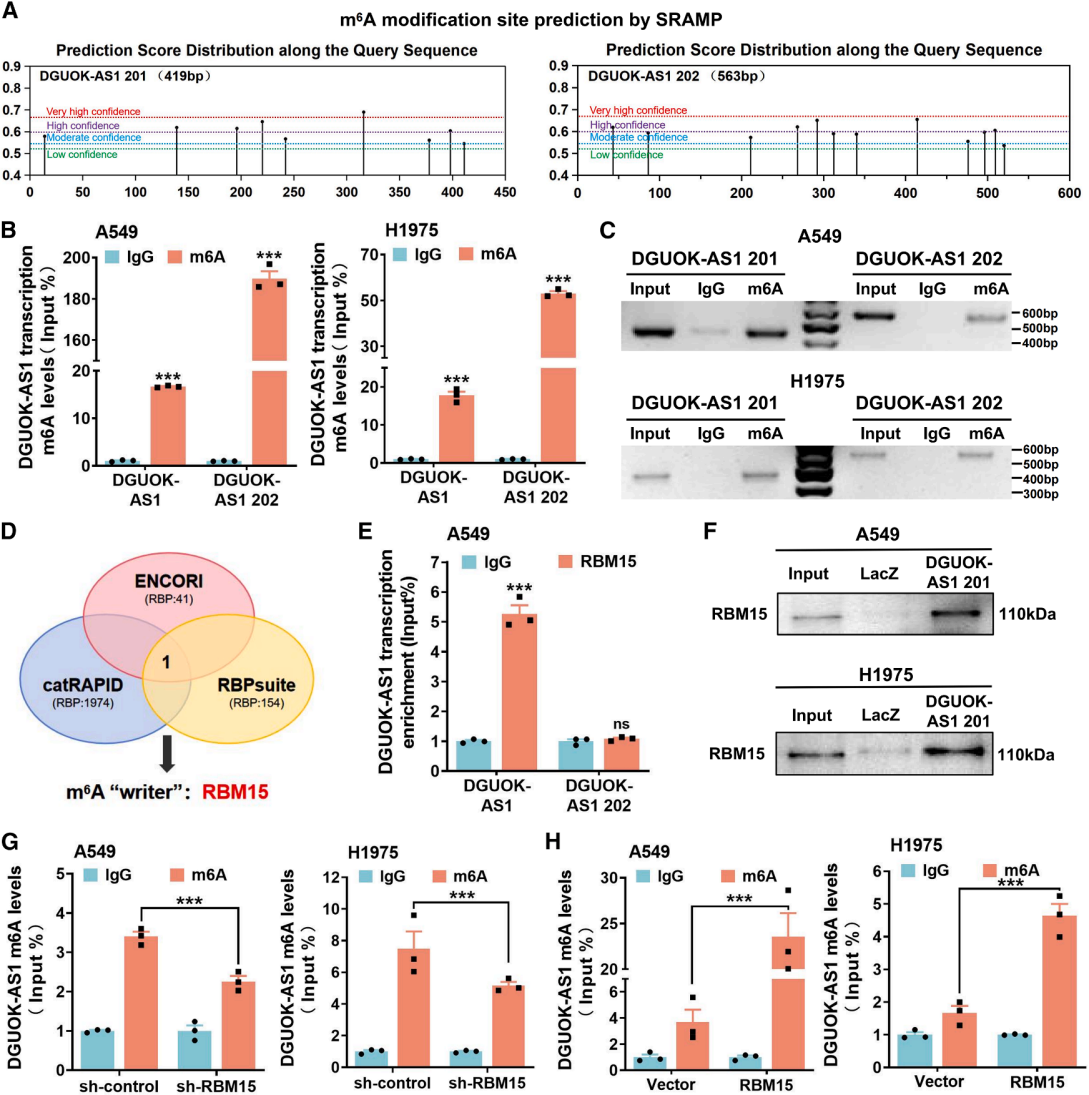
RBM15 interacts with DGUOK-AS1 201 and regulates its m6A modification (A) The m6A sites of DGUOK-AS1 201 and 202 predicted by SRAMP database. (B) MeRIP-qPCR assay was utilized to detect m6A enrichment of DGUOK-AS1 in A549 and H1975 cells (*n* = 3, ∗∗∗*p* < 0.001). (C) Agarose gel images showed the products of MeRIP-PCR using full-length primers. (D) Predicted binding proteins of DGUOK-AS1 201 using catRAPID, RBPsuite, and ENCORI and the one overlapping binding protein (m6A “writer”: RBM15). (E) RIP-qPCR assays using anti-RBM15 antibodies demonstrated that RBM15 specifically interacts with DGUOK-AS1 201 in A549 cells (*n* = 3, ∗∗∗*p* < 0.001; ns, not significant). (F) Western blotting was conducted to assess the levels of RBM15 enriched by LacZ probe and DGUOK-AS1 201 probe in A549 and H1975 cells. (G and H) The m6A enrichment of DGUOK-AS1 was measured after knocking down or overexpressing RBM15 by MeRIP-qPCR assay (*n* = 3, ∗∗∗*p* < 0.001). Data are displayed as the mean ± SEM based on three independent trials. For (A–H), *n* represents the number of independent biological replicates, unless otherwise specified. Statistical analysis was performed using a two-way ANOVA (B, E, G, and H) followed by Tukey’s multiple comparison test.

The m6A modification is crucial for the stability and degradation of LncRNAs in eukaryotic cells.^36^^,^^37^ To explore the role of m6A in regulating DGUOK-AS1 in LUAD, we first predicted m6A sites on DGUOK-AS1 201 and 202 by using SRAMP tools (Figure 4A; Figure S5A). Methylated RNA immunoprecipitation-quantitative PCR (MeRIP-qPCR) analysis in A549 and H1975 cells verified m6A modification on DGUOK-AS1, showing significant enrichment with the anti-m6A antibody compared to IgG (Figure 4B). Moreover, using the MeRIP products as templates, PCR was performed with full-length primers designed for DGUOK-AS1 201 and 202. Gel electrophoresis results demonstrated that the m6A modifications of DGUOK-AS1 201 and 202 were significantly enriched in A549 and H1975 cells (Figure 4C).

Next, we used catRAPID, ENCORI, and RBPsuite to predict RNA-binding proteins for DGUOK-AS1. RBM15 was predicted to bind to the DGUOK-AS1 201 sequence, which is the common sequence of the two DGUOK-AS1 transcripts (Figure 4D). Subsequently, we conducted RIP and RNA pull-down assays to confirm the interaction between DGUOK-AS1 201 and RBM15. The RIP-qPCR results indicated a significantly higher abundance of DGUOK-AS1 in the RBM15 antibody group compared to the IgG control group in A549 cells. However, there were no significant changes in the enrichment levels of DGUOK-AS1 202 (Figure 4E), suggesting that RBM15 primarily binds to DGUOK-AS1 201. In addition, a negative probe targeting LacZ and a full-length probe specific for DGUOK-AS1 201 were used for pull-down experiments. The results indicated that the levels of RBM15 protein detected in the DGUOK-AS1 probe group were significantly higher than those observed in the LacZ group (Figure 4F).

To examine its effect on the m6A modification of DGUOK-AS1, we modified the expression levels of RBM15 (Figures S5B–S5E). The MeRIP analyses revealed that the inhibition of RBM15 resulted in a decreased m6A enrichment on DGUOK-AS1 (Figure 4G). In contrast, the overexpression of RBM15 significantly increased m6A enrichment on DGUOK-AS1 (Figure 4H). These results suggested that RBM15 has been identified as a regulator of m6A modifications on DGUOK-AS1.

### Reduced RBM15 inhibits cellular proliferation and migration

**Figure 5:**
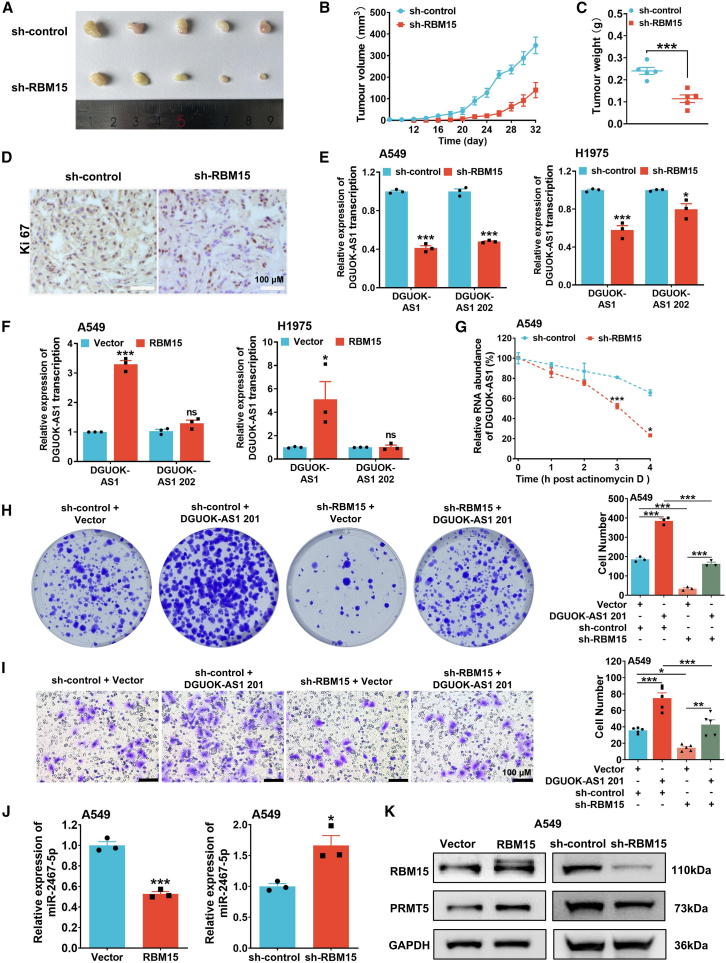
RBM15 upregulates the expression of DGUOK-AS1 201 and promotes the proliferation and migration of LUAD cells (A–C) Tumor volume and weight in nude mice injected with A549 cells stably transfected with sh-RBM15 or sh-control (*n* = 5 mice per group; ∗∗∗*p* < 0.001). (D) Immunohistochemistry (IHC) staining of Ki67 in subcutaneous xenograft tumors (scale bars, 100 μm). (E and F) RT-qPCR analysis of DGUOK-AS1 expression in A549 and H1975 cells upon RBM15 knockdown or overexpression (*n* = 3, ∗*p* < 0.05, ∗∗∗*p* < 0.001; ns, not significant). (G) Relative DGUOK-AS1 RNA levels measured by RT-qPCR following actinomycin D treatment (*n* = 3, ∗*p* < 0.05, ∗∗∗*p* < 0.001). (H) Colony formation assay of proliferation in A549 cells co-transfected with RBM15 knockdown lentivirus and DGUOK-AS1 201 overexpression vector (*n* = 3, ∗∗∗*p* < 0.001). (I) Transwell migration assay in RBM15-silenced A549 cells overexpressing DGUOK-AS1 201 (scale bars, 100 μm; *n* = 5, ∗*p* < 0.05, ∗∗*p* < 0.01, ∗∗∗*p* < 0.001). (J) RT-qPCR analysis of miR-2467-5p expression in A549 and H1975 cells upon RBM15 overexpression or knockdown (*n* = 3, ∗*p* < 0.05, ∗∗∗*p* < 0.001). (K) Protein levels of RBM15 and PRMT5 in A549 cells with RBM15 overexpression or knockdown. Data are presented as mean ± SEM from three independent experiments. For (A–K), *n* represents the number of independent biological replicates, unless otherwise specified (mice: *n* = number of animals). Statistical analysis was performed using a paired *t* test (C and J), a one-way ANOVA (H and I), or a two-way ANOVA (E, F, and G) followed by Tukey’s multiple comparison test.

We next explored the biological functions of RBM15 *in vitro* and *in vivo*. CCK-8 and colony formation assays showed that knockdown of RBM15 significantly inhibited cell proliferation in A549 and H1975 cells (Figures S5F and S5G). Further, Transwell and wound healing assays demonstrated that RBM15 knockdown diminished the migratory capacity of A549 and H1975 cells (Figures S5H and S5I). The findings demonstrated that A549 cells with diminished RBM15 expression formed smaller tumors in nude mice and exhibited reduced growth rates (Figures 5A–5C). The IHC results indicated that RBM15 knockdown could reduce the expression of Ki67 (Figure 5D). These data suggest that the downregulation of RBM15 significantly inhibits cellular proliferation and migration in LUAD.

Then we investigated whether RBM15 could regulate the expression of DGUOK-AS1. The RT-qPCR results indicated that the expression levels of DGUOK-AS1 201 decreased upon suppression of RBM15, whereas overexpression of RBM15 led to a significant increase in DGUOK-AS1 expression levels (Figures 5E and 5F). However, it was observed that the expression levels of DGUOK-AS1 202 remained largely unchanged despite variations in RBM15 expression (Figure 5F). Thus, we hypothesized that m6A might influence the stability of DGUOK-AS1 201. The results showed a significant decrease in the stability of DGUOK-AS1 after RBM15 knockdown in A549 cells (Figure 5G), thereby suggesting that RBM15-induced m6A modifications have the potential to regulate the levels of DGUOK-AS1 in LUAD cells.

The results of subsequent rescue experiments indicated that overexpression of DGUOK-AS1 201 abrogated the suppressive effects of reduced RBM15 expression on cell proliferation and migration (Figures 5H and 5I). Restoration of DGUOK-AS1 201 may counteract EMT, which was inhibited by RBM15 downregulation, as shown by decreased E-cadherin and increased vimentin (Figure S5J). These findings suggest that RBM15 knockdown reduces the expression of DGUOK-AS1, thereby inhibiting cell proliferation and migration. We have confirmed that overexpression of DGUOK-AS1 can increase the expression of PRMT5 (Figure 3O), and overexpression of RBM15 leads to an increase in DGUOK-AS1 expression (Figure 5F). RBM15 overexpression inhibited miR-2467-5p expression (Figure 5J) and promoted PRMT5 expression (Figure 5K), while RBM15 knockdown promoted miR-2467-5p expression (Figure 5J) and inhibited PRMT5 expression (Figure 5K). We further speculated that RBM15 may affect cell growth through the DGUOK-AS1 201/miR-2467-5p/PRMT5 axis.

### HNRNPH1 binds DGUOK-AS1 in an m6A-dependent manner

**Figure 6:**
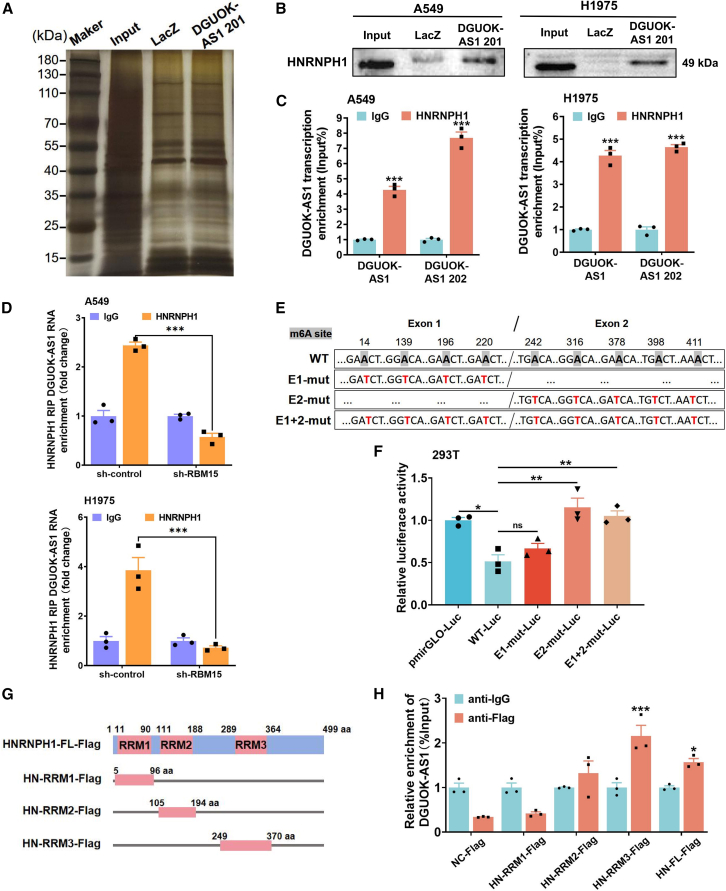
HNRNPH1 binds DGUOK-AS1 in an m6A-dependent manner (A) Silver staining of DGUOK-AS1 201 pull-down products. (B) RNA pull-down assay confirming the interaction between DGUOK-AS1 201 and HNRNPH1 in A549 and H1975 cells (*n* = 3). (C) The RIP-qPCR assay was employed to detect DGUOK-AS1 using either the HNRNPH1 antibody or IgG (*n* = 3, ∗∗∗*p* < 0.001). (D) Assessment of RIP assays detecting DGUOK-AS1 201 mRNA retrieved by a HNRNPH1 antibody or by IgG in A549 and H1975 cells after RBM15 knockdown (*n* = 3, ∗∗∗*p* < 0.001). (E) Schematic representation of WT or m6A sites mutated (E1-mut, E2-mut, and E1+2-mut) DGUOK-AS1 201 dual-luciferase reporter plasmids. (F) Luciferase activity of WT or mutated (E1-mut, E2-mut, and E1+2-mut) DGUOK-AS1 201 reporters in 293T cells with ectopically expressed HNRNPH1 (*n* = 3, ∗*p* < 0.05, ∗∗*p* < 0.01; ns, not significant). (G) Schematic of HNRNPH1 truncation constructs. (H) RIP-qPCR analysis for DGUOK-AS1 201 enrichment in A549 cells infected with lentivirus overexpressing 3×FLAG-tagged full-length or truncated HNRNPH1 proteins (*n* = 3, ∗*p* < 0.05, ∗∗∗*p* < 0.001). Results are expressed as mean ± SEM based on three independent trials. For (A–H), *n* represents the number of independent biological replicates, unless otherwise specified. Statistical analysis was performed using a one-way ANOVA (F) or two-way ANOVA (C, D, and H) followed by Tukey’s multiple comparison test.

Evidence indicates that LncRNAs perform various cellular functions through interactions with proteins or other cellular factors. RNA pull-down assays coupled with LC-MS (liquid chromatography/mass spectrometry) were conducted in A549 cells to identify proteins that directly interact with DGUOK-AS1. The mass spectrometry (MS) proteomics data have been deposited to the ProteomeXchange Consortium (https://proteomecentral.proteomexchange.org) via the iProX partner repository with the dataset identifier PXD075190.^38^^,^^39^ The SDS-PAGE silver staining analysis of the pull-down products showed the enrichment of several distinct bands ranging from 15 kDa to 130 kDa (Figure 6A). Twenty-four proteins with significant expression levels were identified (Table S4). Among these, 16 showed increased binding affinity, while 8 exhibited a reduced binding capacity (Table S4). MS analysis and western blot revealed that the DGUOK-AS1 201 probe group showed increased enrichment of the HNRNPH1 protein (Table S4; Figure 6B). The RIP-qPCR findings showed a higher abundance of DGUOK-AS1 in the precipitates from the HNRNPH1 antibody group compared to the IgG control group (Figure 6C). The results of anti-HNRNPH1 RIP-qPCR for DGUOK-AS1 in cells with or without RBM15 knockdown showed that RBM15 depletion significantly reduced the enrichment of DGUOK-AS1 in HNRNPH1 immunoprecipitates (Figure 6D), indicating that HNRNPH1 binding is largely dependent on prior m6A deposition by RBM15. Moreover, two truncation fragments were constructed based on the two exons comprising DGUOK-AS1 (Figures S6A and S6B). The “Exon 1 + 2” fragments of DGUOK-AS1 may predominantly mediate the interaction of DGUOK-AS1 with HNRNPH1 (Figure S6C). To test whether HNRNPH1 binding is m6A-dependent, we constructed pmirGLO luciferase reporters harboring WT DGUOK-AS1 or MUT in which the m6A motifs in exon 1 (E1-mut), exon 2 (E2-mut), or both (E1+2-mut) (Figure 6E). Expression of HNRNPH1 significantly reduced the luciferase activity of the WT reporter, indicating HNRNPH1-mediated degradation. The E2-mut reporter partially restored luciferase activity, while the E1+2-mut reporter showed a near-complete rescue (Figure 6F). These results confirm that HNRNPH1 recognizes and degrades DGUOK-AS1 in an m6A-dependent manner. Subsequently, the results of RIP assays utilizing 3×FLAG-tagged truncated and full-length fragments of the HNRNPH1 protein demonstrated that the RNA recognition motif 3 (RRM3, amino acids 249–370) and full-length fragments of HNRNPH1 bind to DGUOK-AS1 (Figures 6G and 6H; Figure S6D). The results indicated that HNRNPH1 binds DGUOK-AS1 in an m6A-dependent manner, an interaction predominantly mediated by its RRM3 domain.

### HNRNPH1 inhibits cell growth by modulating the stability of DGUOK-AS1

**Figure 7:**
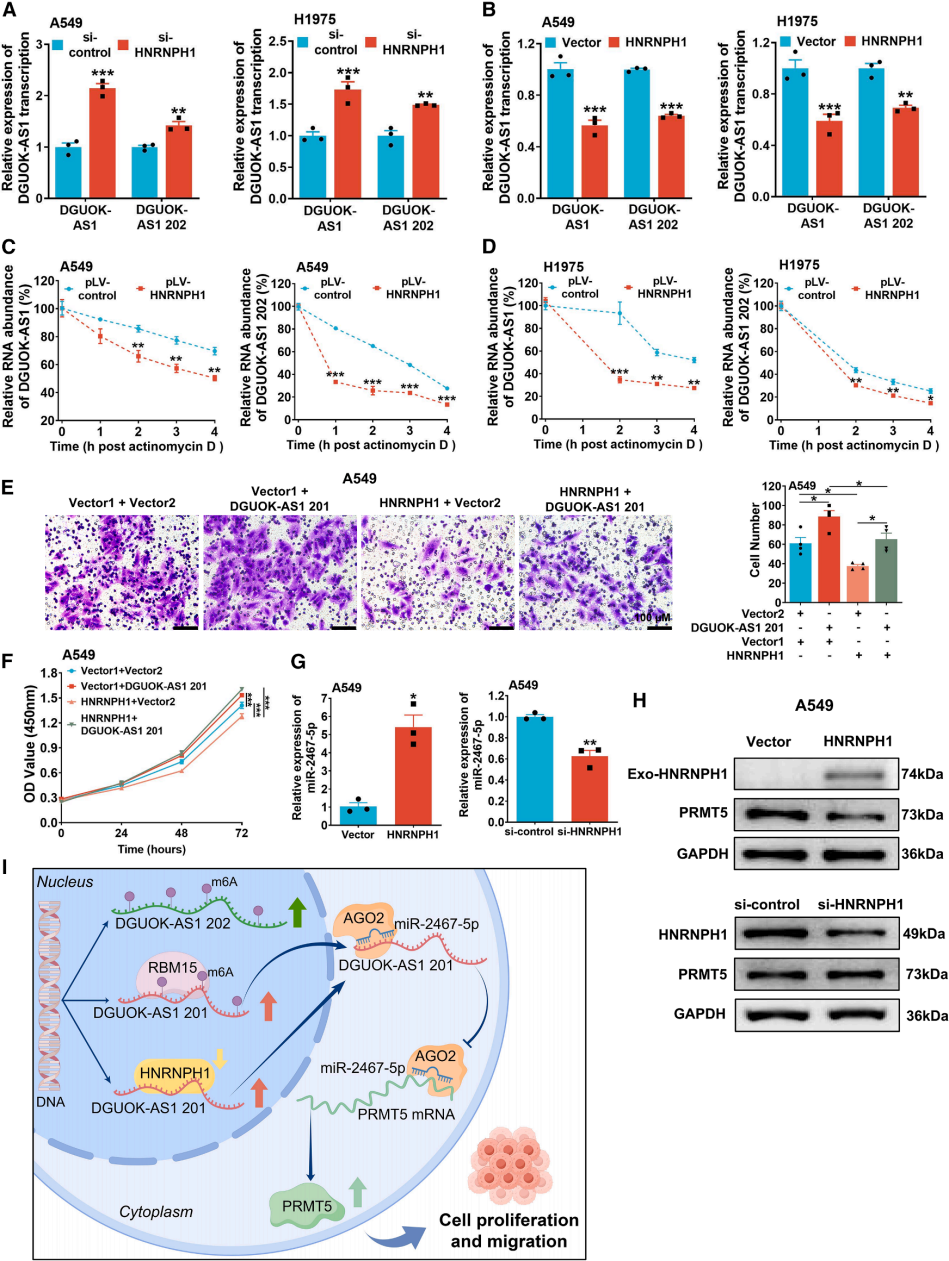
HNRNPH1 regulates the expression of DGUOK-AS1 (A and B) RT-qPCR assay measuring the expression of DGUOK-AS1 following knockdown or overexpression of HNRNPH1 (*n* = 3, ∗∗*p* < 0.01, ∗∗∗*p* < 0.001). (C and D) The RNA stability of DGUOK-AS1 in A549 and H1975 cells was assessed after HNRNPH1 overexpression (*n* = 3, ∗*p* < 0.05, ∗∗*p* < 0.01, ∗∗∗*p* < 0.001). (E) The migration ability was measured using Transwell assay when DGUOK-AS1 201 was overexpressed in HNRNPH1-overexpression cells (scale bars, 100 μm; *n* = 4, ∗*p* < 0.05). (F) CCK-8 assay was used to assess the proliferation of A549 cells co-transfected with HNRNPH1 and DGUOK-AS1 201 overexpression vectors (*n* = 3, ∗∗∗*p* < 0.001). (G) The expression of miR-2467-5p in A549 cells after overexpressing or knocking down HNRNPH1 by RT-qPCR analysis. (H) The protein levels of PRMT5 in A549 cells with overexpressed or knocked down HNRNPH1 (*n* = 3, ∗*p* < 0.05, ∗∗*p* < 0.01). (I) Molecular mechanism diagram of DGUOK-AS1 promoting cancer progression in LUAD. Each data point represents the mean ± SEM based on three independent trials. For (A–I), *n* represents the number of independent biological replicates, unless otherwise specified. Statistical analysis was performed using a paired *t* test (G), one-way ANOVA (E), or two-way ANOVA (A–D) and (F) followed by Tukey’s multiple comparison test.

To investigate the molecular mechanism by which HNRNPH1 regulates DGUOK-AS1, we altered the expression levels of HNRNPH1 in A549 and H1975 cells (Figures S7A–S7D). The RT-qPCR analysis showed that silencing HNRNPH1 resulted in a general increase in the expression levels of DGUOK-AS1 (Figure 7A). Furthermore, overexpression of HNRNPH1 led to decreased expression levels of DGUOK-AS1 (Figure 7B). Given that HNRNPH1 functions as an RNA-binding protein, we next evaluated the effect of HNRNPH1 on the stability of DGUOK-AS1 via actinomycin D RNA stability experiments. The overexpression of HNRNPH1 resulted in a reduction in both the half-life and expression level of DGUOK-AS1 in A549 and H1975 cells (Figures 7C and 7D), indicating that HNRNPH1 may play a role in modulating the stability of DGUOK-AS1.

HNRNPH1 shows low expression levels in LUAD from TCGA database and LUAD cells (Figures S7E–S7G). Consequently, we hypothesized that HNRNPH1 might affect cell growth via modulating the stability of DGUOK-AS1. The results of rescue experiments showed that DGUOK-AS1 overexpression significantly counteracted the inhibition of cell proliferation and migration in A549 cells, which was induced by the overexpression of HNRNPH1 (Figures 7E and 7F). In addition, restoration of DGUOK-AS1 201 may encourage EMT, which was suppressed by HNRNPH1 upregulation, as evidenced by reduced E-cadherin and increased vimentin (Figure S7H).

We have confirmed that silencing HNRNPH1 leads to an increase in DGUOK-AS1 expression (Figure 7A). We further speculated that HNRNPH1 may affect cell growth through the DGUOK-AS1 201/miR-2467-5p/PRMT5 axis. HNRNPH1 overexpression promoted miR-2467-5p expression (Figure 7G) and inhibited PRMT5 expression (Figure 7H), while silencing HNRNPH1 inhibited miR-2467-5p expression (Figure 7G) and promoted PRMT5 expression (Figure 7H). The findings suggest that HNRNPH1 silencing reduces DGUOK-AS1 degradation, and that this stabilization partially promotes cellular growth through the DGUOK-AS1 201/miR-2467-5p/PRMT5 axis.

## Discussion

DGUOK-AS1, located on chromosome 2p13.1, has been associated with the progression of multiple cancers.^10^^,^^11^^,^^13^ Through integrated TCGA data analysis and sequence alignment, this study identifies two DGUOK-AS1 transcript variants (designated DGUOK-AS1 201 and DGUOK-AS1 202) as potential regulatory factors in LUAD. LncRNAs circulating in serum exhibit remarkable stability due to their association with exosomes or protein complexes, which confer resistance to RNase degradation.^40^ Thus, LncRNAs can be considered as promising diagnostic and prognostic biomarkers for various cancers.^41^^,^^42^^,^^43^ DGUOK-AS1 was significantly associated with Tumor Node Metastasis (TNM) stage, while not correlated to gender, age, tumor size, histological cell type, lymph node metastasis, and differentiation,^13^ which aligns with and corroborates our findings from serum samples. Our recent findings demonstrate that DGUOK-AS1 expression is upregulated in the serum of NSCLC patients, suggesting its potential as a novel diagnostic biomarker for NSCLC (Figure 1G). Together, these consistent results across independent cohorts and sample types strengthen the potential of DGUOK-AS1 as a clinically relevant biomarker.

Notably, the majority of LncRNAs selectively encapsulated within exosomes originate from the cytoplasm of donor cells. Following exosomal delivery, these LncRNAs mainly localize to the cytoplasm of recipient cells, where they modulate signaling pathways or translational processes to exert their functions. Our investigation revealed that DGUOK-AS1 201 is distributed mostly in the cytoplasm. Furthermore, functional assays demonstrated that DGUOK-AS1 201, but not 202, plays a dominant role in promoting the proliferation and migration of LUAD cells. Consequently, we postulate that the stable persistence of DGUOK-AS1 201 in the serum of LUAD patients is likely dependent on its association with exosomes, which protects it from degradation. Studies have shown that LncRNAs typically act as ceRNAs in the cytoplasm.^41^^,^^44^ Our findings confirm that DGUOK-AS1 201 acts as a ceRNA to sponge miR-2467-5p to release PRMT5, which enhances cell proliferation and migration.

To explore the broader regulatory potential of DGUOK-AS1, we performed RNA-seq in A549 cells upon DGUOK-AS1 knockdown. Kyoto Encyclopedia of Genes and Genomes (KEGG) enrichment analysis revealed that downregulated genes were associated with lysosomal degradation, homologous recombination, and the Fanconi anemia pathway, while upregulated genes were enriched in ribosome biogenesis and stress-associated pathways including IL-17, TNF, and PI3K-Akt signaling (Figures S8A–S8C). Notably, “microRNAs in cancer” was among the top enriched terms for downregulated genes, consistent with our validated miR-2467-5p/PRMT5 axis and the established role of PRMT5 in DNA repair. These transcriptome-wide findings suggest that DGUOK-AS1 may exert broader homeostatic functions beyond the characterized axis, warranting future investigation.

m6A modification represents a core mechanism within the epitranscriptome for regulating LncRNAs stability. Critically, it can either promote degradation or enhance stabilization of target LncRNAs. Recent studies have highlighted the significant role of RBM15 in the methylation modification of mRNA in various cancers.^28^^,^^29^^,^^30^ However, the binding of RBM15 to LncRNAs and its consequent regulation of LncRNA m6A modification remain poorly characterized. RIP-qPCR and RNA pull-down assays confirmed the specific interaction between RBM15 and the DGUOK-AS1 201 transcript. Overexpression of RBM15 significantly enhanced m6A modification and concomitantly increased the stability of DGUOK-AS1 201. However, these analyses did not detect any m6A-mediated enhancement of DGUOK-AS1 202 expression. We propose that unknown mechanisms may specifically regulate m6A modification of the DGUOK-AS1 202 transcript.

Given that LncRNA performs its biological function mainly through interactions with RBPs, we further identified the captured DGUOK-AS1 201 binding proteins through LC-MS and found that HNRNPH1 might interact with DGUOK-AS1 201 and 202. HNRNPH1, which belongs to the HNRNPH1 (hnRNPs) family, plays a vital role in regulating alternative splicing,^31^^,^^43^^,^^44^ tissue-specific gene transcription, RNA transport, translation, and RNA stability,^32^^,^^33^ thereby influencing gene expression. Our findings demonstrate that the RRM3 domain of HNRNPH1 primarily mediates its binding interactions with both full-length of DGUOK-AS1 201 and 202. This study demonstrates that HNRNPH1 interacts with both transcript variants of DGUOK-AS1 and thereby accelerates their degradation rate. Furthermore, analysis of LUAD tumor samples from TCGA and LUAD cell lines revealed downregulation of HNRNPH1 expression (Figures S7E–S7G). Consequently, HNRNPH1 expression demonstrates an inverse correlation with DGUOK-AS1 transcript levels in LUAD cells.

A recent study indicates that m6A modification has been shown to regulate the alternative splicing of LncRNAs, such as LINC00475,^33^ Interestingly, our sequence analysis revealed that a distinct region (nt 300–325) within DGUOK-AS1 202 exhibits intron-like characteristics not present in the DGUOK-AS1 201 transcript (Figure S5A). Furthermore, our results demonstrate that DGUOK-AS1 202 is predominantly localized to the nucleus of LUAD cells (Figures 1I and 1J; Figures S1C and S1D). Therefore, we hypothesize that DGUOK-AS1 202 may undergo splicing to generate DGUOK-AS1 201, potentially in response to accelerated cellular proliferation cues.

However, the lack of extensive follow-up and clinical data precluded robust survival or multivariate analysis. Therefore, the potential of DGUOK-AS1 as a clinical biomarker requires future validation in a large, well-annotated, independent patient cohort with complete long-term follow-up data. Our preliminary ROC analysis serves as a foundation for these essential future studies. This study did not investigate whether m6A modification participates in the splicing of LncRNA DGUOK-AS1 transcripts, and the mechanism underlying m6A modification of DGUOK-AS1 202 currently lacks experimental validation. Moreover, fully elucidating the precise mechanistic coordination between RBM15 and HNRNPH1 is crucial.

In summary, we found that the LncRNA DGUOK-AS1 is highly expressed in LUAD cells, partially enhancing cell proliferation and migration through the miR-2467-5p/PRMT5 axis in the cytoplasm. Furthermore, RBM15 stabilizes DGUOK-AS1 201 in the nucleus, which promotes the m6A modification and expression of DGUOK-AS1. Additionally, the RRM3 domain of HNRNPH1 can physically interact with DGUOK-AS1 in an m6A-dependent manner, leading to its degradation. The downregulation of HNRNPH1 in LUAD cells results in the upregulation of DGUOK-AS1 expression. These findings provide the initial evidence for the existence of a DGUOK-AS1 201/miR-2467-5p/PRMT5 axis regulated by RBM15 and HNRNPH1 in LUAD cells (Figure 7I). The study elucidated the novel mechanisms of DGUOK-AS1 in both the cytoplasm and the nucleus, uncovering its role in cancer development and its potential as a biomarker for lung cancer.

### Limitations of the study

Several limitations of this study should be acknowledged. First, the clinical association of serum DGUOK-AS1 was evaluated in a single-center cohort of modest size, and the lack of extensive follow-up and clinical annotation precluded robust survival or multivariate analysis. Independent multi-center validation in a large, well-annotated patient cohort with complete long-term follow-up data is required to establish its clinical utility. Second, although our data demonstrate that HNRNPH1 binds DGUOK-AS1 in an m6A-dependent manner and that RBM15 knockdown reduces HNRNPH1 enrichment, the precise mechanistic relationship between RBM15 and HNRNPH1—such as whether they act sequentially on the same transcript or compete for overlapping binding sites—remains unresolved. Third, the functional differences between the DGUOK-AS1 201 and 202 isoforms have not been fully elucidated. While we hypothesize that DGUOK-AS1 202 may represent a processing intermediate of 201, the mechanism underlying m6A modification of the 202 transcript currently lacks experimental validation, and whether m6A participates in the splicing of DGUOK-AS1 transcripts remains unknown. Finally, the RNA-seq analysis provides a descriptive transcriptome-wide perspective; the functional significance of individual enriched pathways requires dedicated investigation in future studies.

## Resource availability

### Lead contact

Requests for further information and resources should be directed to and will be fulfilled by the lead contact, Hongfang Sun (sunhongfang@bzmc.edu.cn).

### Materials availability

This study did not generate new unique reagents.

### Data and code availability

• Raw RNA-sequencing data are deposited in the NCBI SRA database (SRA ID: PRJNA1492077) at https://www.ncbi.nlm.nih.gov/sra/PRJNA1492077. Mass spectrometry data are deposited to ProteomeXchange via iProX (ProteomeXchange ID: PXD075190) at https://www.iprox.cn/page/project.html?id=IPX0015969000. TCGA-LUAD data were obtained from The Cancer Genome Atlas via the GDC Data Portal (https://portal.gdc.cancer.gov/). Original western blot images are in the supplemental information (Data S1).
• This paper does not report original code.
• Any additional information required to reanalyze the data reported in this paper is available from the lead contact upon request.

## Acknowledgments

This work was supported by the 10.13039/501100001809National Natural Science Foundation of China (nos. 82002604, 32301062), the Development Plan of Youth Innovation Team in Colleges and Universities of Shandong Province (no. 2021KJ101), the 10.13039/501100007129Natural Science Foundation of Shandong (nos. ZR2020QH221, ZR2023MH223, and ZR2022LSW002), the Shandong Province Taishan Scholar Project (no. ts201712067), and the Scientific Research Foundation of 10.13039/501100007322Binzhou Medical University (no. 50012304278).

## Author contributions

H.S. and S.X. conceived the study, conducted data analysis, wrote the manuscript, and carried out the final editing of figures. M.Y. contributed to the design of artwork and manuscript writing. M.Y. and J.W. conducted the experiments and analyzed data. Y. Li and Y. Liang supplied experimental reagents and assisted with data analysis and manuscript writing. H.L., H.Z., Q.W., P.W., and Y.Y. provided assistance with the molecular biology experiments. G.S., J.F., and W.L. collected clinical samples. The manuscript underwent a thorough critical review by all authors.

## Declaration of interests

The authors declare no competing interests.

## STAR★Methods

### Key resources table

**Table:** 

REAGENT or RESOURCE	SOURCE	IDENTIFIER
**Antibodies**		

GAPDH polyclonal antibody	Bioworld	Cat# AP0063
PRMT5 Rabbit mAb	ABclonal	Cat# A19533; RRID: AB_2862652
RBM15 (E8Y8A) Rabbit Monoclonal Antibody	Cell Signaling Technology	Cat# 60386
HNRNPH1 Polyclonal antibody	Proteintech	Cat# 14774-1-AP; RRID: AB_2120339
Goat Anti-Rabbit IgG (H+L) HRP	Bioworld	Cat# ZJ2020-R
Goat Anti-Mouse IgG (H+L) HRP	Bioworld	Cat# BS12478
AGO2 Monoclonal antibody	Proteintech	Cat# 67934-1-Ig; RRID: AB_2918686
Mouse IgG	Proteintech	Cat# B900620
E-cadherin Polyclonal antibody	Proteintech	Cat# 20874-1-AP; RRID: AB_10697811
Vimentin Polyclonal antibody	Proteintech	Cat# 10366-1-AP; RRID: AB_2273020
[KO Validated] Ki67 Rabbit mAb	ABclonal	Cat# A20018; RRID: AB_3065688
Mouse anti DDDDK-Tag mAb	ABclonal	Cat# AE005; RRID: AB_2770401
N6-Methyladenosine (m6A) (D9D9W) Rabbit Monoclonal Antibody	Cell Signaling Technology	Cat# 56593
Normal Rabbit IgG	Cell Signaling Technology	Cat# 2729

**Bacterial and virus strains**		

DH5α Chemically Competent Cell	Tsingke Biotechnology	Cat# TSC-C14

**Chemicals, peptides, and recombinant proteins**		

RPMI-1640	Procell	Cat# PM150110
DMEM	Procell	Cat# PM150210
Fetal Bovine Serum	Vazyme	Cat# F101-01
Penicillin–streptomycin	Procell	Cat# PB180120
Actinomycin D	AAT Bioquest	Cat# 17505
ExFect Transfection Reagent	Vazyme	Cat# T101-01
Lipofectamine 2000	Invitrogen	Cat# 11668-019
RNA isolater Total RNA Extraction Reagent	Vazyme	Cat# R401-01
Transwell assay insert	BIOFIL	Cat# TCS003024
Crystal violet	Beyotime	Cat# C0121
4% paraformaldehyde	Biosharp	Cat# BL539A
BeyoMag™ RNA Clean Magnetic Beads	Beyotime	Cat# R0081
RIPA lysis buffer	Beyotime	Cat# P00138
PVDF	Millipore	Cat# IPVH00010

**Critical commercial assays**		

HiScript II Q RT SuperMix for qPCR(+gDNA wiper)	Vazyme	Cat# R223-01
ChamQ®SYBR qPCR Master Mix	Vazyme	Cat# Q311-02
Enhanced Cell Counting Kit 8	Elabscience	Cat# E-CK-A362
RNA Immunoprecipitation Kit	GENESEED	Cat# P0102
RNA pulldown Kit	BersinBio	Cat# Bes5102
Biotin RNA Labeling Kit (SP6/T7)	Beyotime	Cat# R7061S
Fast Silver Stain Kit	Beyotime	Cat# P0017S
Methylated RNA Immunoprecipitation(MeRIP) Kit	BersinBio	Cat# Bes5203-2
SABC Immunohistochemistry Kit	Boster	Cat# SA1022
Dual Luciferase Reporter Assay Kit	Vazyme	Cat# DL101-01

**Deposited data**		

mass spectrometry proteomics data	ProteomeXchange	ID: PXD075190
Raw RNA-sequencing data	NCBI SRA	ID: PRJNA1492077
TCGA-LUAD	NCI/GDC	TCGA-LUAD

**Experimental models: Cell lines**		

BEAS-2B	Shanghai Institute of Cell Biology	Cat# SCSP-5067
A549	Shanghai Institute of Cell Biology	Cat# SCSP-503
H1975	Shanghai Institute of Cell Biology	Cat# SCSP-597
PC-9	Procell	Cat# CL-0668; RRID:CVCL_B260
HEK293T	Shanghai Institute of Cell Biology	Cat# SCSP-502

**Experimental models: Organisms/strains**		

Female BALB/c nude mice	Hangzhou Ziyuan Experimental Animal Technology	Cat# S007-1

**Oligonucleotides**		

Primers	Tsingke Biotechnology	See Table S5
siRNA and microRNA mimics	Gene Pharma	See Table S6
FISH probes	Gene Pharma	See Table S7

**Recombinant DNA**		

pLV3-U6-MCS-shRNA-CopGFP-Puro	MiaoLing Biology	Cat# P29436
pLV3-U6-RBM15(human)-shRNA2-CopGFP-Puro	MiaoLing Biology	Cat# P48108
pCMV-MCS-3×FLAG-Neo	MiaoLing Biology	Cat# P8196P8196
pEnCMV-RBM15(human)-1-3×FLAG-SV40-Neo	MiaoLing Biology	Cat# P24503
pCMV-PRMT5(human)-3×FLAG-Neo	MiaoLing Biology	Cat# P61983
pCMV-mCherry-2×Linker-MCS-Neo	MiaoLing Biology	Cat# P41171
pCMV-mCherry-HNRNPH1(human)-6×His-Neo	MiaoLing Biology	Cat# P55662

**Software and algorithms**		

GraphPad	Prism	https://www.graphpad.com
ImageJ	Open-source software	https://doi.org/10.1038/nmeth.2089
GEPIA2	Open-source software	http://gepia2.cancer-pku.cn/#analysis
MEME Suite 5.5.9	Open-source software	https://meme-suite.org/meme/
miRDB	Open-source software	https://mirdb.org/
TargetScanHuman 8.0	Open-source software	https://www.targetscan.org/vert_80/
miRWalk	Open-source software	http://mirwalk.umm.uni-heidelberg.de/
SRAMP	Open-source software	https://www.cuilab.cn/m6asiteapp/old
RNAseq analysis	BGI Dr.Tom	https://biosys.bgi.com/#/report/project/projectList

### Experimental model and study participant details

#### Human subjects

Serum samples from 43 pairs of healthy individuals and lung cancer patients were gathered from the Yantai Affiliated Hospital of Binzhou Medical University between September 2022 and March 2024. Written informed consent was obtained from all subjects prior to sample collection. All subjects were of Han Chinese ethnicity. The cohort included 58 male and 28 female subjects, with an age range of 25-79 years. Sex was not found to be a significant confounding variable in the analyses performed. The present study was approved by the Medical Ethics Committee of Binzhou Medical University (no. 2021-102). The detailed patient information can be found in Table S8.

#### Cell lines

Human embryonic kidney 293T (HEK-293T) cells, human normal epithelial lung cells BEAS-2B, human lung cancer cells A549, H1975 were sourced from the Shanghai Institute of Cell Biology, China. Human lung adenocarcinoma cells PC-9 were obtained from Wuhan Procell, China. All cell lines were authenticated by short tandem repeat (STR) profiling. All cell lines were routinely tested for mycoplasma contamination and confirmed negative. A549, H1975, and PC-9 cells were cultured in RPMI-1640 medium supplemented with 10% fetal bovine serum and 1% penicillin - streptomycin. BEAS-2B and HEK293T cells were maintained in DMEM containing 10% fetal bovine serum and 1% penicillin - streptomycin. All cells were incubated at 37°C in a humidified atmosphere with 5% CO_2_.

#### Animals

Female BALB/c nude mice (4-6 weeks old, Hangzhou Ziyuan Experimental Animal Technology) were obtained for tumorigenesis research, following approved guidelines and protocols from the Animal Experimentation Ethics Committee of Binzhou Medical University (no. 2022-378). Mice were housed under specific pathogen-free (SPF) conditions at 22–24°C and provided with sterilized food and water *ad libitum*.

### Method details

#### Plasmid constructions and cell transfection

For the overexpression experiments, a full-length human DGUOK-AS1 201 or 202 sequence were amplified from HEK293T cells and subcloned into the pcDNA3.1 (-) vector. The overexpression plasmid of RBM15 and HNRNPH1 were synthesized from Miaoling Plasmid Platform. For knockdown of DGUOK-AS1, PRMT5 and HNRNPH1, specific small interfering RNAs (siRNAs) and miR-2467-5p mimic were purchased from Gene Pharma (Gene Pharma, Shanghai). The DGUOK-AS1 201/202 overexpression lentiviral plasmid and shRNA lentiviral plasmid were construction into the plv-FUGW vector. The RBM15 shRNA lentiviral plasmid and HNRNPH1 overexpression lentiviral plasmid were synthesized from Miaoling Plasmid Platform. Cell transfection was performed using the Lipofectamine 2000 transfection reagent (11668-019, Invitrogen) or ExFect Transfection Reagent (T101-01, Vazyme) following the manufacturer’s instructions.

#### Quantitative real-time PCR (qRT-PCR)

The total RNA Extraction Reagent (R401-01, Vazyme) was used to isolate RNAs from cells, which were then reverse transcribed into first strand cDNA using HiScript II Q RT SuperMix (R223-01, Vazyme). Additionally, qRT-PCR was conducted with a ChamQ®SYBR qPCR Master Mix (Q311-02, Vazyme) on a StepOnePlus Real-time system (ThermoFisher).

#### Western blotting

Total protein extraction was conducted as previously reported. Total proteins were extracted from transfected cells using RIPA lysis buffer (P0013B, Beyotime). Following this, the cell lysate was centrifuged at 12000 rpm for 15 mins at 4°C. The supernatant was collected, boiled with a 5×SDS loading buffer, and the proteins were separated through SDS-polyacrylamide gel electrophoresis before being transferred to polyvinylidene fluoride membranes (IPVH00010, Millipore). Membranes were blocked using a solution of 5% nonfat dry milk and incubated with primary antibodies PRMT5 (1:800, A19533, ABclonal), RBM15 (1:1000, 60386, CST), HNRNPH1 (1:1000, 14774-1-AP, Proteintech), E-cadherin (1:1000, 20874-1-AP, Proteintech), Vimentin (1:1000, 10366-1-AP, Proteintech) and GAPDH (1:6000, AP0063, Bioworld), followed by reaction with HRP-conjugated secondary antibodies (1:4000, Proteintech). Finally, the protein bands were revealed through the use of an enhanced chemiluminescence reagent (P0018S, Beyotime).

#### Cell proliferation assay (CCK-8)

Cell proliferation was measured using an enhanced cell counting kit 8 (E-CK-A362, Elabscience). Briefly, each well of a 96-well plate was populated with 2000 cells. After 24, 48, and 72 hours, each well was treated with 10 μL of CCK-8 solution, and the cells were incubated at 37°C for 2 hours. The absorbance at 450 nm for each well was finally measured with a microplate reader.

#### Transwell assay

Transwell migration assay was conducted using Transwell chambers (TCS003024, BIOFIL). Briefly, the upper chamber was seeded with 20,000 transfected cells, and the lower chamber was supplied with RPMI-1640 medium enriched with 30% FBS. After 24 hours of incubation, the chamber was fixed using a 4% paraformaldehyde solution and stained with crystal violet. Four randomly selected fields were counted under a microscope to determine the number of migratory cells (DM6000B, Leica).

#### Colony formation assay

The transfected cells were counted and plated with 500 cells into 6-well plates. And cells were cultured at 37°C with 5% CO_2_ for 8 days to allow colony formation. The cell colonies were treated with 4% paraformaldehyde for fixation and stained using crystal violet. After staining, photographs were taken and the colonies were counted for statistical analysis.

#### Wound healing assay

After the transfected cells cultured to 100% confluence in 6-well plates, a straight linear scratch wound was created in the cell monolayer. At 0, 24, and 48 hours, the distance of wound healing was measured, and the wound areas were photographed using a microscope (DM6000B, Leica). ImageJ software was used to measure the percentage of wound closure, indicating the cells' migration ability.

#### Fluorescent *in situ* hybridization (FISH)

The manipulations closely paralleled those previously described.^45^ Following fixation cells by 4% paraformaldehyde solution, the cells were exposed to a cell permeabilization agent for 15 mins, then incubated with a blocking solution at 37°C for 30 mins. Hybridization between fluorescently labeled RNA probes and cells was carried out overnight at 37°C. Subsequently, DAPI was used to stain cell nuclei, followed by mounting with an anti-fade medium, observations were made under a fluorescence microscope.

#### Luciferase reporter assay

Luciferase levels were analyzed using the Dual Luciferase Reporter Assay Kit (DL101-01, Vazyme). Cells were transfected with vectors that expressed luciferase or with control vectors. After 48 hours, add 100μL luciferase substrate equilibrated and 20μL cell lysis supernatant into the microplate well, quickly mix and immediately test the firefly luciferase reporter activity in the fluorescence tester (INFINITE M PLEX, TECAN). Then add 100μL freshly prepared Renilla substrate working solution, and immediately detect Renilla luciferase reporter gene activity in a fluorescence detector.

#### RNA immunoprecipitation (RIP)-qPCR

About 2×10^7^ cells were collected for RIP assays by using the PureBinding®RNA Immunoprecipitation Kit (P0102, Geneseed) in accordance with the manufacturer’s instructions. The amount of antibody used for each experiment was 5μg. Rotate them and antibody overnight at 4°C for thorough mixing, then separate and collect RNAs into RNase-free 1.5mL Eppendorf tubes. The obtained RNAs are reverse-transcribed in equal volumes and analyzed using qPCR.

#### Methylated RNA immunoprecipitation (MeRIP)

Total RNA was extracted from 2 × 10^7^ cells using the Methylated RNA Immunoprecipitation Kit (Bes5203-2, BersinBio) according to the manufacturer’s instructions. The purified RNA was fragmented to approximately 300 nucleotides by incubation in fragmentation buffer at 94°C for 2 min, and the reaction was terminated by adding EDTA followed by cooling on ice. The fragmented RNA was divided equally into two portions and incubated at 4°C for 4 h with either anti-m6A antibody (4 μg, 56593, CST) or control IgG antibody (4 μg, 2729, CST) under gentle rotation. Protein A/G magnetic beads were then added to capture the antibody-RNA complexes. After extensive washing, the enriched m6A-modified RNAs were eluted and analyzed by qPCR or PCR to assess the m6A levels on the lncRNA of interest.

#### RNA pull-down and LC-MS

Biotinylated full-length probes of DGUOK-AS1 201 and 202 were generated using *in vitro* transcription following the instructions of the Biotin RNA Labeling Kit (R7061S, Beyotime), and the pull-down assay was conducted using the RNA Pull-Down Kit (Bes5102, BersinBio). According to the mass ratio of 1 μg/1000 nt, the respective quantities of biotinylated target RNA probes and LacZ probes were used. Incubate streptavidin magnetic beads with the probes at 25°C for 30mins to form the probe-bead complexes. Mix the probe-bead complexes with 2×10^7^ cell extracts and rotate the mixtures at 25°C for 2 hours to capture proteins. Subsequently, the investigation of specific RNA-binding proteins interacting with DGUOK-AS1 201 and 202 was conducted using mass spectrometry or Western blotting. RNA pull-down products and LacZ pull-down products were sent to Shanghai Luming Biotechnology Co., Ltd. (Shanghai, China) for LC-MS. The eluted proteins were digested with trypsin and analyzed by LC-MS/MS on a Q Exactive HF mass spectrometer. Raw data were searched against the UniProt human database using MaxQuant with a 1% FDR at both peptide and protein levels.

#### RNA stability assay

The cells were placed in 6-well plates overnight and then subjected to 5 μg/mL actinomycin D (17505, AAT Bioquest) treatment for 0, 1, 2, 3, and 4 hours. Total RNA was isolated using total RNA Extraction Reagent and quantifed by qRT-PCR. Group expression of the mRNA at the indicated times was calculated and normalized by GAPDH.

#### Immunohistochemistry

Immunohistochemistry was performed as described previously. Xenograft tumors were fixed with 4% paraformaldehyde and embedded in paraffin. Then they were cut into sections of 5 μm. Following the instructions of the SABC Immunohistochemistry Kit (SA1022, Boster), antigen retrieval was performed using sodium citrate, and then endogenous peroxidase was blocked. The sections were incubated with specific Ki67 antibody (1:500, A20018, ABclonal) at 37°C for 2 hours. After incubation with secondary antibody at 37°C for 30 mins, DAB was used for detection. The sections were observed under the Leica Application Suite (DM6000B, Leica).

### Quantification and statistical analysis

All the experiments were repeated no less than three times, and GraphPad Prism (version 8.0.2) was utilized for statistical analysis. Data are shown as means ± SEM. Differences between two groups were analyzed using a paired Student’s *t* test. For comparisons among multiple groups, one-way or two-way ANOVA followed by Tukey’s post-hoc test was used. A *p*-value of less than 0.05 was considered statistically significant. Symbols ∗, ∗∗, and ∗∗∗ represented *p*-values of less than 0.05, 0.01, and 0.001, respectively. The exact value of *n*, what *n* represents, and the specific statistical test used for each experiment are reported in the respective figure legends.
