# Supplementary material for: m6A-dependent regulation of DGUOK-AS1 by RBM15 and HNRNPH1 in lung adenocarcinoma
Source: iScience. 2026 Aug 7;29(8):117078. doi: 10.1016/j.isci.2026.117078 (PMC13476473; doi:10.1016/j.isci.2026.117078)

iScience, Volume *29*

Supplemental information

m6A-dependent regulation of DGUOK-AS1 by RBM15 and HNRNPH1 in lung adenocarcinoma

Menghao Yang, Jiaen Wu, Youjie Li, Hui Li, Hongrong Zhang, Yan Liang, Qin Wang, Pingyu Wang, Yuan Yu, Guangbin Sun, Jiankai Feng, Wenwen Liu, Shuyang Xie, and Hongfang Sun

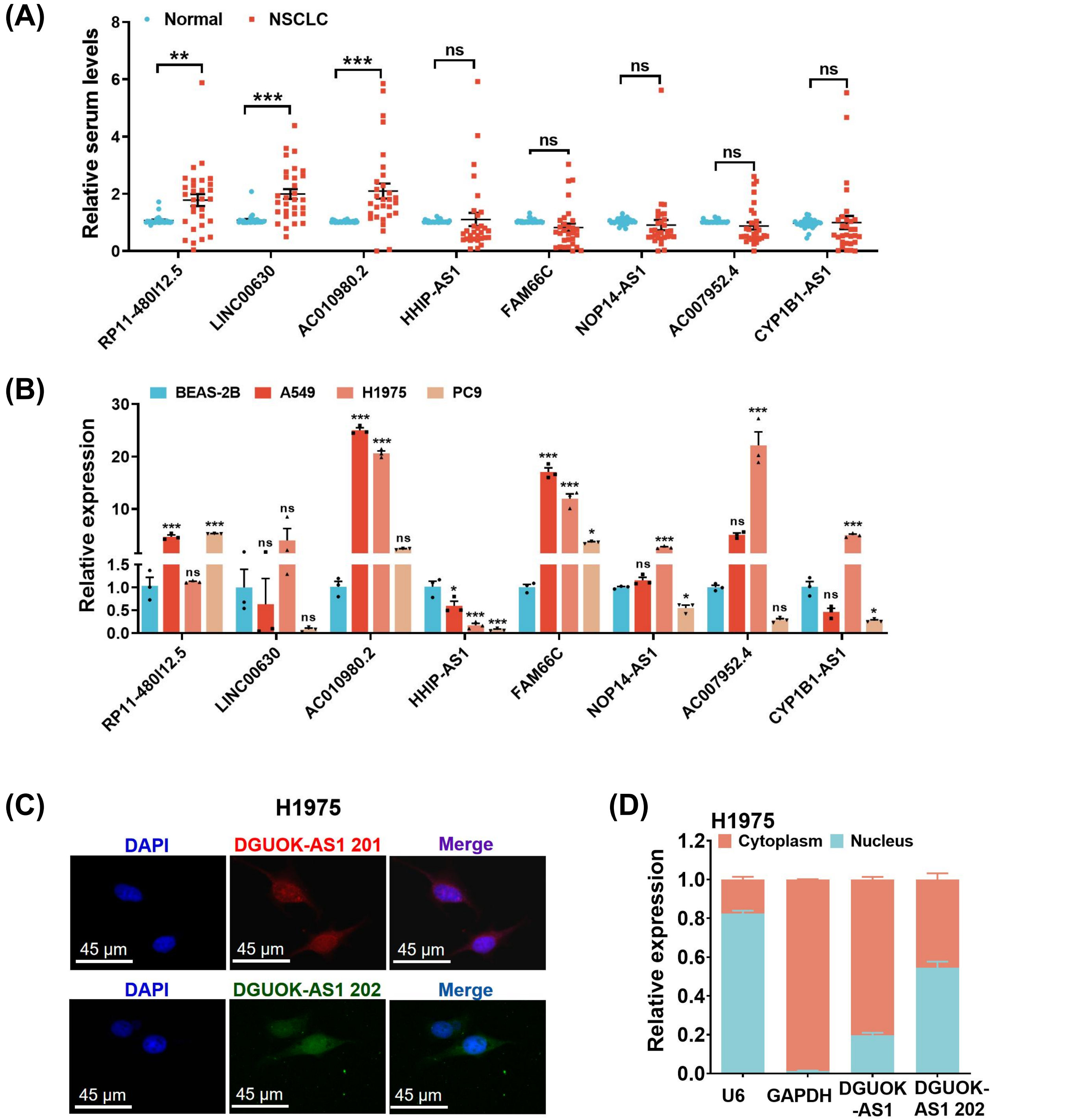

Figure S1 The expression levels of eight candidate lncRNAs and the subcellular localization of DGUOK-AS1. (A) 8 candidate lncRNAs expression in serum from 30 pairs of healthy individuals and NSCLC patients. (***p* < 0.01, ****p* < 0.001; ns, not significant). (B) The expression of the 8 candidate lncRNAs in BEAS-2B, A549, H1975 and PC-9 cells. (C) FISH analysis of DGUOK-AS1 (red) and DGUOK-AS1 202 (green) in H1975 cells. (D) DGUOK-AS1 201/202 expression in the cytoplasm and nucleus of H1975 cells. (scale bars, 45 μm). Data in all graphs are shown as mean ± SEM based on three independent trials. Statistical analysis was performed using a two-way ANOVA (A) or one-way ANOVA (B), followed by Tukey's multiple comparison test.

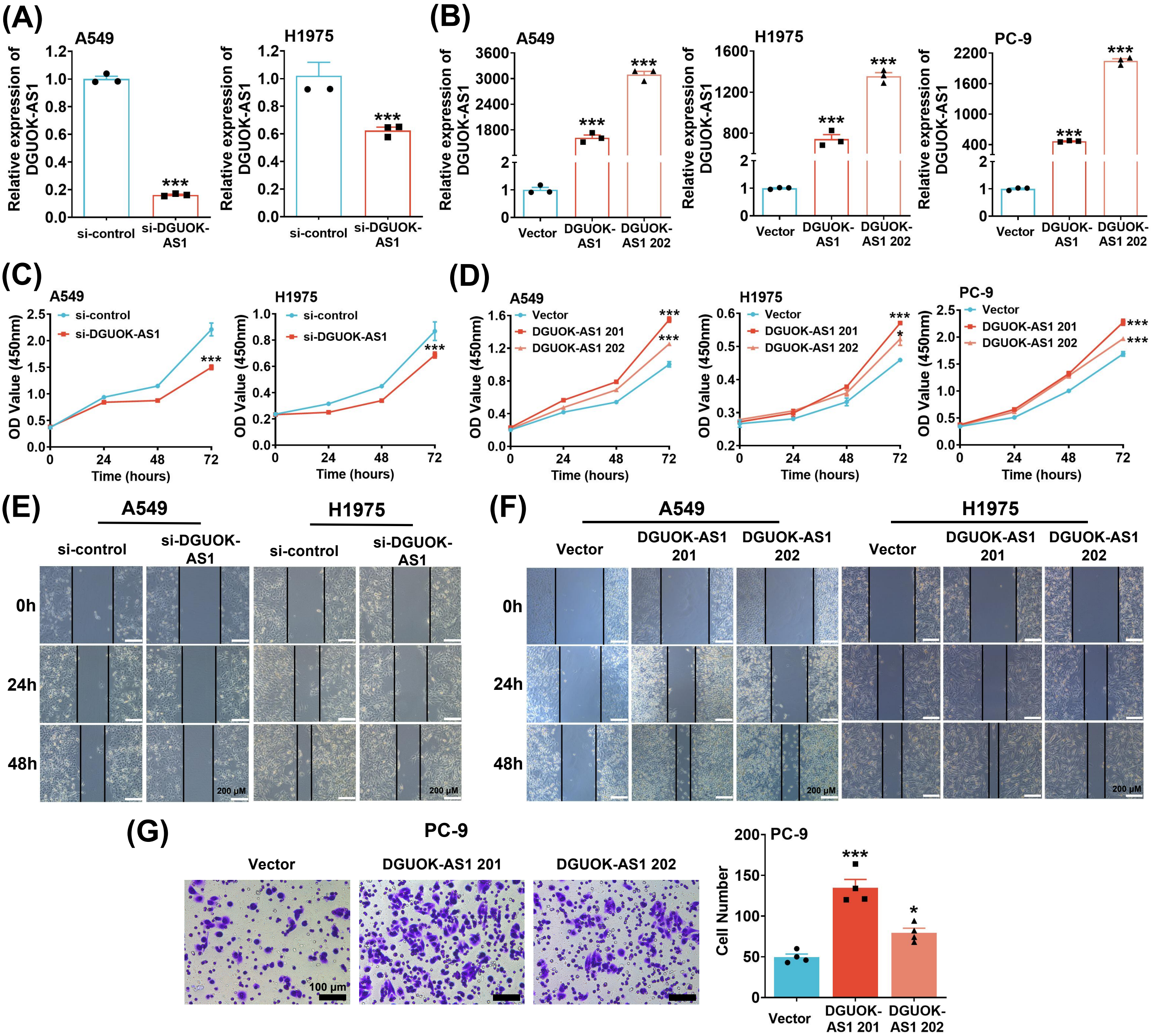

Figure S2 DGUOK-AS1 promotes proliferation and migration of LUAD cells. (A, B) The expression of DGUOK-AS1 in A549, H1975 and PC-9 cells after knocking down or overexpressing DGUOK-AS1 by qRT-PCR analysis. (***p < 0.001). (C) CCK-8 assay was performed to measure the cell proliferation of A549 and H1975 cells transfected with DGUOK-AS1 siRNA or scrambled controls. (***p < 0.001). (D) Cell proliferation analysis of A549, H1975 and PC-9 cells transfected with DGUOK-AS1 201, DGUOK-AS1 202 overexpression vector or control vector. (*p < 0.05, ***p < 0.001). (E, F) Wound healing migration assay of A549 and H1975 cells with DGUOK-AS1 knocked down or overexpressed. (scale bars, 200 μm). (G) Transwell were used to assess the migration of PC-9 cells transfected with DGUOK-AS1 201 or 202 overexpression vector. (*p < 0.05, ***p < 0.001). All data are presented as the mean ± SEM based on three independent trials. Statistical analysis was performed using a paired test (A), one-way ANOVA (B, G) or two-way ANOVA (C, D), followed by Tukey's multiple comparison test.

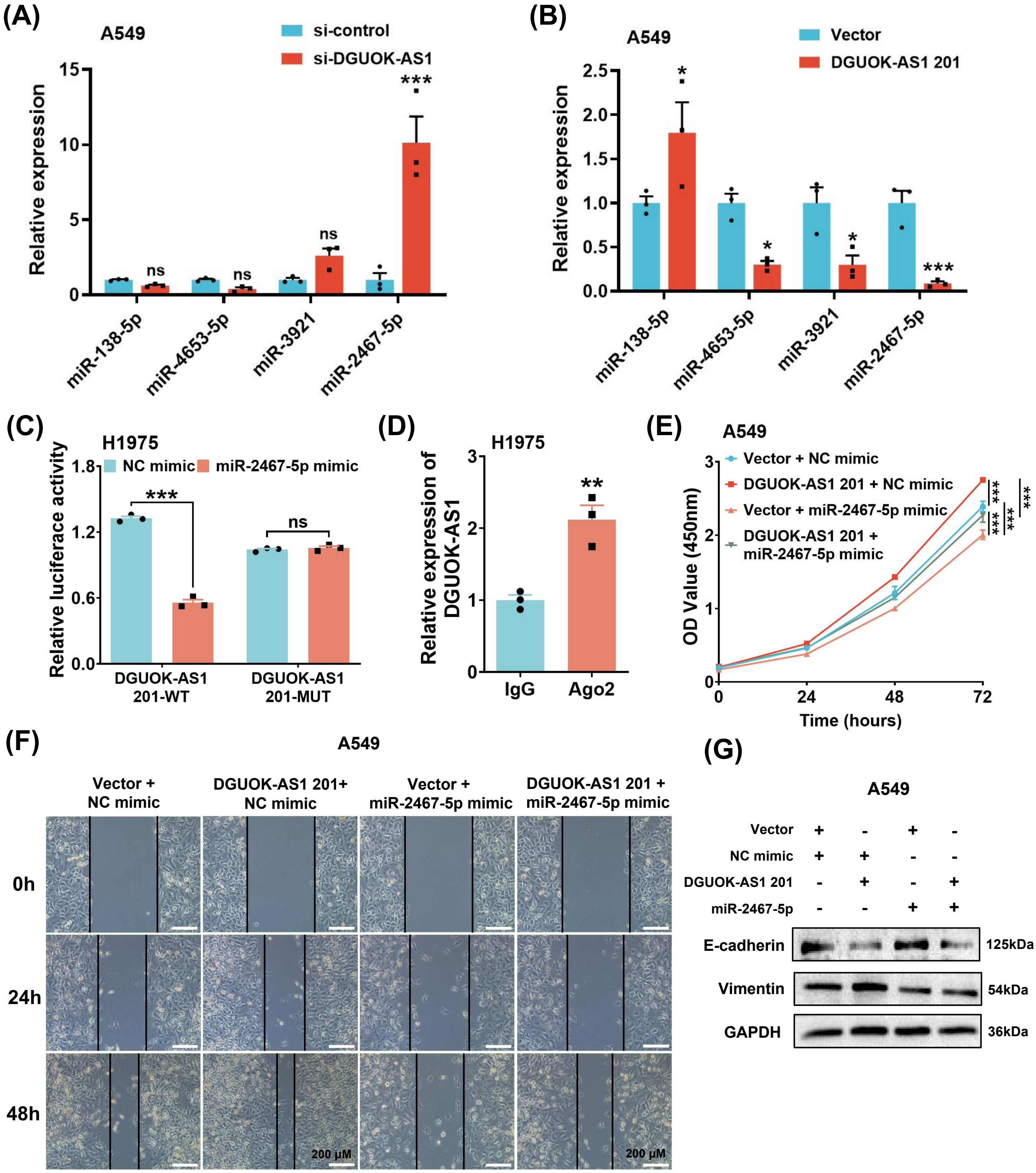

Figure S3 DGUOK-AS1 201 functions as the ceRNA of miR-2467-5p to promote cell proliferation and migration. (A, B) The expression of candidate miRNAs in A549 cells after knocking down or overexpressing DGUOK-AS1 201 by qRT-PCR analysis. (*p < 0.05, ***p < 0.001; ns, not significant). (C) Dual luciferase assay in H1975 cells co-transfected with WT or MUT DGUOK-AS1 201 luciferase vectors and miR-2467-5p mimics. (***p < 0.001; ns, not significant). (D) RIP assay of the enrichment of DGUOK-AS1 on Ago2 relative to IgG in H1975 cells. (**p < 0.01). (E) The proliferation of A549 cells with DGUOK-AS1 201 overexpression with or without overexpressing miR-2467-5p. (***p < 0.001). (F) Wound healing migration assay of A549 cells co-transfected with DGUOK-AS1 201 overexpression vector and miR-2467-5p mimic. (scale bars, 200 μm). (G) The expression of E-cadherin and Vimentin in A549 cells with DGUOK-AS1 201 overexpression vector and miR-2467-5p mimic treatment. Data are presented as the mean ± SEM of three independent experiments. Statistical analysis was performed using a paired test (D) or two-way ANOVA (A, B, C, E), followed by Tukey's multiple comparison test.

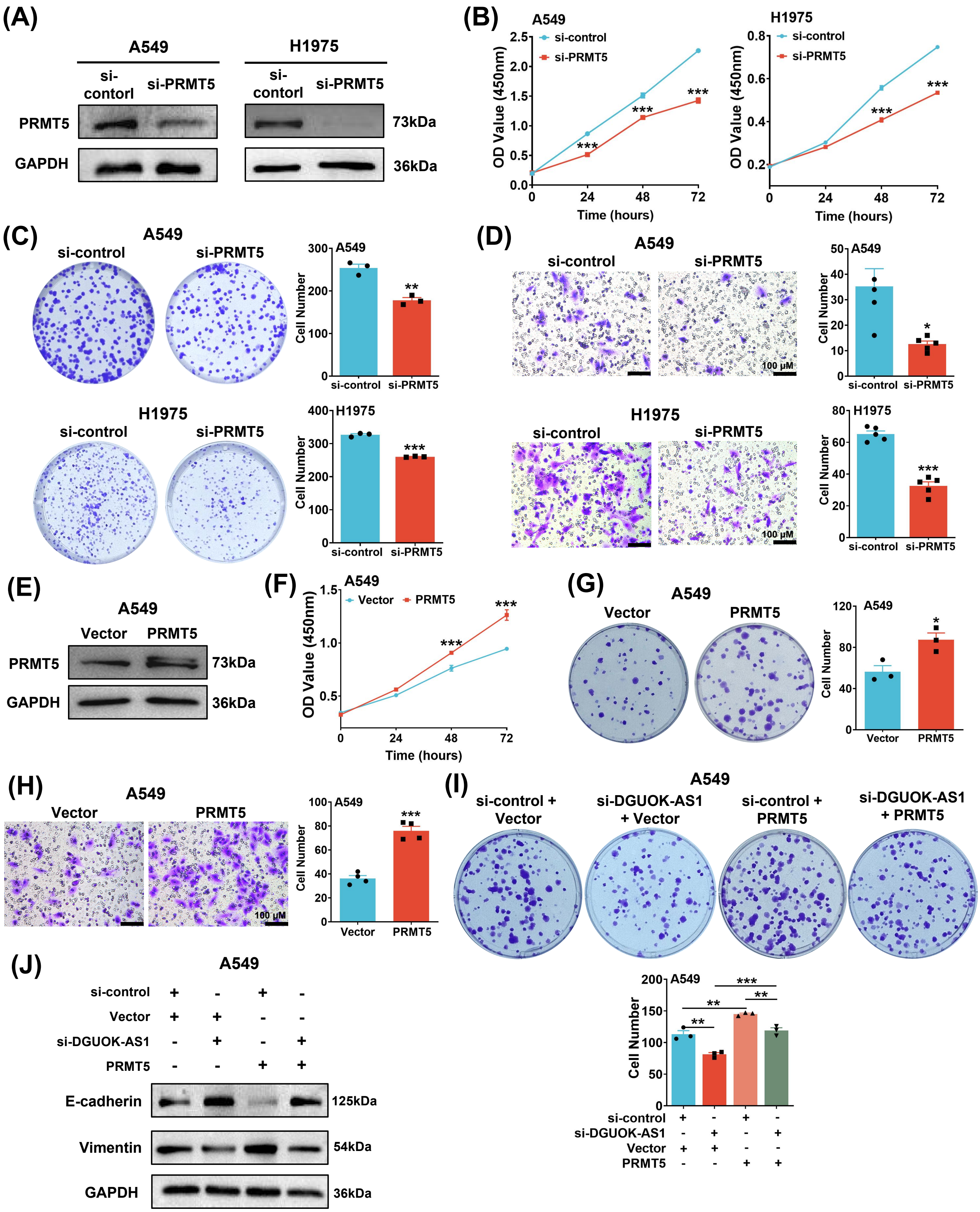

Figure S4 Knocking down PRMT5 inhibits LUAD cells proliferation and migration. (A) The protein expression of PRMT5 was inhibited in A549 and H1975 cells. (B-D) Cell proliferation and migration analysis of A549 and H1975 cells following PRMT5 knockdown. (scale bars, 100 μm; $*p < 0.05$, $**p < 0.01$, $***p < 0.001$). (E) The PRMT5 expression in A549 cells transfected with PRMT5 overexpression vector. (F-H) CCK-8, colony formation and transwell assays were performed to measure the cell proliferation and migration of A549 cells overexpressing PRMT5. (scale bars, 100 μm; $*p < 0.05$, $***p < 0.001$). (I) The clonogenicity was measured using colony formation assay when PRMT5 was overexpressed in DGUOK-AS1-silenced cells. ($**p < 0.01$, $***p < 0.001$). (J) Expression levels of E-cadherin and Vimentin in A549 cells co-transfected with PRMT5 overexpress vector and DGUOK-AS1 siRNA. All data are presented as the mean ± SEM of three independent experiments. Statistical analysis was performed using a paired test (C, D, G, H), one-way ANOVA (I) or two-way ANOVA (B, F), followed by Tukey's multiple comparison test.

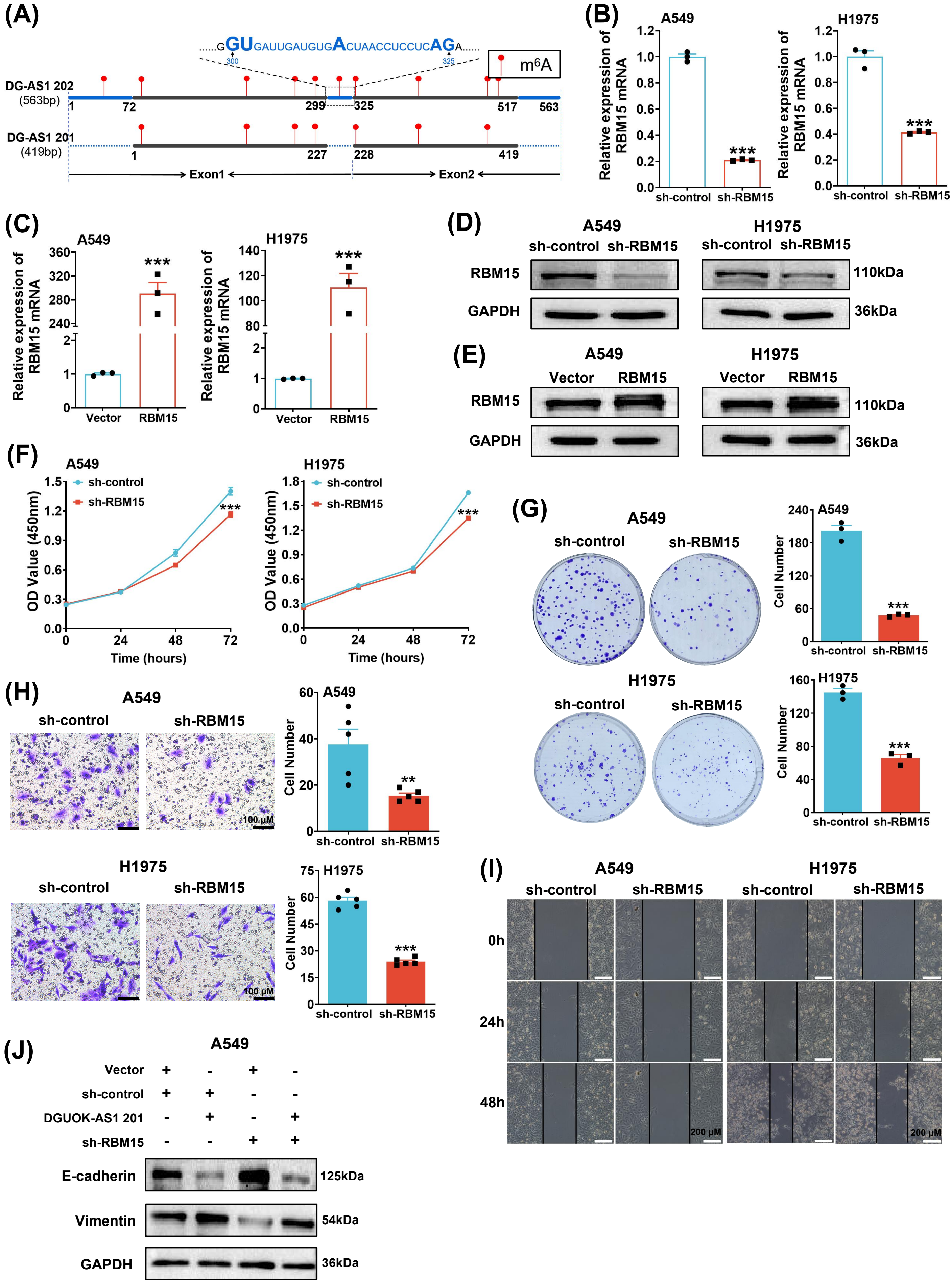

Figure S5 RBM15 knockdown suppresses cell proliferation and migration. (A) Diagram of the m6A sites on DGUOK-AS1 201/202 and the nucleotide sequence of the region spanning 300-325bp on DGUOK-AS1 202. (B-E) The mRNA and protein levels of RBM15 were identified following its knockdown or overexpression using qRT-PCR and western blotting assay. (****p* < 0.001). CCK-8 (F) and colony formation assay (G) were used to evaluate the proliferation of A549 and H1975 cells following with knocked down RBM15. (****p* < 0.001). (H, I) Transwell and wound healing migration assay in RBM15 knockdown A549 and H1975 cells. (Black scale bar, 100 μm; white scale bar, 200 μm; ***p* < 0.01, ****p* < 0.001). (J) Expression levels of E-cadherin and Vimentin when DGUOK-AS1 201 overexpressed in RBM15-silenced cells. All data are presented as the mean ± SEM of three independent experiments. Statistical analysis was performed using a paired test (B, C, G, H) or two-way ANOVA (F), followed by Tukey's multiple comparison test.

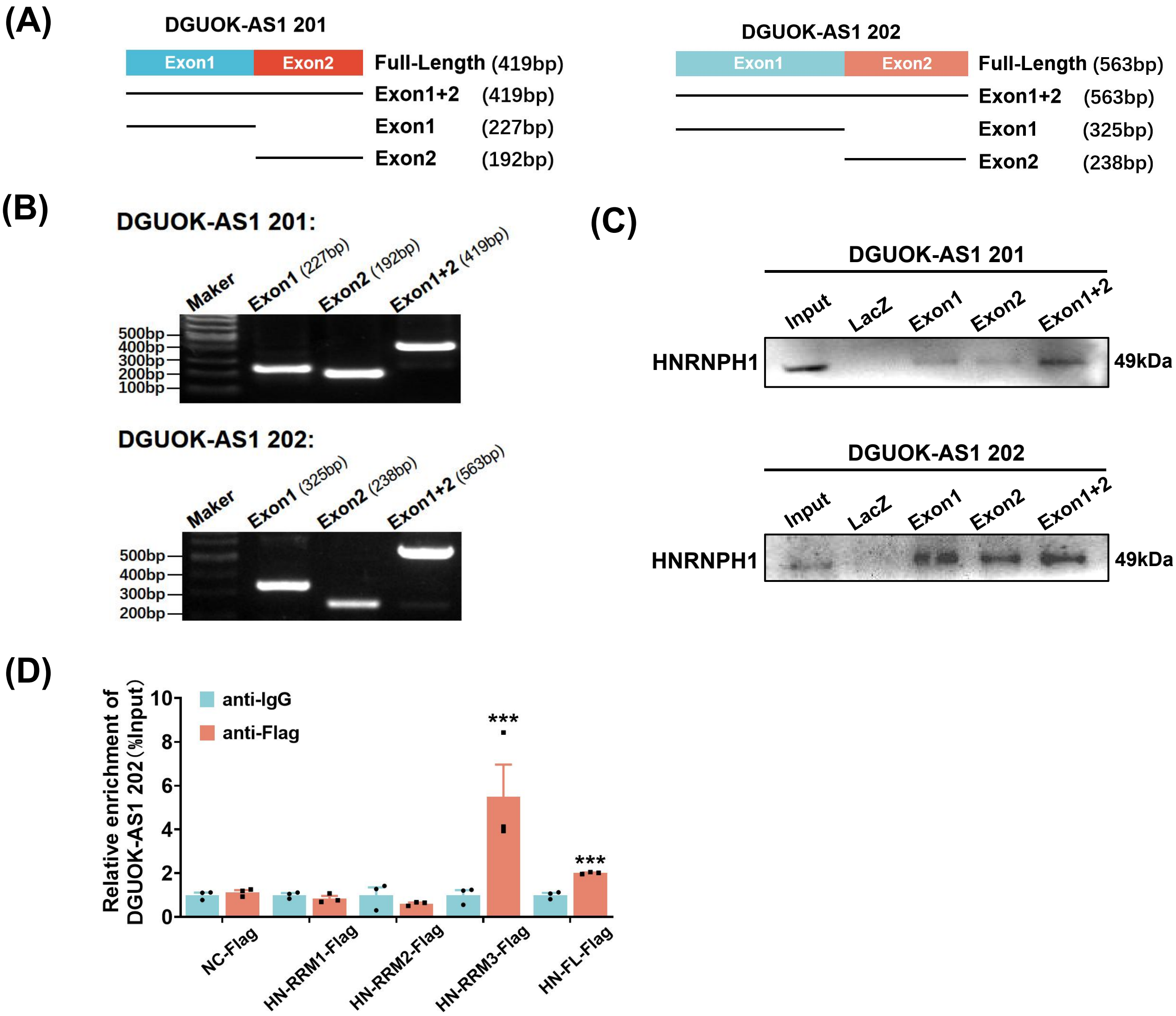

Figure S6 Identification of the interaction between DGUOK-AS1 and HNRNPH1. (A, B) Schematic of truncated DGUOK-AS1 201/202 RNA forms and agarose gel image of DGUOK-AS1 201/202 full-length and truncations. (C) Western blotting evaluated HNRNPH1 levels enriched by LacZ or DGUOK-AS1 201/202 truncated probes in A549 cells. (***p* < 0.01, ****p* < 0.001). (D) RIP-qPCR analysis for DGUOK-AS1 201 enrichment in A549 cells infected with lentivirus overexpressing 3×FLAG-tagged full-length or truncated HNRNPH1 proteins. (n = 3, ****p* < 0.001). Statistical analysis was performed using a two-way ANOVA (D), followed by Tukey's multiple comparison test.

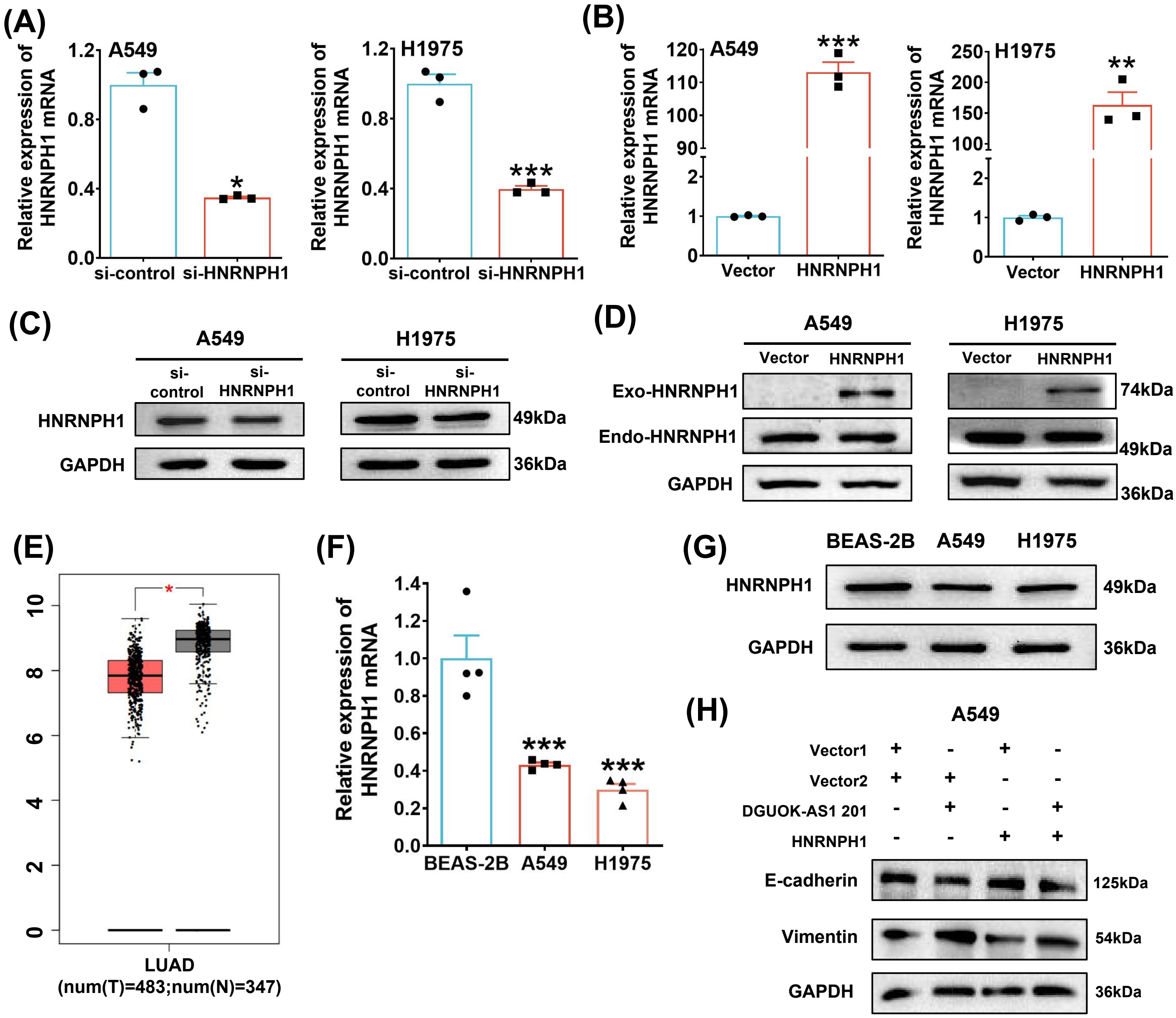

Figure S7 The expression of HNRNPH1 in LUAD. The qRT-PCR (A, B) and western blotting (C, D) were used to determine the expression levels of HNRNPH1 after its knockdown or overexpression. ($^{*}p < 0.05$, $^{**}p < 0.01$, $^{***}p < 0.001$). (E) The expression of HNRNPH1 in TCGA-LUAD database on the GEPIA online tool. The mRNA (F) and protein (G) expression levels of HNRNPH1 in BEAS-2B, A549 and H1975 cells. ($^{***}p < 0.001$). (H) E-cadherin and Vimentin expression in A549 cells co-transfected with DGUOK-AS1 201 overexpression vector and HNRNPH1 vector. Data are reported as mean ± SEM from three independent experimental runs. Statistical analysis was performed using a paired test (A, B), one-way ANOVA (F), followed by Tukey's multiple comparison test.

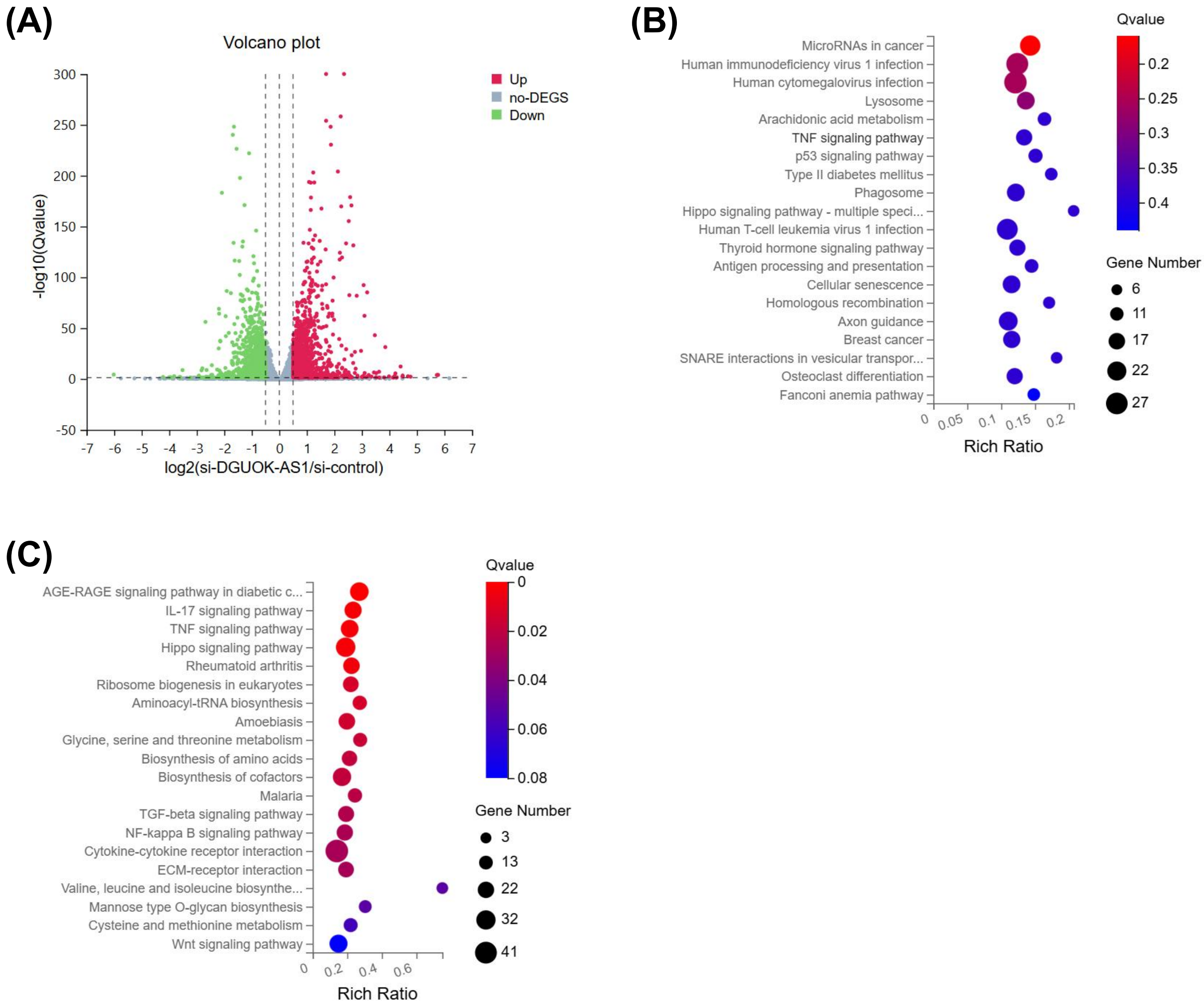

Figure S8 RNA-seq analysis of DGUOK-AS1 knockdown in A549 cells. (A) Volcano plot of DEGs in A549 cells upon DGUOK-AS1 knockdown. KEGG pathway enrichment analysis of downregulated (B) or upregulated (C) genes following DGUOK-AS1 knockdown. ($|log2FC| > 0.5$, $p < 0.05$).

Table S1. The RNA expression data of LncRNA DGUOK-AS1 transcriptions from GEO

ProbeName	P value	T/C	Seq name	Gene Symbol	Source	RNA length	chrom	relationship
ASHGA5P035114	0.0206206432565811	3.938177486	ENST00000413452	DGUOK-AS1	GENCODE	419	chr2	natural antisense
ASHGA5P050525	0.036406197	3.740618513	ENST00000439192	DGUOK-AS1	GENCODE	563	chr2	natural antisense

Table S2. Sanger sequencings of DGUOK-AS1 transcript

Gene	Transcript:	sequence
DGUOK-AS1 201	>ENST00000413452.1	GGAAGCCCTAAGAACTCAAGGGAGATAGGGGTGGCAGGGG<br>CATTTCCCTGGATGCCTGGAGCATGGAGGGTGCGAAATAAGG<br>AGGCTTTCCTACCCCATTGATGAGCTGAAGTTGATTCCGCACG<br>GGGGAGGAAAGGGACAGGAAGCATAATTGGATGTGCAAGCC<br>TGTGGTTCTTCAGGAAGTTAACACGTGGAACTAACACTGTTG<br>GATGCTGCTGAACTCTCAGAAAAATCATCATTGACATCCAACA<br>CCAGCACTGGAATGTTCATCAGAGCCTCAAAGTGGAGCCTATA<br>AGAAGCGTGAACGTTTGGGGGACAGAAAAAAAGAAATGTAA<br>CTTGAGTCAAGTTTCACATACTCGCCAAGTCTTACTCAGAGAA<br>CAGATCTAAATTCAACATGACTTTTCAAAGAAACTGCATGT
DGUOK-AS1 202	>ENST00000439192.1	GCTCCCGCGAGGTCAGAGCCGGGCTGTGCCGCTGCCCCCGG<br>GACTAGAAAGGGAAATCTCCTTGCTAGAGGAGGAAGCCCTAA<br>GAACTCAAGGGAGATAGGGGTGGCAGGGGCATTTCCCTGGA<br>TGCCTGGAGCATGGAGGGTGCGAAATAAGGAGGCTTTCCTAC<br>CCCATTGATGAGCTGAAGTTGATTCCGCACGGGGGAGGAAA<br>GGGACAGGAAGCATAATTGGATGTGCAAGCCTGTGGTTCTTC<br>AGGAAGTTAACACGTGGAACTAACACTGTTGGATGCTGCTGA<br>ACTCTCAGGTGATTGATGTGACTAACCTCCTCAGAAAAATCAT<br>CATTGACATCCAACACCAGCACTGGAATGTTCATCAGAGCCTC<br>AAAGTGGAGCCTATAAGAAGCGTGAACGTTTGGGGGACAGA<br>AAAAAAGAAATGTAACTTGAGTCAAGTTTCACATACTCGCCAA<br>GTCTTACTCAGAGAACAGATCTAAATTCAACATGACTTTTCAA<br>AGAAACTGCATGTAAACTGCATTTAAAAAAACAAATTCAATAA<br>AGTTGGCCGGAAACAG

Table S3. List of candidate miRNAs with potential binding sites on DGUOK-AS1-201 by using miRDB.

Target Rank	Target Score	miRNA Name
1	89	hsa-miR-138-5p
2	84	hsa-miR-4653-5p
3	84	hsa-miR-3921
4	80	hsa-miR-2467-5p
5	77	hsa-miR-1825
6	71	hsa-miR-6761-5p
7	71	hsa-miR-1304-5p
8	70	hsa-miR-26b-3p
9	69	hsa-miR-4502
10	65	hsa-miR-549a-5p
11	61	hsa-miR-4685-3p
12	61	hsa-miR-4287
13	55	hsa-miR-7113-5p
14	52	hsa-miR-4764-5p

Table S4. The result of DGUOK-AS1 RNA pull-down coupled with LC-MS (liquid chromatography/ mass spectrometry).

Accession	Description	Sum PEP Score	Coverage [%]	# Peptides	# PSMs	# Unique Peptides	# AAs	MW [kDa]	calc. pI	Score Sequest HT: Sequest HT	Gene Symbol	Abundances (Normalized): F2: Sample
P11498	Pyruvate carboxylase, mitochondrial OS=Homo sapiens OX=9606 GN=PC PE=1 SV=2	9.399	6	5	7	5	1178	129.6	6.84	14.12	PC	92652999.5
P68363	Tubulin alpha-1B chain OS=Homo sapiens OX=9606 GN=TUBA1B PE=1 SV=1	6.358	12	4	4	4	451	50.1	5.06	8.81	TUBA1B	124691948
P14923	Junction plakoglobin OS=Homo sapiens	8.564	5	2	3	2	745	81.7	6.14	8.33	JUP	19091921.5

	OX=9606 GN=JUP PE=1 SV=3											
P07437	Tubulin beta chain OS=Homo sapiens OX=9606 GN=TUBB PE=1 SV=2	3.244	9	3	3	3	444	49.6	4.89	8.21	TUBB	80913034
P62805	Histone H4 OS=Homo sapiens OX=9606 GN=H4C1 PE=1 SV=2	5.113	22	2	2	2	103	11.4	11.36	5.49	H4C1;	87262924
Q16778	Histone H2B type 2-E OS=Homo sapiens OX=9606 GN=H2BC21 PE=1 SV=3	3.832	12	1	2	1	126	13.9	10.32	4.82	H2BC21	18465162
P81605	Dermcidin OS=Homo sapiens OX=9606 GN=DCD PE=1 SV=2	2.984	13	1	2	1	110	11.3	6.54	4.71	DCD	12933043
P25705	ATP synthase subunit alpha, mitochondrial	4.6	2	1	2	1	553	59.7	9.13	4.23	ATP5F1A	601503856

	OS=Homo sapiens OX=9606 GN=ATP5F1A PE=1 SV=1										
P46783	40S ribosomal protein S10 OS=Homo sapiens OX=9606 GN=RPS10 PE=1 SV=1	4.041	9	1	2	1	165	18.9	10.15	3.84	RPS10	5254256.5
P33764	Protein S100-A3 OS=Homo sapiens OX=9606 GN=S100A3 PE=1 SV=1	8.377	20	1	2	1	101	11.7	4.78	2.61	S100A3	25945500
P02768	Albumin OS=Homo sapiens OX=9606 GN=ALB PE=1 SV=2	2.535	2	1	2	1	609	69.3	6.28	2.51	ALB	12621895
P05109	Protein S100-A8 OS=Homo sapiens OX=9606 GN=S100A8 PE=1 SV=1	1.169	12	1	1	1	93	10.8	7.03	2.27	S100A8	5377205.5
P62987	Ubiquitin-60S	1.082	13	1	1	1	128	14.7	9.83	2.18	UBA52	15206132

	ribosomal protein L40 OS=Homo sapiens OX=9606 GN=UBA52 PE=1 SV=2										
O76031	ATP-dependent Clp protease ATP-binding subunit clpX-like, mitochondrial OS=Homo sapiens OX=9606 GN=CLPX PE=1 SV=2	1.511	2	1	1	1	633	69.2	7.58	2.02	CLPX	83371576
P04406	Glyceraldehyde-3-phosphate dehydrogenase OS=Homo sapiens OX=9606 GN=GAPDH PE=1 SV=3	2.769	4	1	1	1	335	36	8.46	1.89	GAPDH	10469196
P31943	Heterogeneous nuclear ribonucleoprotein H OS=Homo sapiens OX=9606	2.396	3	1	1	1	449	49.2	6.3	1.69	HNRNPH1	10920067

	GN=HNRNPH1 PE=1 SV=4											
P27482	Calmodulin-like protein 3 OS=Homo sapiens OX=9606 GN=CALML3 PE=1 SV=2	2.091	19	2	2	2	149	16.9	4.42	0	CALML3	
P68104	Elongation factor 1-alpha 1 OS=Homo sapiens OX=9606 GN=EEF1A1 PE=1 SV=1	1.881	3	1	1	1	462	50.1	9.01	0	EEF1A1	6242277.5
O75449	Katanin p60 ATPase-containing subunit A1 OS=Homo sapiens OX=9606 GN=KATNA1 PE=1 SV=1	1.478	2	1	2	1	491	55.9	6.9	0	KATNA1	26933294
P15924	Desmoplakin OS=Homo sapiens OX=9606 GN=DSP PE=1 SV=3	1.096	0	1	1	1	2871	331.6	6.81	0	DSP	11725986

P0DPA2	V-set and immunoglobulin domain-containing protein 8 OS=Homo sapiens OX=9606 GN=VSIG8 PE=2 SV=1	1.023	4	1	1	1	414	43.9	7.2	0	VSIG8	
Q01813	ATP-dependent 6-phosphofructokinase, platelet type OS=Homo sapiens OX=9606 GN=PFKP PE=1 SV=2	0.97	3	1	1	1	784	85.5	7.55	0	PFKP	
Q96RT6	cTAGE family member 2 OS=Homo sapiens OX=9606 GN=CTAGE1 PE=1 SV=2	0.893	2	1	1	1	745	85.2	6.16	0	CTAGE1	93739032
O15050	TPR and ankyrin repeat-containing protein 1 OS=Homo sapiens OX=9606	0.893	0	1	1	1	2925	336	6.76	0	TRANK1	36087356

GN=TRANK1
PE=2 SV=4

Table S5. List of primers used in this study.

	Gene Name	Species	Forward	Rerverse
qRT-PCR primers	DGUOK-AS1	Homo sapiens	ACACTGTTGGATGCTGCTGA	GCTCCACTTTGAGGCTCTGA
	DGUOK-AS1 202		GGGAAATCTCCTTGCTAGAGGA	TCAGCAGCATCCAACAGTGT
	miR-2467-5p		TGAGGCTCTGTTAGCCTTGGCT	AGCCAAGGCTAACAGAGCCTCA
	5S rRNA		GCCATACCACCCTGAACG	CGAACATGTACAGTCCATGGATAG
	PRMT5		GTACATCTATGGGCTGTGATTCC	GATATTCCAGGGAGTTCTTGAGG
	RBM15		GGCTGCCTGAGGAGAGTGGAG	CGGCTACTGCTCAATTCTGGACTG
	HNRNPH1		GTCCAAATAGTCCTGACACGG	TCCACCGGCAATGTTATCCC
	U6		CTCGCTTCGGCAGCACA	AACGCTTCACGAATTTGCGT
	GAPDH		GTGAACCATGAGAAGTATG	CGGCCATCACGCCACAGTTTC
PCR primers	DGUOK-AS1 201	Homo sapiens	CGGATATCGGACCAAGCAGGCAGCTTC	TTGCGGCCGCTGGAGCACAACCCATACCCC
	T7-201-Exon1		TAATACGACTCACTATAGGGGGAAGCCCTAAGAACTCA	CTGAGAGTTCAGCAGCATCCAA
	T7-201-Exon2		CGCCTAATACGACTCACTATAGGGAAAAATCATCATTGACATCC	ACATGCAGTTTCTTTGAAAAGTC
	T7-201-Exon1+2		TAATACGACTCACTATAGGGGGAAGCCCTAAGAACTCAACATGCAGTTTCTTTGAAAAGTC	

	DGUOK-AS1 202		CCGGATATCGTGACTTCGCGCCG	TTGCGGCCGCACAAATAAAGCA TGTGTTTATTGAAA
	T7-202-Exon1		TAATACGACTCACTATAGGGGCTCCCG CGAGGTCAGAG	CTGAGGAGGTTAGTCACATC
	T7-202-Exon2		CGCCTAATACGACTCACTATAGGGAAA AATCATCATTGACATCC	CTGTTTCCGGCCAACTTTATTG
	T7-202-Exon1+2		TAATACGACTCACTATAGGGGCTCCCG CGAGGTCAGAG	CTGTTTCCGGCCAACTTTATTG

Table S6. List of siRNA and microRNA used in this study.

	**Name**	**Species**	**Sense**	**Antisense**
**siRNAs**	Negative control	Homo sapiens	UUCUCCGAACGUGUCACGUTT	ACGUGACACGUUCGGAGAATT
	si-DGUOK-AS1		CAUGGAGGGUGCGAAAUAATT	UUAUUUCGCACCCUCCAUGTT
			CUGUGGUUCUUCAGGAAGUTT	ACUUCCUGAAGAACCACAGTT
	si-PRMT5		GGGACUGGAAUACGCUAAUTT	AUUAGCGUAUUCCAGUCCCTT
			GGACCUGAGAGAUGAUAUATT	UAUAUCAUCUCUCAGGUCCTT
	si-HNRNPH1		GCUCAAGGUAUUCGUUUCATT	UGAAACGAAUACCUUGAGCTT
			GGAUUACCUUACAGAGCUATT	UAGCUCUGUAAGGUAAUCCTT
**microRNA mimics**	NC-mimic	Homo sapiens	UUCUCCGAACGUGUCACGUTT	ACGUGACACGUUCGGAGAATT
	miR-2467-5p mimic		UGAGGCUCUGUUAGCCUUGGCUC	GCCAAGGCUAACAGAGCCUCAU U

Table S7: List of FISH probe used in this study.

	Gene Name	species	Probe sequence	Probe modification
**DGUOK-AS1 transcriptions RNA-FISH probe**	DGUOK-AS1	Homo sapiens	TCCT+TATT+TCGCACCCTCCA	5'-Biotin
	DGUOK-AS1 202		TGAGGAGGT+TAGTCACA+TCAATCA	5'-Biotin

Table S8. Patient Information

Sample	Sex	Age(year)	Ancestry/race/ethnicity	Cancer subtype
1	Male	62	East Asian Ancestry	Lung cancer
2	Male	60	East Asian Ancestry	Normal
3	Female	58	East Asian Ancestry	Lung cancer
4	Female	60	East Asian Ancestry	Normal
5	Female	27	East Asian Ancestry	Lung cancer
6	Female	25	East Asian Ancestry	Normal
7	Male	60	East Asian Ancestry	Lung cancer
8	Male	57	East Asian Ancestry	Normal
9	Male	60	East Asian Ancestry	Lung cancer
10	Male	58	East Asian Ancestry	Normal
11	Male	55	East Asian Ancestry	Lung cancer
12	Male	55	East Asian Ancestry	Normal
13	Female	61	East Asian Ancestry	Lung cancer
14	Female	58	East Asian Ancestry	Normal
15	Male	60	East Asian Ancestry	Lung cancer
16	Male	64	East Asian Ancestry	Normal
17	Female	72	East Asian Ancestry	Lung cancer
18	Female	73	East Asian Ancestry	Normal
19	Male	50	East Asian Ancestry	Lung cancer
20	Male	49	East Asian Ancestry	Normal
21	Male	65	East Asian Ancestry	Lung cancer
22	Male	65	East Asian Ancestry	Normal
23	Male	74	East Asian Ancestry	Lung cancer
24	Male	75	East Asian Ancestry	Normal
25	Female	59	East Asian Ancestry	Lung cancer
26	Female	58	East Asian Ancestry	Normal
27	Female	63	East Asian Ancestry	Lung cancer
28	Female	64	East Asian Ancestry	Normal
29	Female	66	East Asian Ancestry	Lung cancer
30	Female	65	East Asian Ancestry	Normal
31	Male	74	East Asian Ancestry	Lung cancer
32	Male	75	East Asian Ancestry	Normal
33	Female	28	East Asian Ancestry	Lung cancer
34	Female	30	East Asian Ancestry	Normal
35	Female	50	East Asian Ancestry	Lung cancer
36	Female	50	East Asian Ancestry	Normal
37	Female	58	East Asian Ancestry	Lung cancer
38	Female	58	East Asian Ancestry	Normal
39	Male	79	East Asian Ancestry	Lung cancer
40	Male	76	East Asian Ancestry	Normal
41	Male	50	East Asian Ancestry	Lung cancer
42	Male	50	East Asian Ancestry	Normal

43	Male	52	East Asian Ancestry	Lung cancer
44	Male	53	East Asian Ancestry	Normal
45	Male	56	East Asian Ancestry	Lung cancer
46	Male	57	East Asian Ancestry	Normal
47	Male	54	East Asian Ancestry	Lung cancer
48	Male	53	East Asian Ancestry	Normal
49	Male	59	East Asian Ancestry	Lung cancer
50	Male	58	East Asian Ancestry	Normal
51	Male	62	East Asian Ancestry	Lung cancer
52	Male	63	East Asian Ancestry	Normal
53	Male	64	East Asian Ancestry	Lung cancer
54	Male	63	East Asian Ancestry	Normal
55	Male	69	East Asian Ancestry	Lung cancer
56	Male	69	East Asian Ancestry	Normal
57	Male	79	East Asian Ancestry	Lung cancer
58	Male	74	East Asian Ancestry	Normal
59	Female	44	East Asian Ancestry	Lung cancer
60	Female	44	East Asian Ancestry	Normal
61	Female	50	East Asian Ancestry	Lung cancer
62	Female	49	East Asian Ancestry	Normal
63	Female	50	East Asian Ancestry	Lung cancer
64	Female	49	East Asian Ancestry	Normal
65	Female	60	East Asian Ancestry	Lung cancer
66	Female	61	East Asian Ancestry	Normal
67	Male	58	East Asian Ancestry	Lung cancer
68	Male	58	East Asian Ancestry	Normal
69	Male	59	East Asian Ancestry	Lung cancer
70	Male	59	East Asian Ancestry	Normal
71	Male	65	East Asian Ancestry	Lung cancer
72	Male	65	East Asian Ancestry	Normal
73	Male	69	East Asian Ancestry	Lung cancer
74	Male	69	East Asian Ancestry	Normal
75	Male	72	East Asian Ancestry	Lung cancer
76	Male	70	East Asian Ancestry	Normal
77	Male	58	East Asian Ancestry	Lung cancer
78	Male	58	East Asian Ancestry	Normal
79	Male	62	East Asian Ancestry	Lung cancer
80	Male	59	East Asian Ancestry	Normal
81	Male	69	East Asian Ancestry	Lung cancer
82	Male	69	East Asian Ancestry	Normal
83	Male	69	East Asian Ancestry	Lung cancer
84	Male	70	East Asian Ancestry	Normal
85	Male	61	East Asian Ancestry	Lung cancer
86	Male	60	East Asian Ancestry	Normal

Data S1：Original Western blot images

Corresponding to **Figure 3N** of the main text

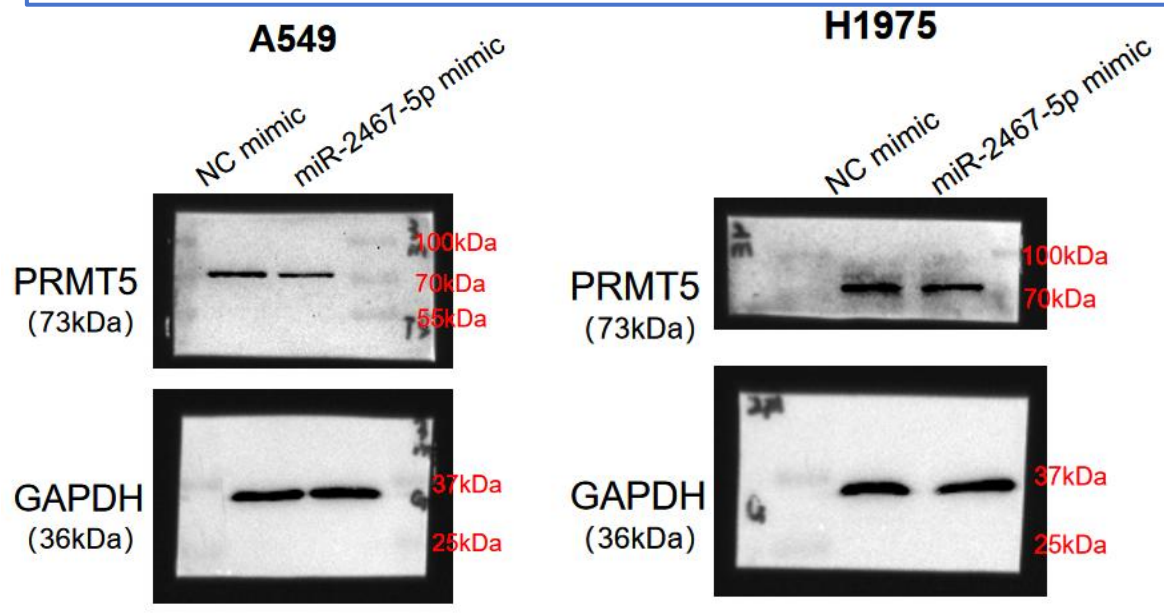

(Repeat)Corresponding to **Figure 3N** of the main text

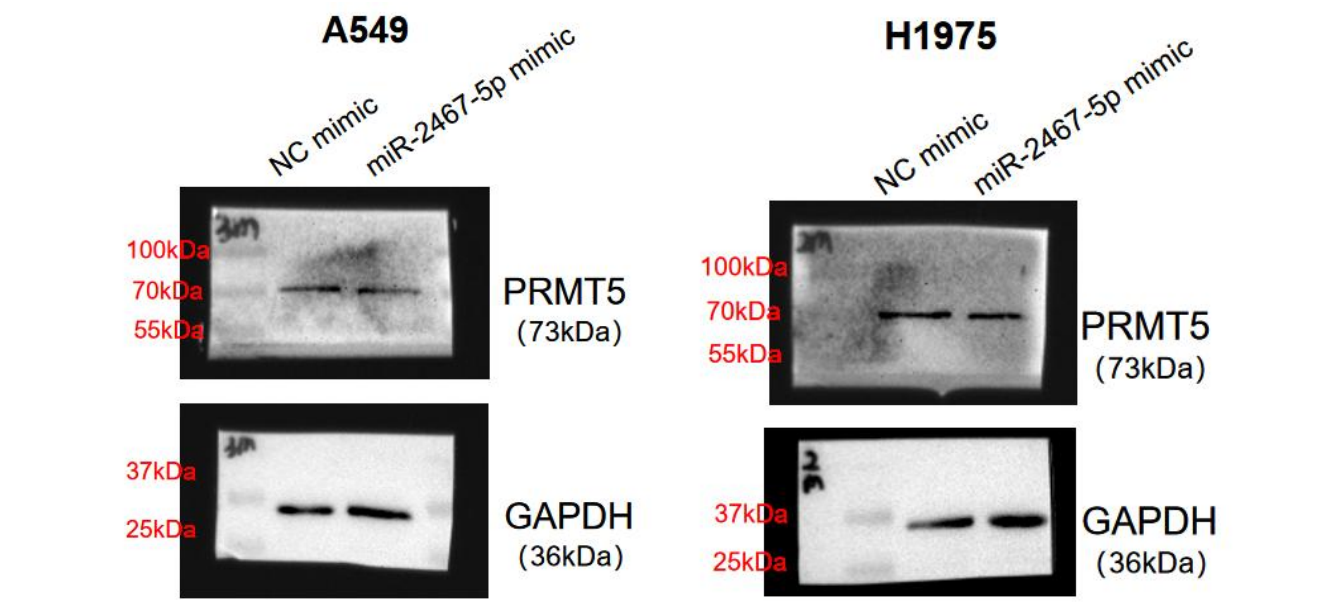

Corresponding to **Figure 3O** of the main text

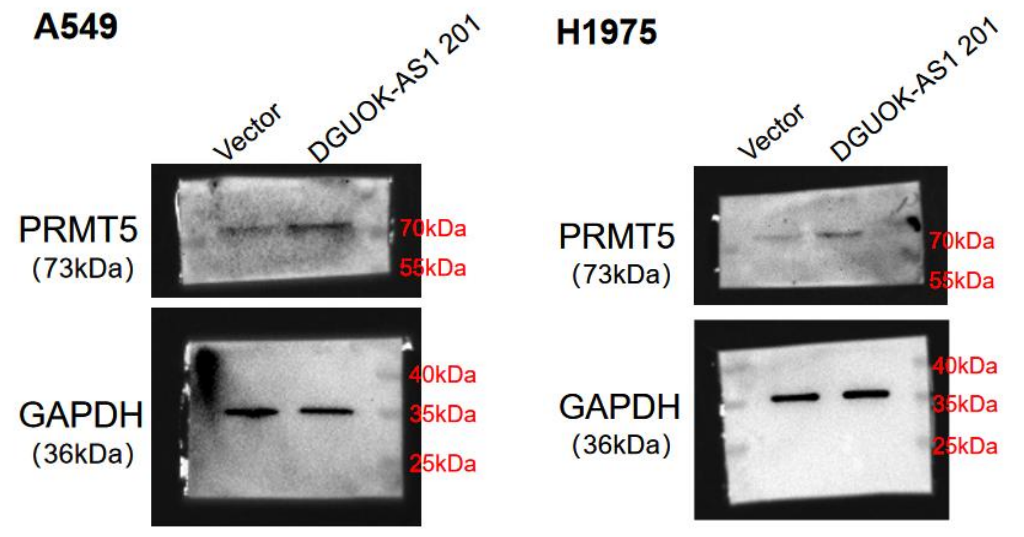

(Repeat)Corresponding to **Figure 3O** of the main text

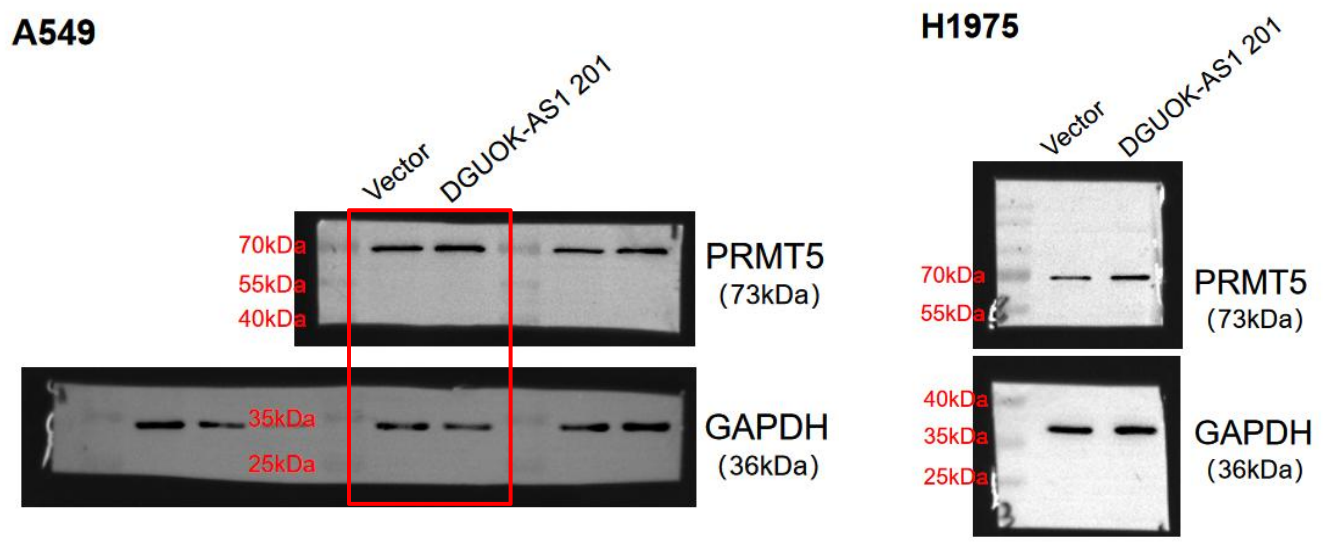

Corresponding to **Figure 4F** of the main text

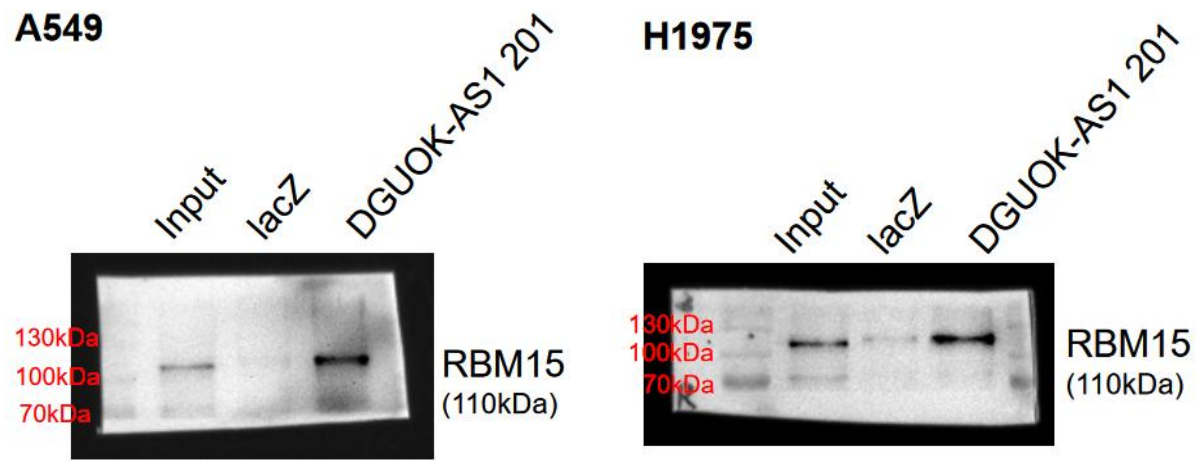

(Repeat)Corresponding to **Figure 4F** of the main text

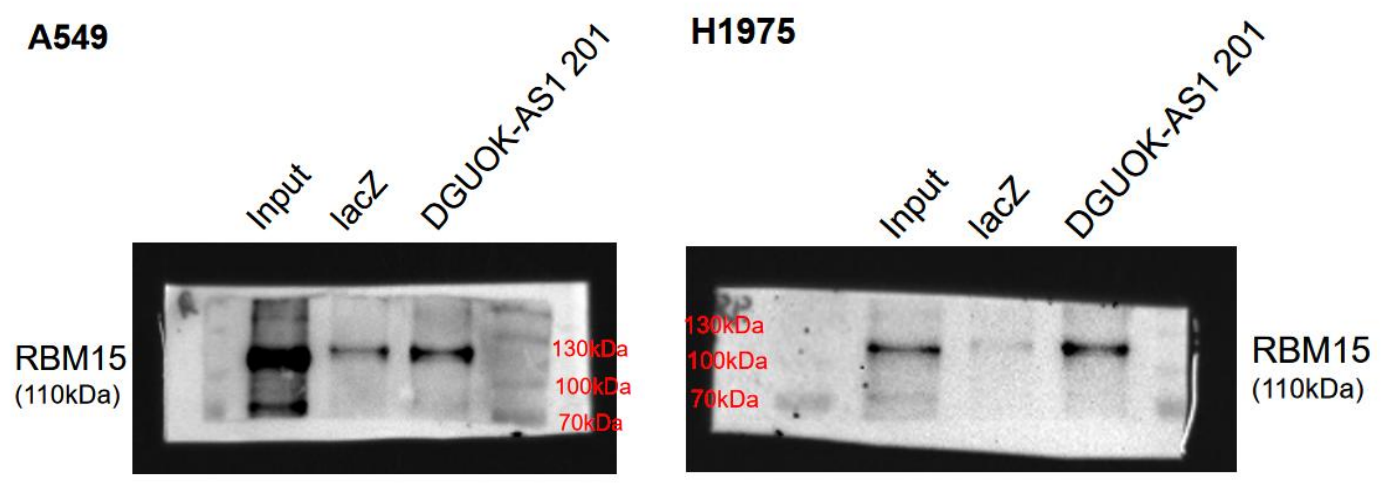

Corresponding to **Figure 5K** of the main text

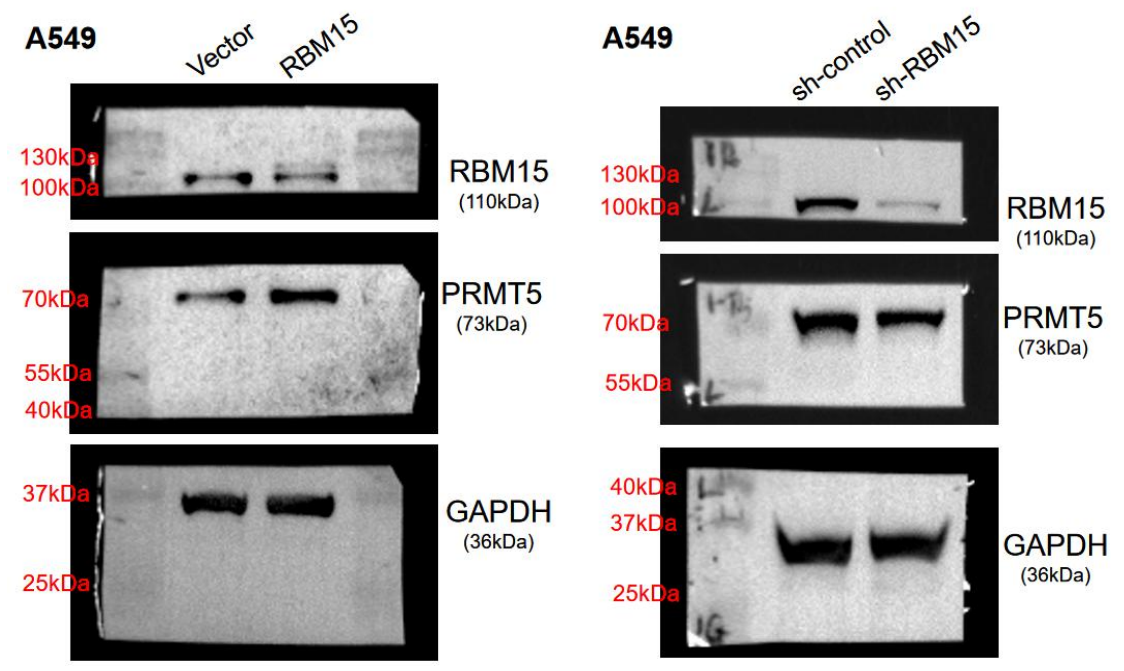

(Repeat)Corresponding to **Figure 5K** of the main text

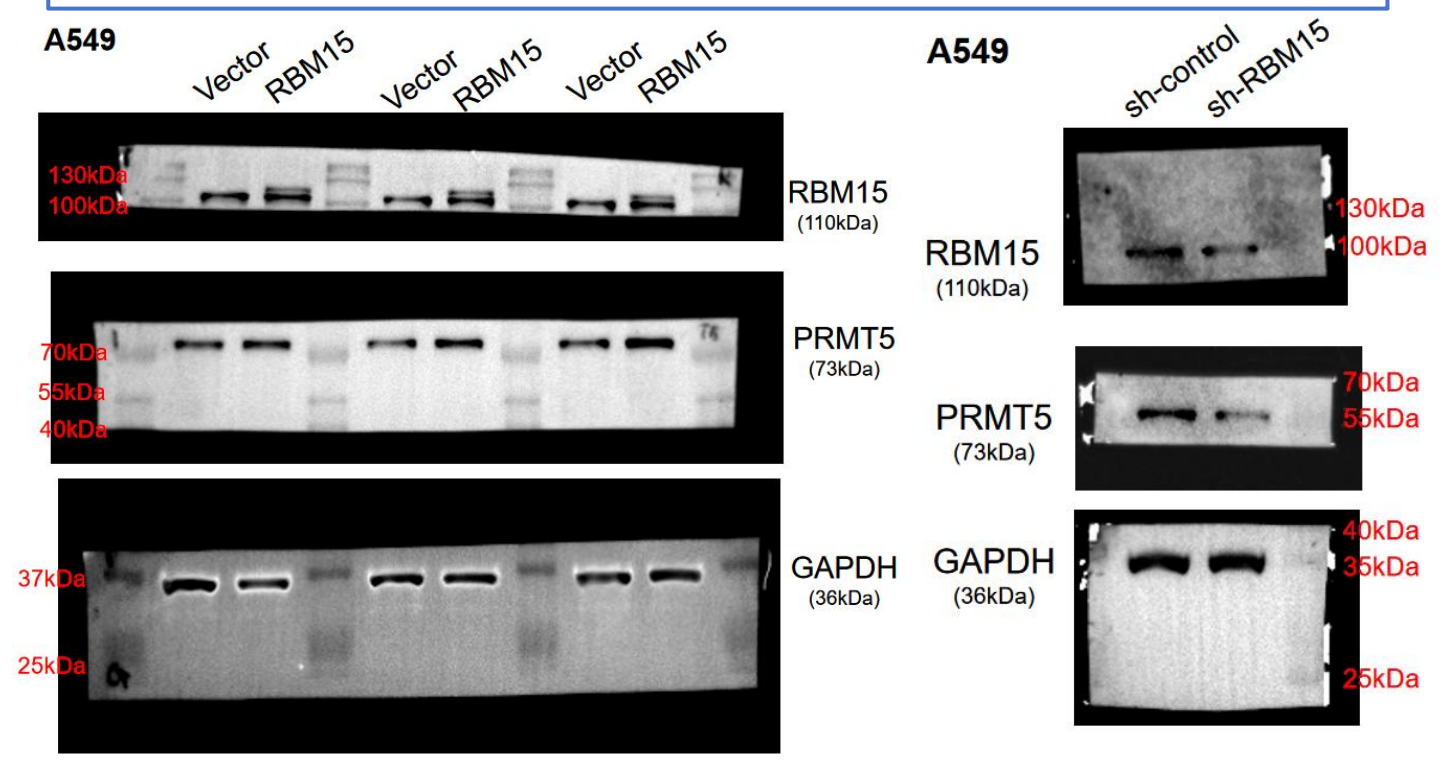

Corresponding to **Figure 6B** of the main text

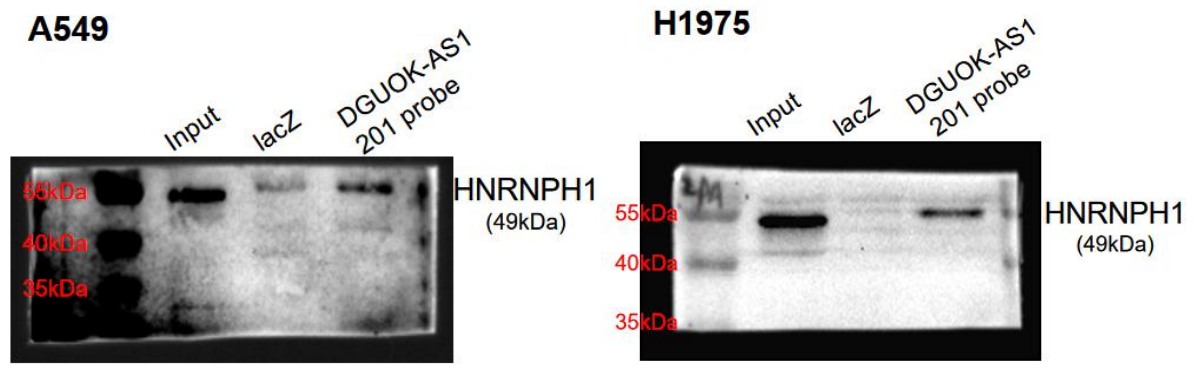

(Repeat)Corresponding to **Figure 6B** of the main text

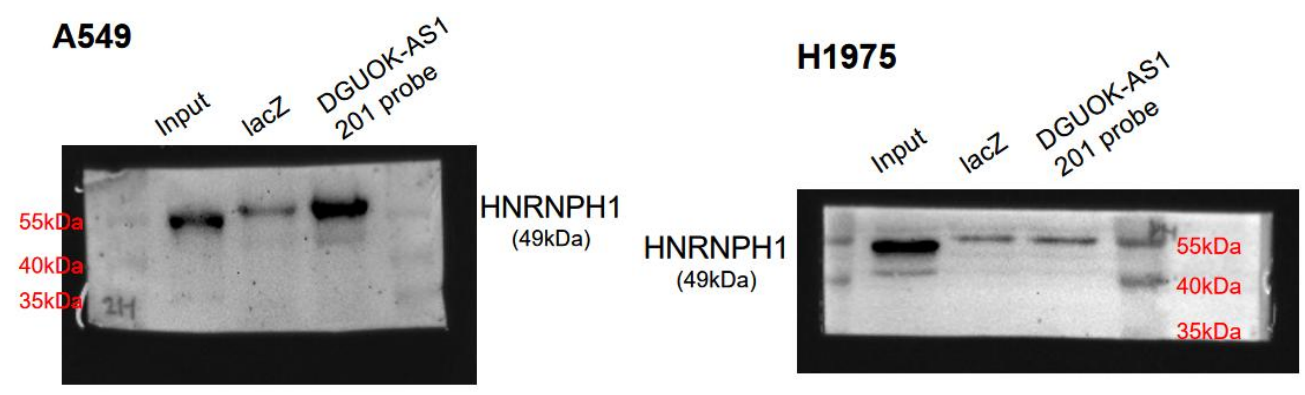

Corresponding to **Figure 7H** of the main text

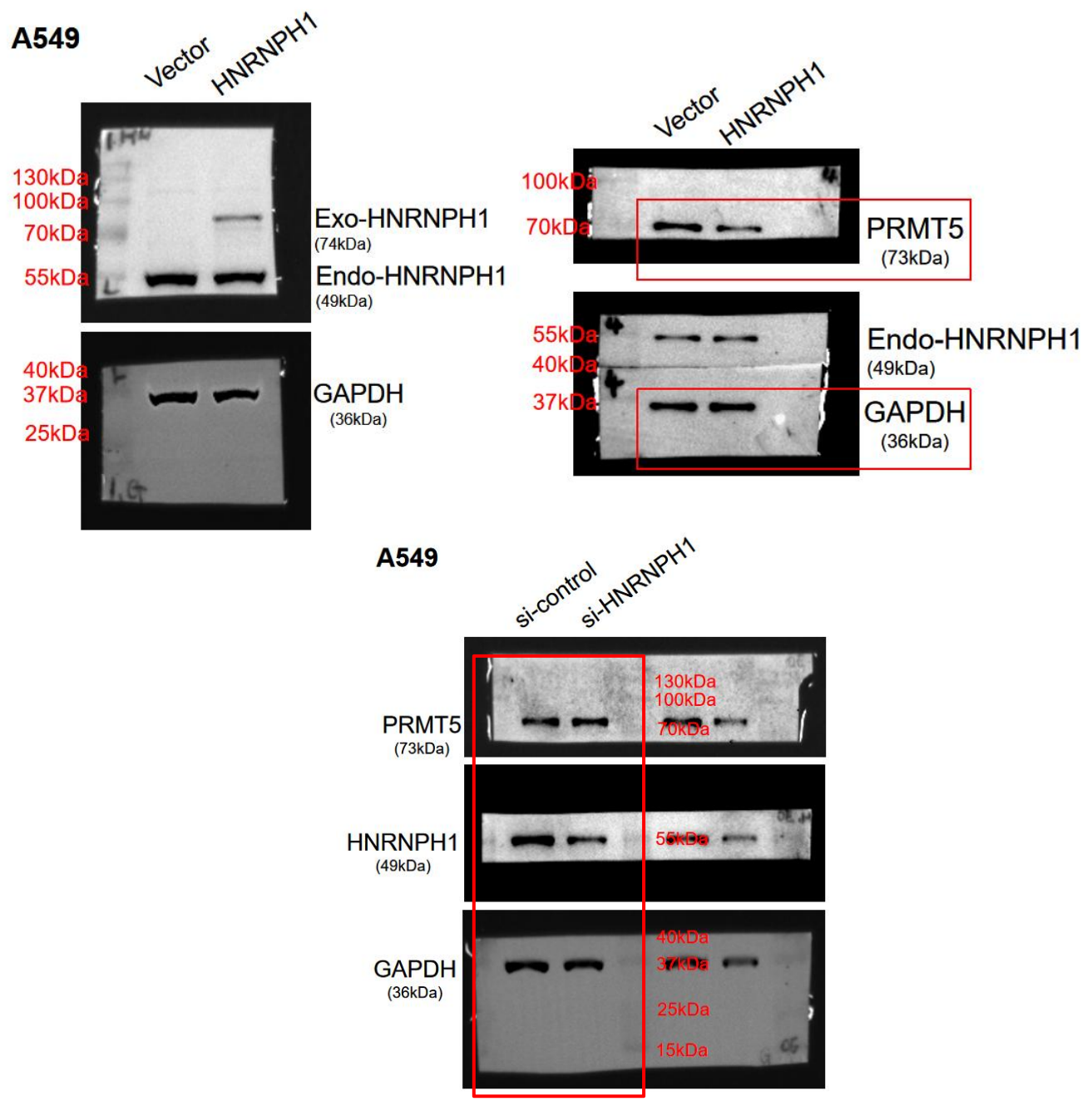

(Repeat)Corresponding to **Figure 7H** of the main text

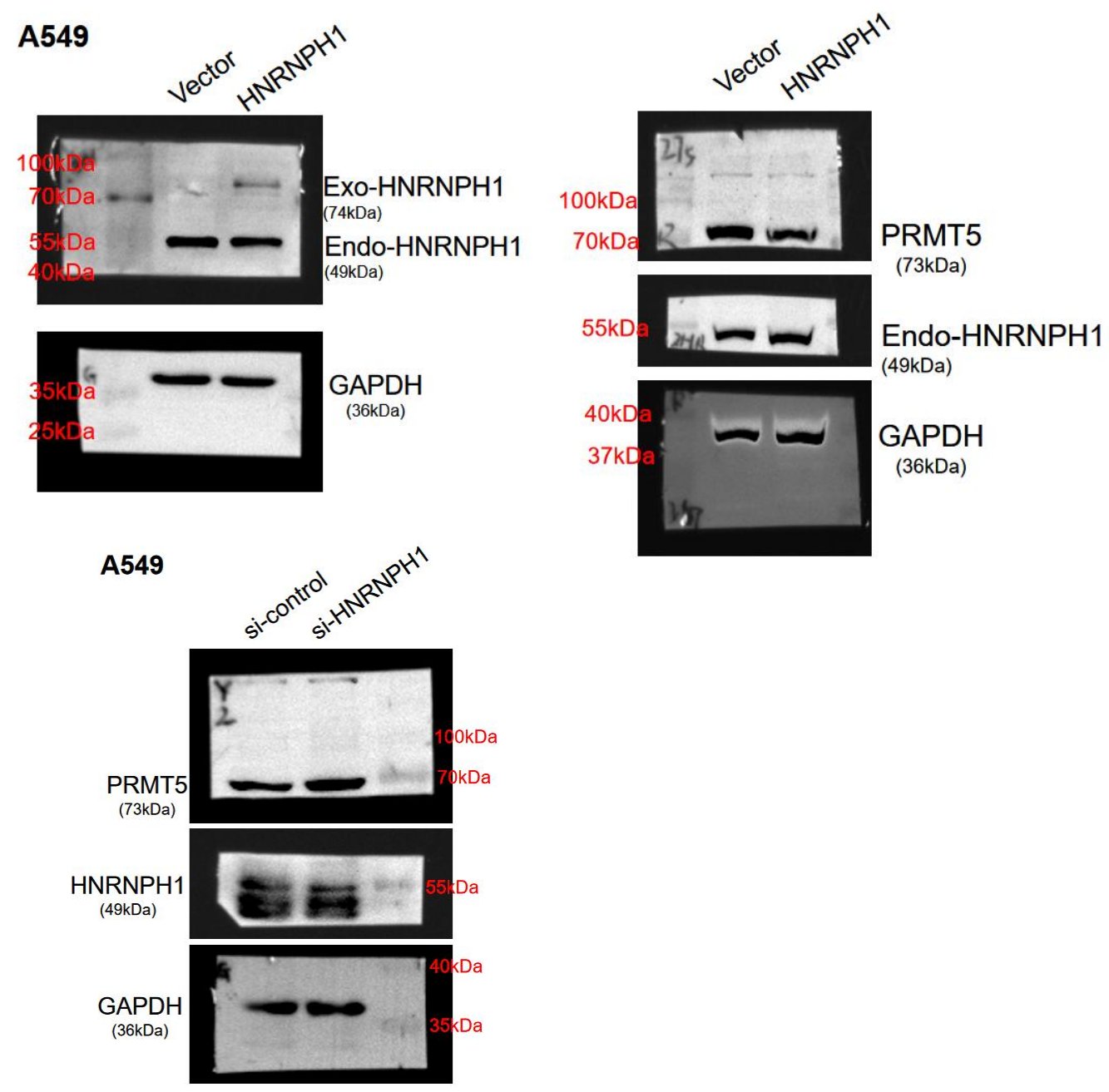

Corresponding to **Figure S3G** of the main text

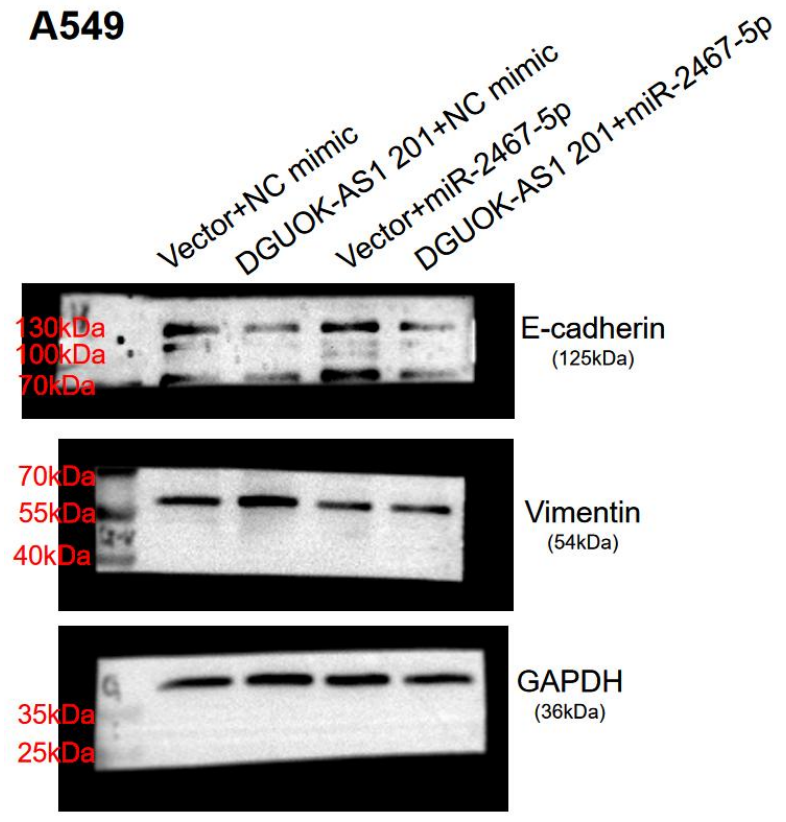

(Repeat)Corresponding to **Figure S3G** of the main text

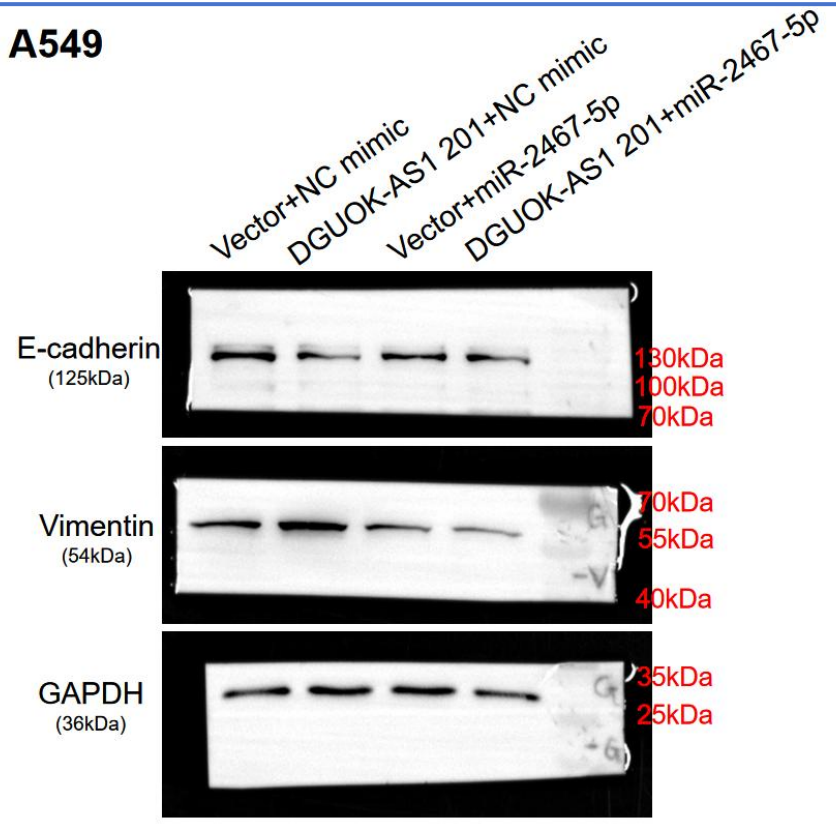

Corresponding to **Figure S4A** of the main text

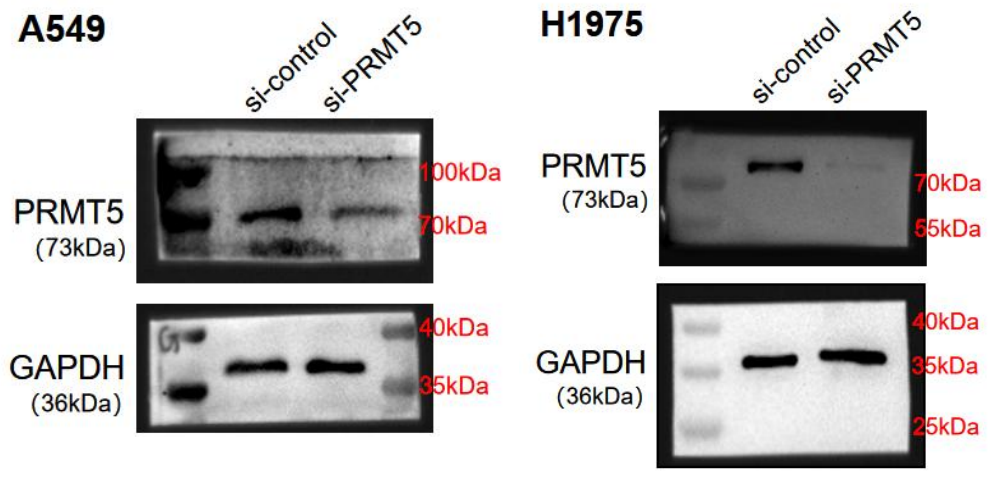

(Repeat)Corresponding to **Figure S4A** of the main text

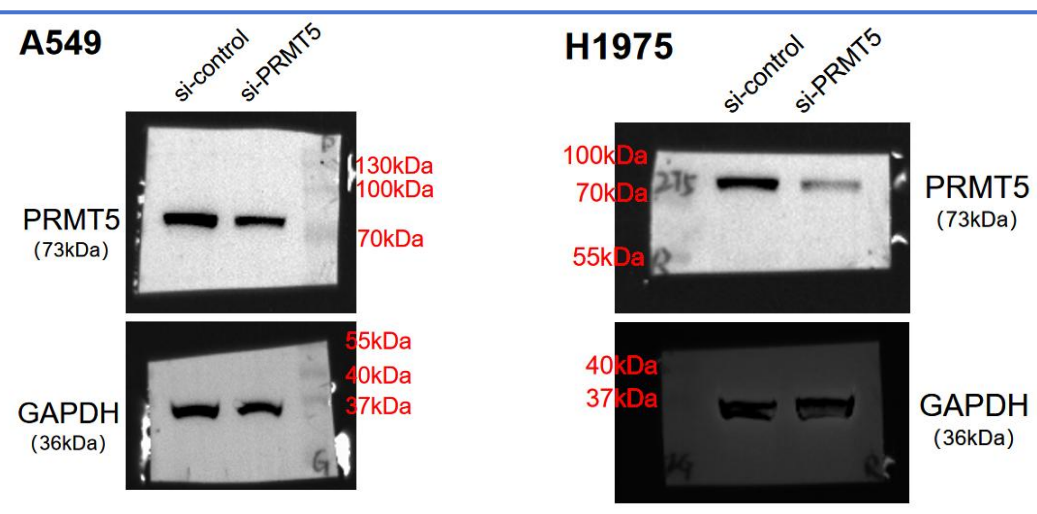

Corresponding to **Figure S4E** of the main text

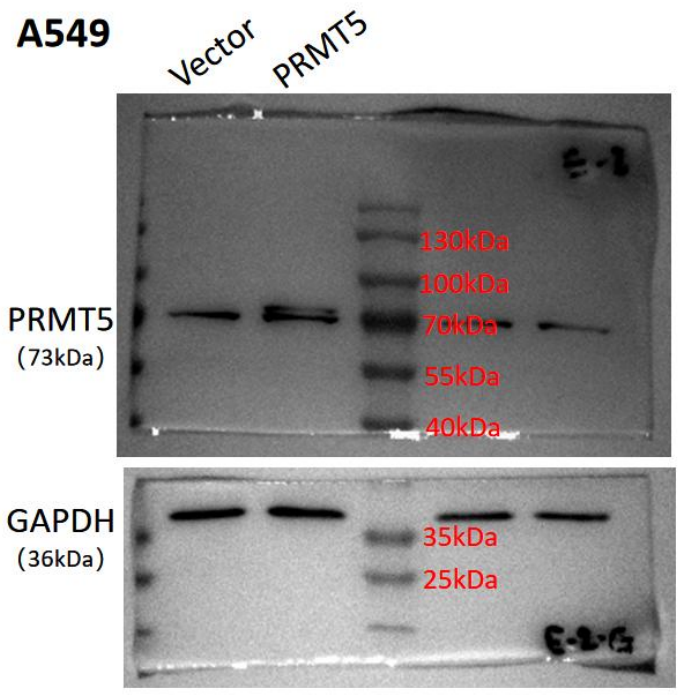

(Repeat)Corresponding to **Figure S4E** of the main text

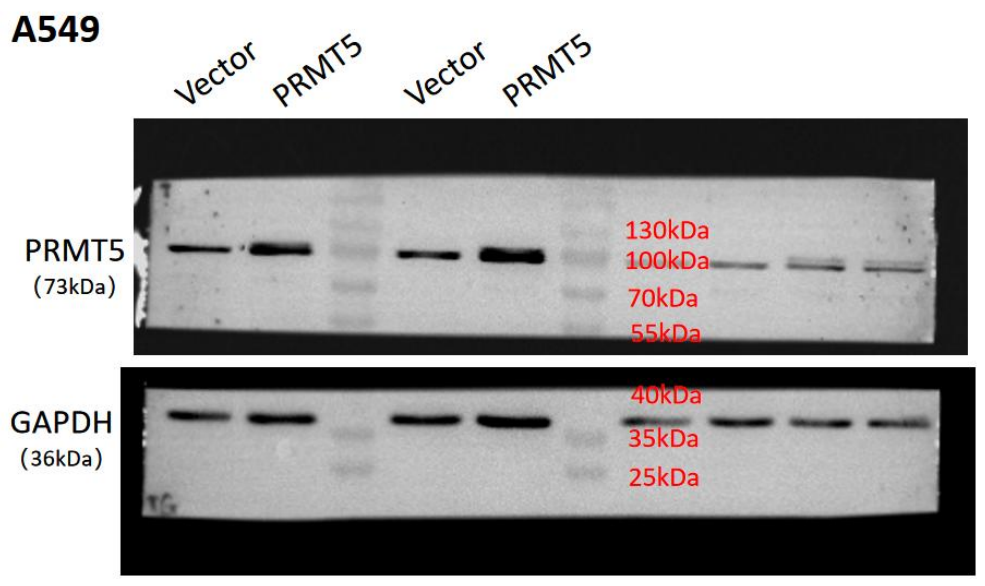

Corresponding to **Figure S4J** of the main text

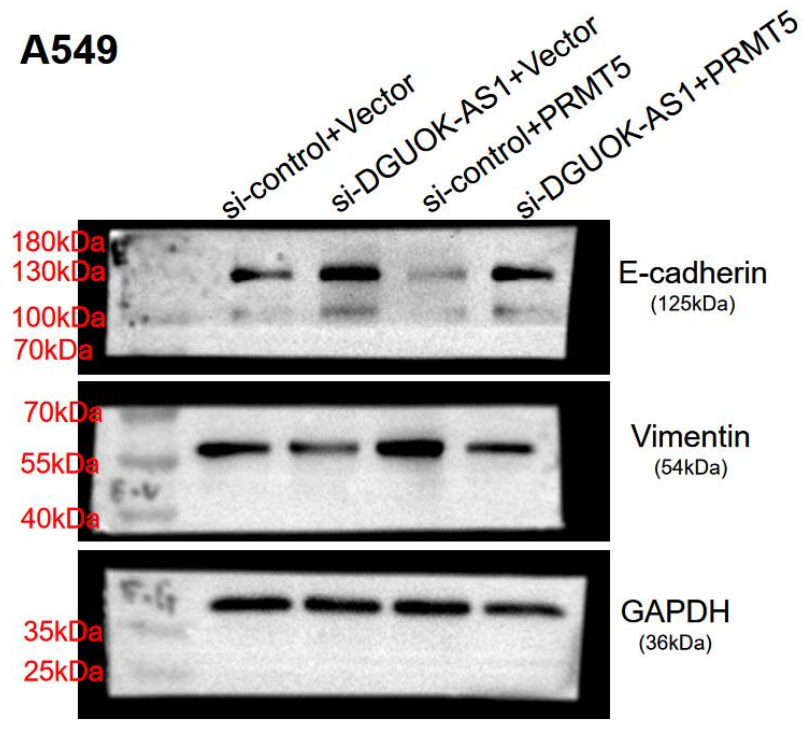

(Repeat)Corresponding to **Figure S4J** of the main text

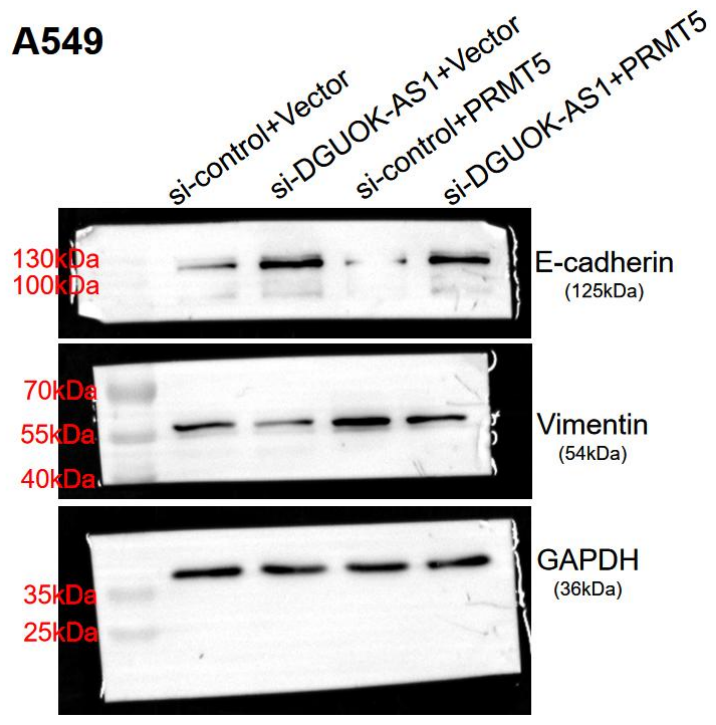

Corresponding to **Figure S5D** of the main text

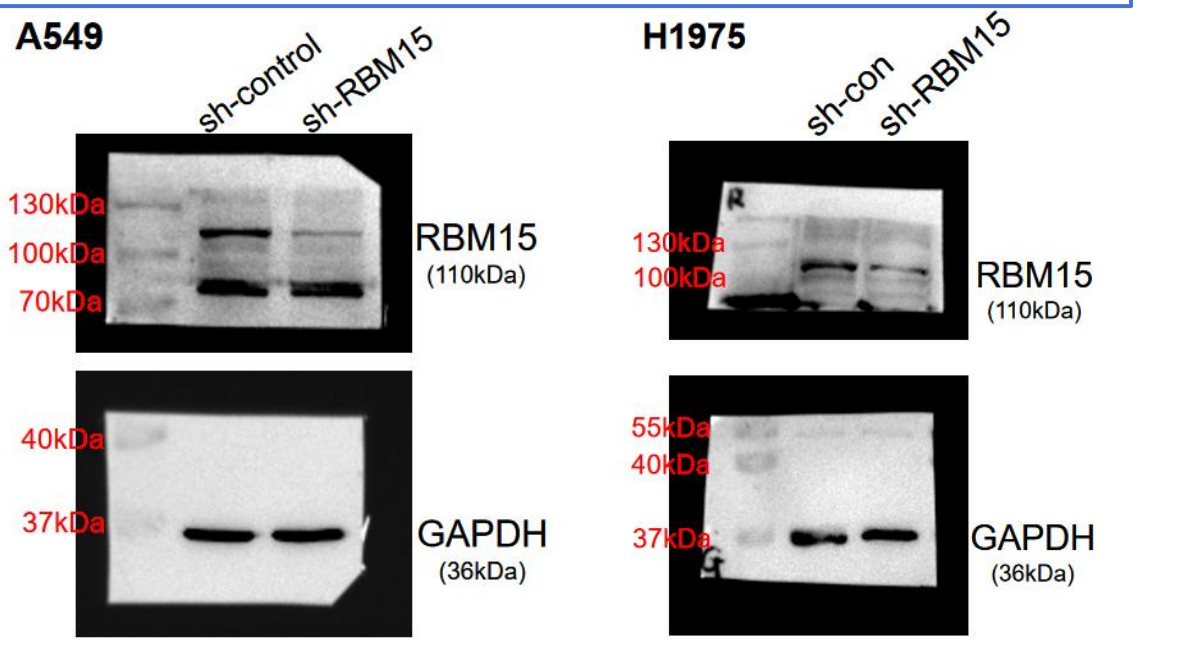

(Repeat)Corresponding to **Figure S5D** of the main text

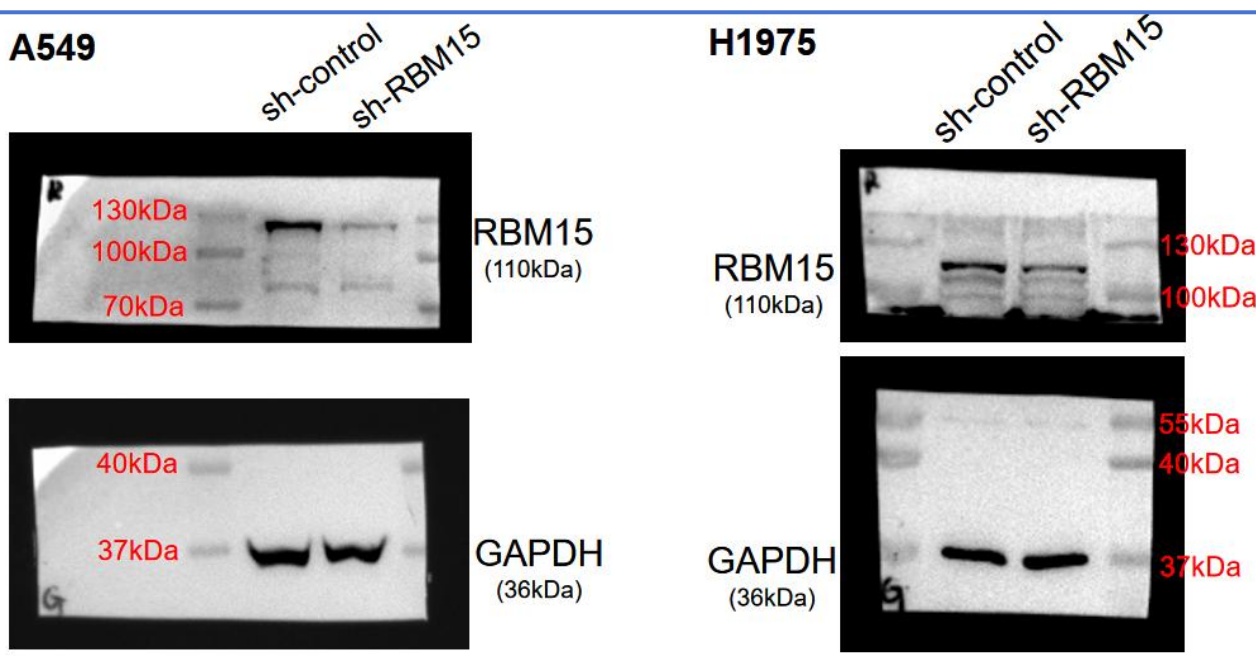

Corresponding to **Figure S5E** of the main text

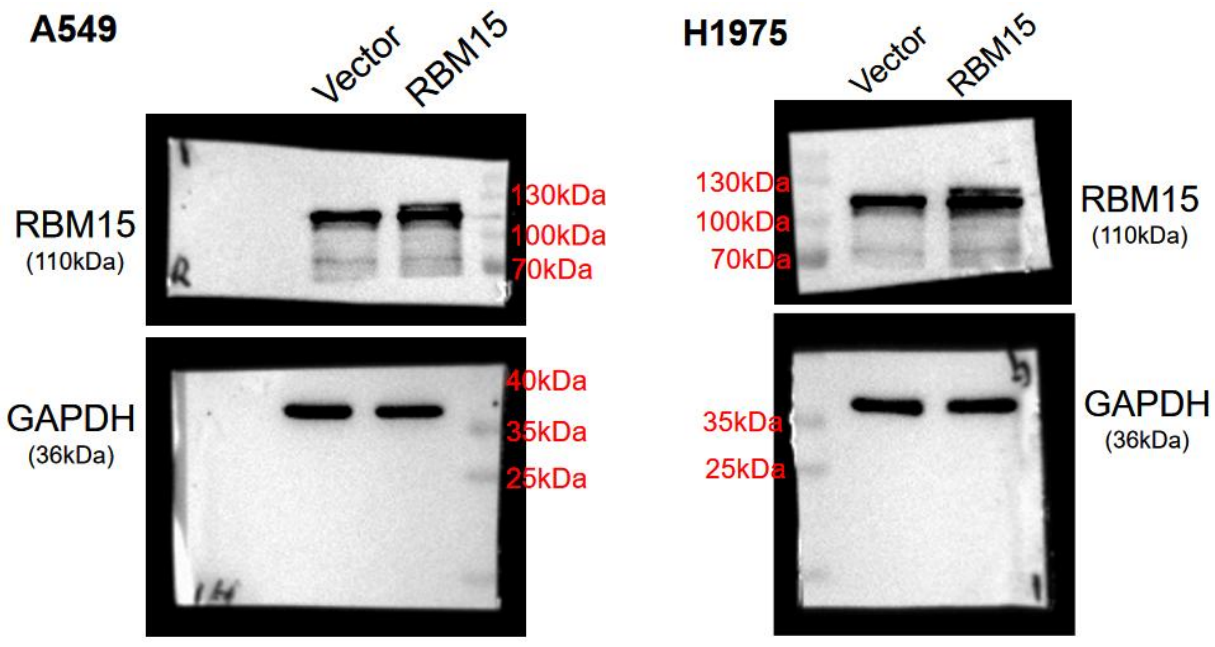

(Repeat)Corresponding to **Figure S5E** of the main text

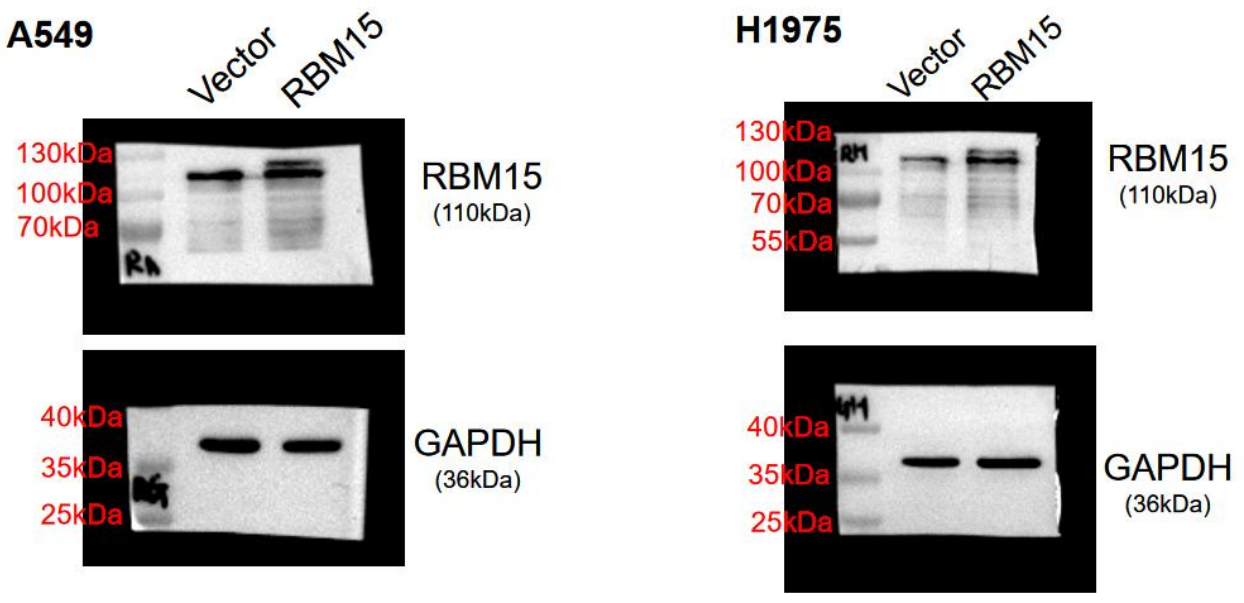

Corresponding to **Figure S5J** of the main text

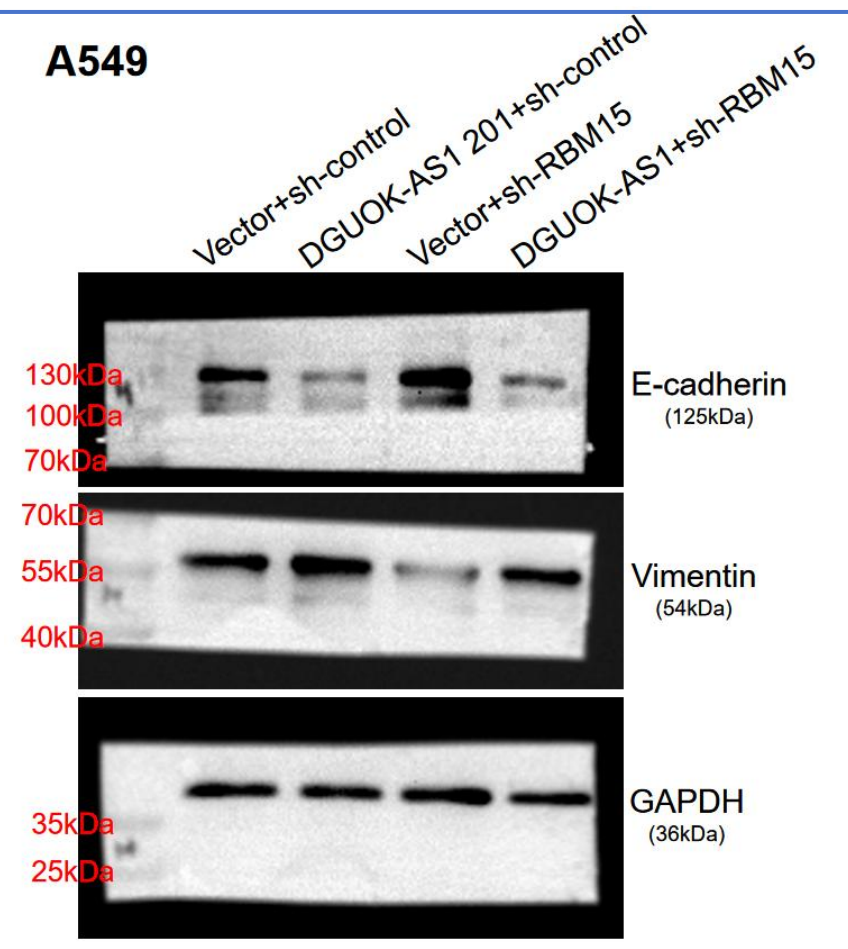

(Repeat)Corresponding to **Figure S5J** of the main text

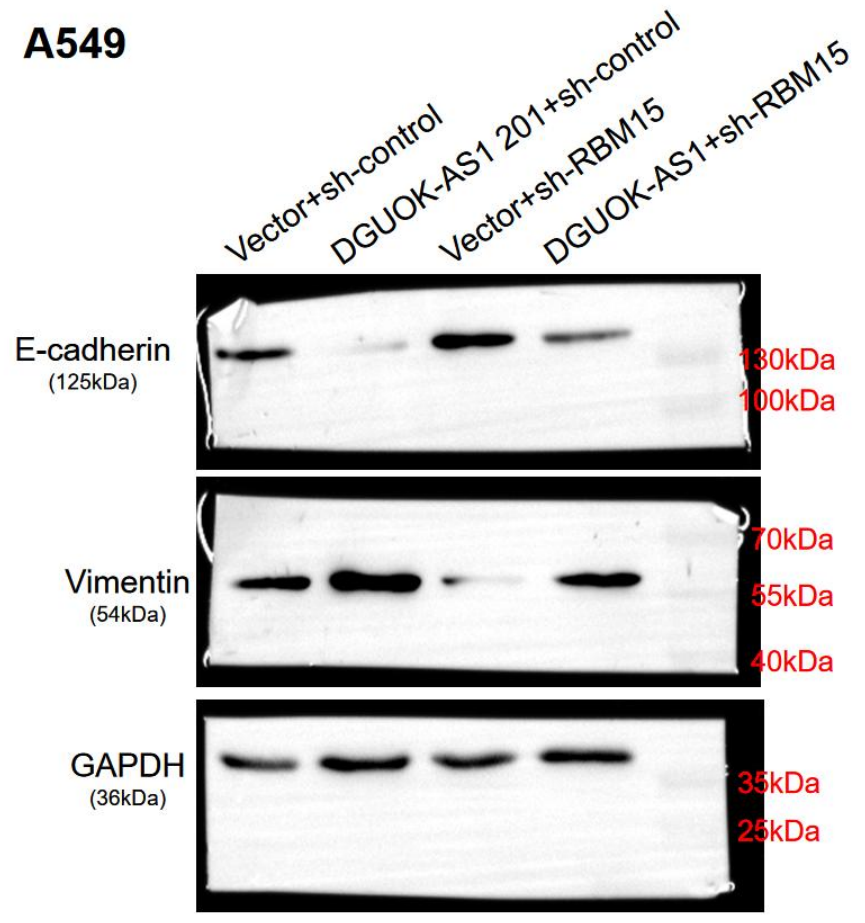

Corresponding to **Figure S6C** of the main text

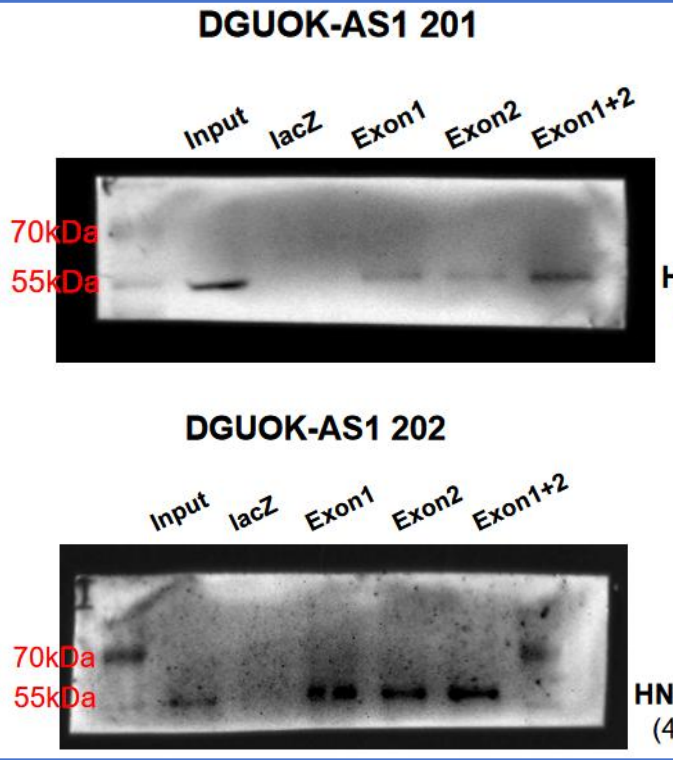

(Repeat)Corresponding to **Figure S6C** of the main text

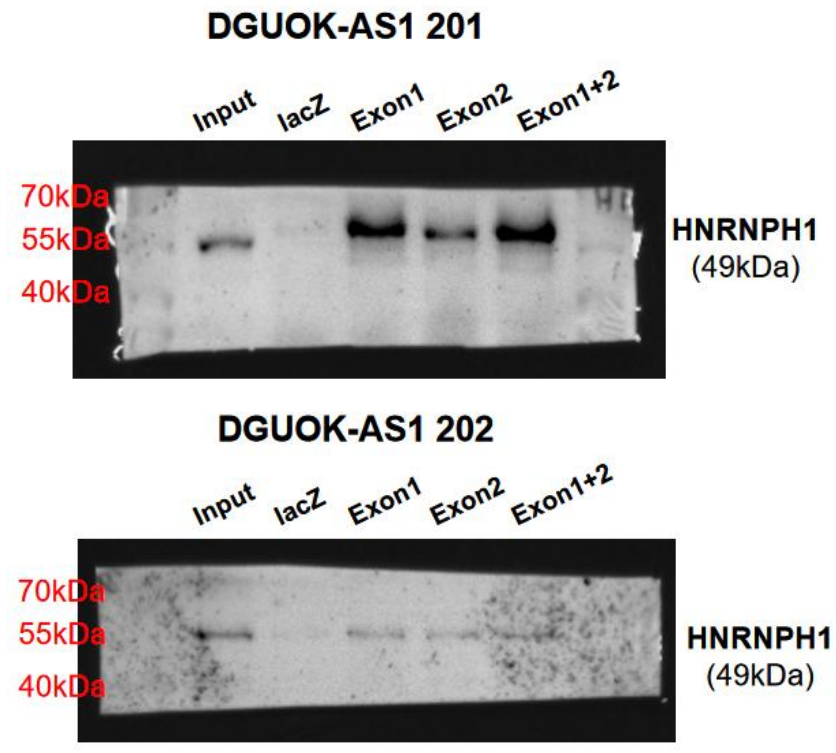

Corresponding to **Figure S7C** of the main text

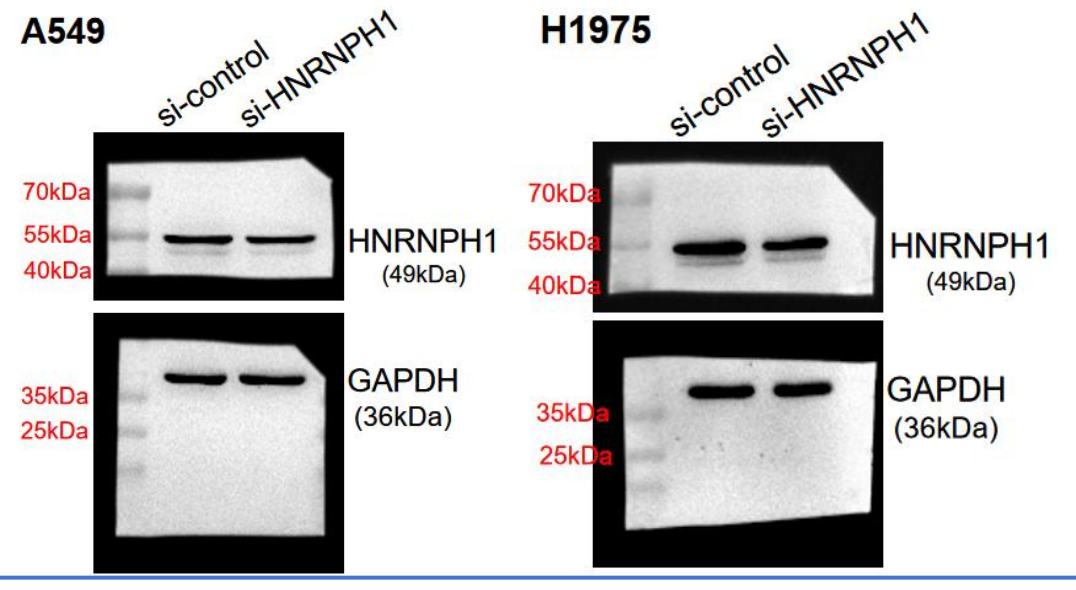

(Repeat)Corresponding to **Figure S7C** of the main text

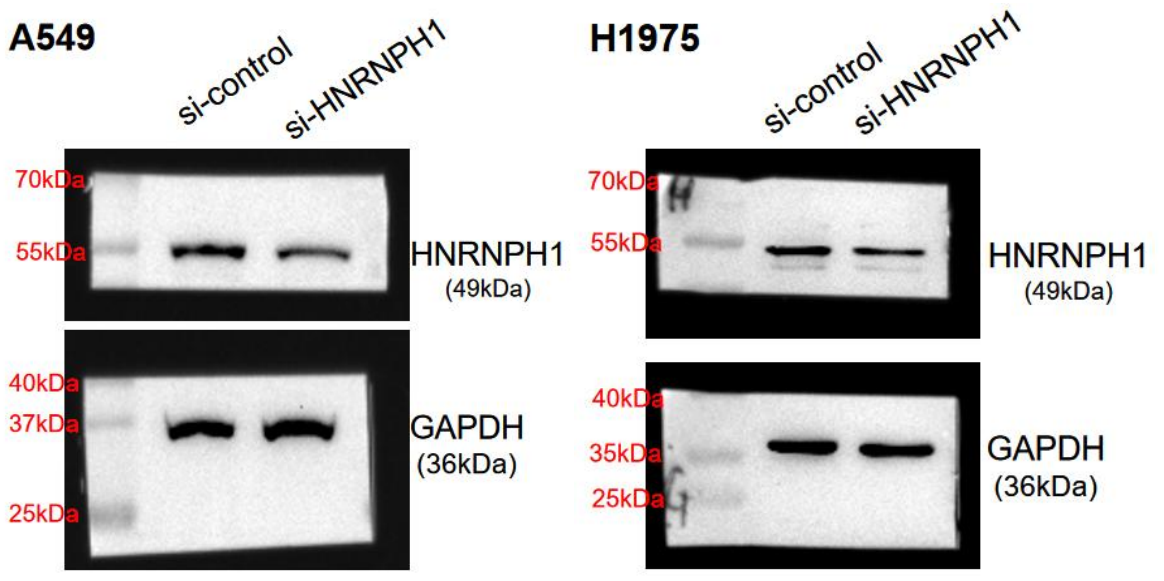

Corresponding to **Figure S7D** of the main text

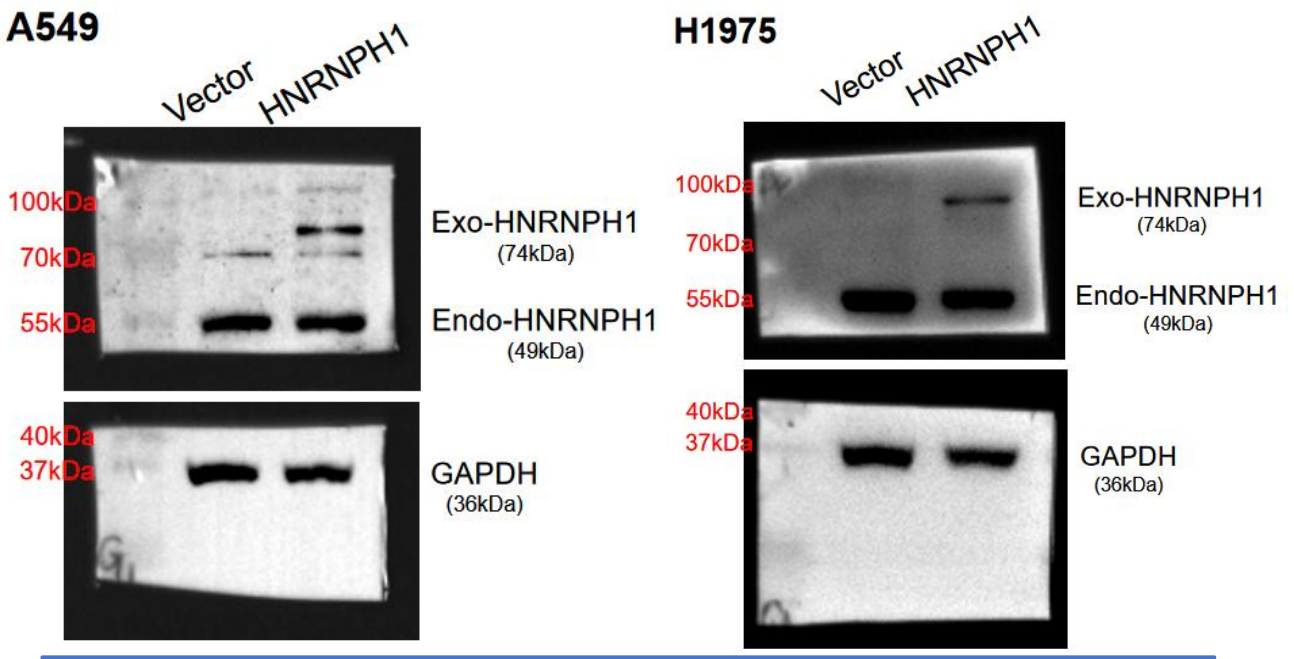

(Repeat)Corresponding to **Figure S7D** of the main text

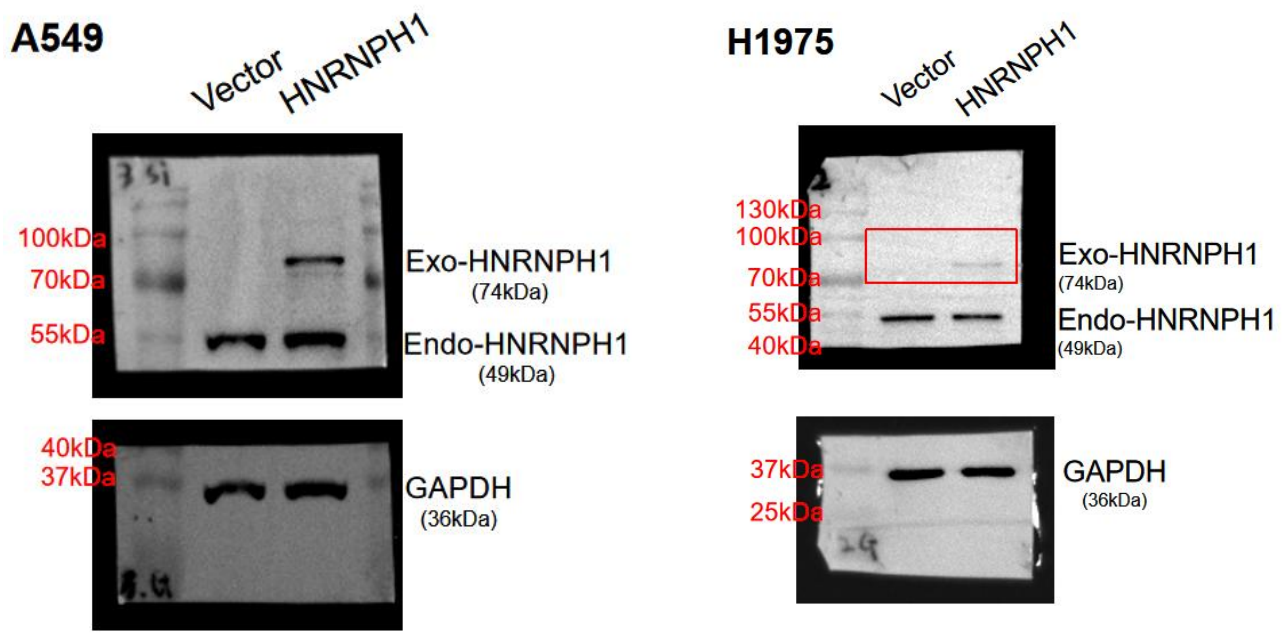

Corresponding to **Figure S7G** of the main text

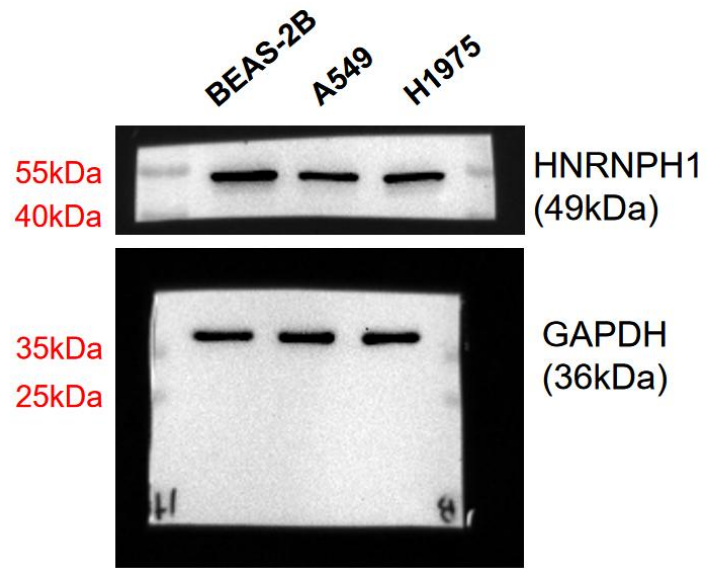

(Repeat)Corresponding to **Figure S7G** of the main text

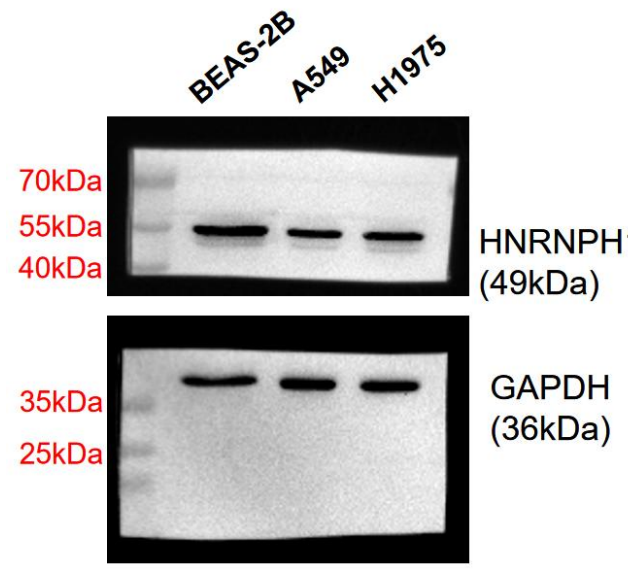

Corresponding to **Figure S7H** of the main text

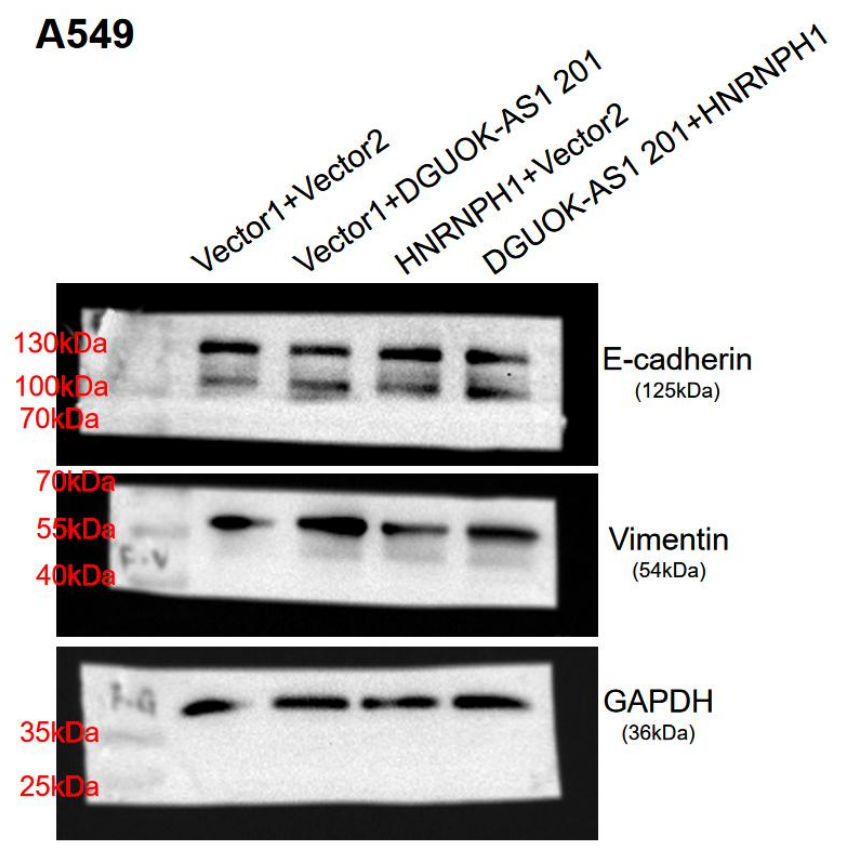

(Repeat)Corresponding to **Figure S7H** of the main text

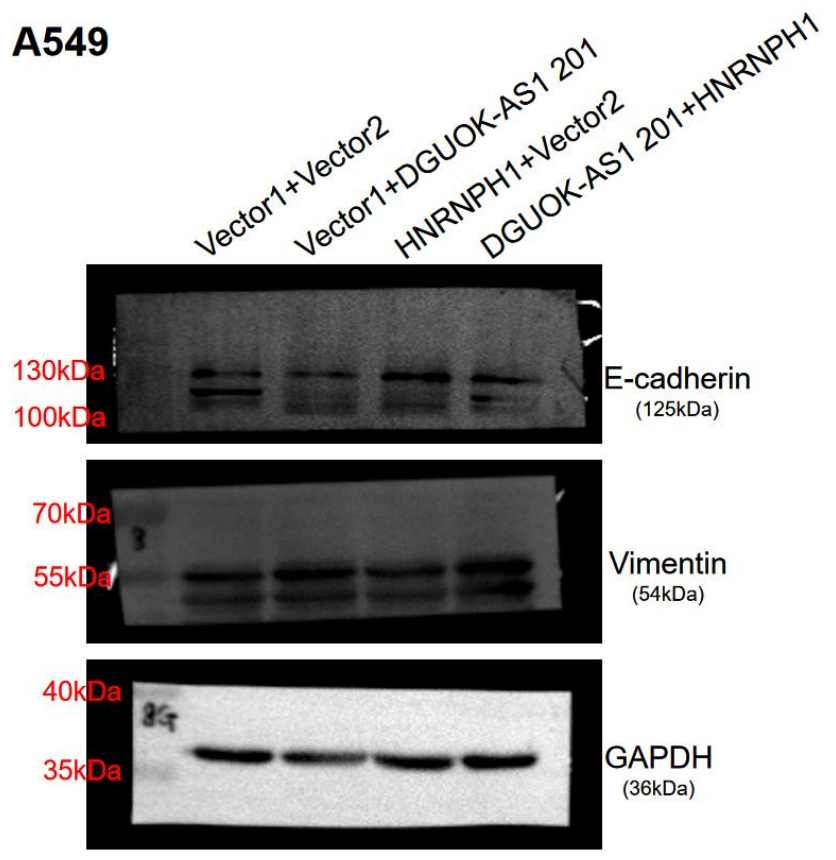

Supplement: Supplementary file 1 [file mmc1.pdf]
